# Comparison of Pleated and Rippled β‑Sheet Assembly of Sequence Isomers of an Amphipathic Self-Assembling Peptide

**DOI:** 10.1021/acs.biochem.6c00035

**Published:** 2026-03-20

**Authors:** Christopher W. Jones, Jianping Chen, Rishab Panda, Sharareh Jalali, Loren P. Cardani, Yahui Guo, Ian M. Arnold, Cristiano L. Dias, Bradley L. Nilsson

**Affiliations:** † Department of Chemistry, 6927 University of Rochester, Rochester, New York 14627-0216, United States; ‡ Department of Physics, 5965 New Jersey Institute of Technology, Newark, New Jersey 07102-1982, United States; § Materials Science Program, University of Rochester, Rochester, New York 14627-0166, United States

## Abstract

Supramolecular β-sheet peptide nanomaterials are of critical interest due to their relevance in amyloid disorders and are increasingly valued for applications in regenerative medicine, tissue engineering, and antimicrobial design. Amphipathic peptides, particularly those with alternating hydrophobic and hydrophilic residues, readily form amyloid-like pleated β-sheet fibrils. It has been demonstrated that the amino acid sequence order of isomeric peptides dramatically influences the self-assembly propensity of the resulting sequences as well as the morphology of the assembled pleated β-sheet nanomaterials. This was substantiated by our previous investigations of the peptides Ac-(FKFE)_2_-NH_2_ (L1), Ac-(FK)_2_(FE)_2_-NH_2_ (L2), Ac-KE­(F)_4_KE-NH2 (L3), Ac-(KFFE)_2_-NH_2_ (L4), and Ac-FF­(KE)_2_FF-NH_2_ (L5). Recently, interest in the Pauling and Corey rippled β-sheet motif, composed of coassembled enantiomeric l- and d-peptides in which the l- and d-enantiomers are organized in an alternating fashion, has been revitalized, although understanding of the rippled β-sheet fold lags far behind that of the naturally occurring pleated β-sheet. Herein, we interrogate the scope of rippled β-sheet formation by extending our previous study of the L1–L5 peptides to enantiomeric mixtures of these sequences to understand the effect of sequence order on rippled β-sheet formation. These integrated experimental and computational studies confirm that enantiomeric mixtures of these peptides have a significantly higher propensity to coassemble into putative rippled β-sheets than single enantiomers have to self-assemble into pleated β-sheets under the same solvent and concentration conditions. These findings extend our understanding of the rippled β-sheet motif and highlight the potential to exploit stereochemically diverse peptides in the design of next-generation biomaterials.

## Graphical abstract

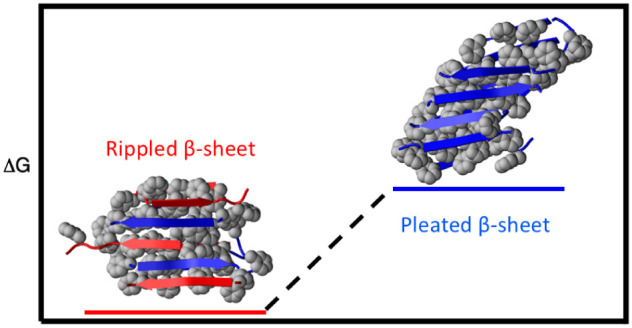

## Introduction

Self-assembled β-sheet peptide nanoassemblies have garnered significant interest due to their relevance to amyloid disorders and for their potential as modifiable supramolecular biomaterials.^1–5^ These amyloid aggregates and designed supramolecular peptide assemblies are composed of pleated β-sheets^6^ organized into laminated cross-β architectures.^7–9^ In canonical pleated β-sheets, neighboring peptides in an extended β-strand conformation interact through backbone hydrogen bonding and are aligned with side chains oriented in cross-strand eclipsed geometries that provide the characteristic pleated appearance (Figure 1A).^10^ Shortly after their elucidation of the pleated β-sheet structure,^6^ Pauling and Corey hypothesized a second type of β-sheet, the rippled β-sheet.^11^ They proposed that equimolar mixtures of enantiomeric l- and d-peptides would form structurally distinct β-sheets in which the l- and d-peptide strands were organized in an alternating arrangement (Figure 1B). In the theorized rippled β-sheets, neighboring cross-strand α-carbons and side chain groups exist in a staggered conformation. The side chain groups thus form a zigzag or “rippled” pattern that is distinct from the pleated pattern of the naturally observed pleated β-sheet. The hypothetical rippled β-sheet is not expected to occur in nature, and only recently has the rippled β-sheet motif been observed in designed systems.^10^

**Figure 1:**
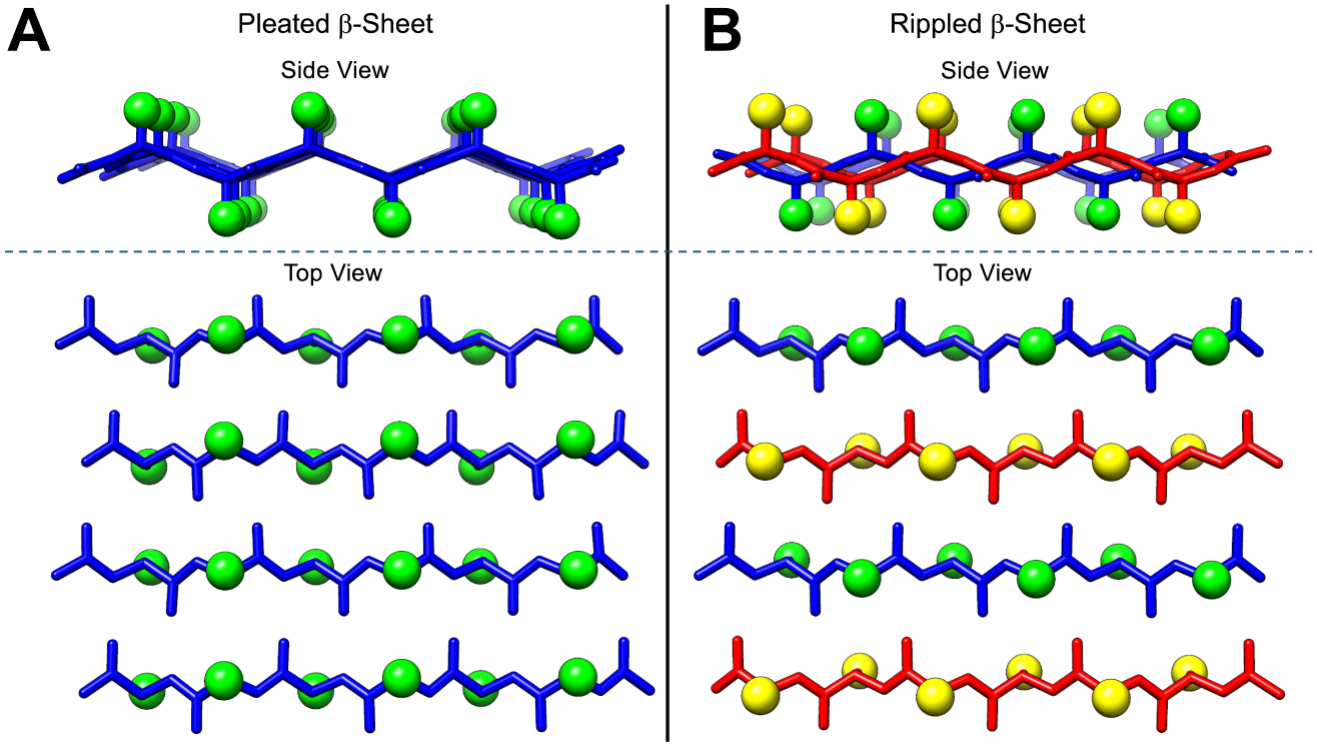
Structural representations of pleated and β-sheet systems. (A) Side and top-view of a pleated β-sheet composed of l-enantiomer peptides (blue) with side chain groups shown as green spheres. (B) Side and top-view of a rippled β-sheet with alternating l- (blue) and d-enantiomer (red) peptides. The side chain groups belonging to the d-strands are depicted as yellow spheres.

The rippled β-sheet motif has remained largely unexamined since it was first proposed by Pauling and Corey in 1953.^11^ Several studies proposed that polyglycine^12,13^ and racemic mixtures of poly-l- and poly-d-lysine^14^ or poly-l- and poly-d-benzyl-aspartate^15^ adopt rippled and not pleated β-sheet structures. More recently, we and others have examined racemic mixtures of self-assembling peptides and reported data consistent with the formation of rippled β-sheets.^10^ Schneider and coworkers demonstrated that racemic mixtures of their hairpin-templated (VK)_n_ MAX1 peptide assemble into supramolecular fibril hydrogels with different emergent viscoelasticity than the single enantiomer pleated β-sheet nanofibrils.^16,17^ We have shown that equimolar mixtures of enantiomers of the amphipathic KFE8 peptide (Ac-(FKFE)_2_-NH_2_) assemble into fibrils that have distinct morphology^18^ from the pleated β-sheet nanoribbons and nanotubes^19–22^ and that these putative rippled β-sheet assemblies also have unique emergent hydrogel properties and stability to proteolytic degradation relative to the corresponding single-enantiomer pleated β-sheet assemblies.^23^ We have also shown that racemic mixtures of the amyloid-β 16–22 fragment (Aβ(16–22)) have distinctive assembly properties relative to single enantiomer solutions consistent with rippled β-sheet assembly.^24^ Raskatov and coworkers have demonstrated that several amyloid-derived peptides also favor rippled β-sheet formation from racemic mixtures.^25–28^ Significantly, Schneider and Raskatov have obtained high-resolution evidence of the rippled β-sheet structure from crystallizing peptide systems.^29–32^ These examples are now complemented by a growing body of evidence for rippled β-sheet formation.^33–35^ Despite this mounting confirmation of the Pauling and Corey prediction of the rippled β-sheet fold, others have shown that some enantiomeric mixtures of l- and d-peptides do not appear to form rippled β-sheets but rather favor self-sorting into all-l and all-d pleated β-sheets.^36–39^ Thus, the scope of rippled β-sheet formation is not completely understood. Additional studies are necessary to expand our understanding of rippled β-sheet versus pleated β-sheet formation in enantiomeric peptide mixtures.

Accordingly, we sought to investigate the sequence scope of rippled versus pleated β-sheet formation in closely related sequence isomers of the amphipathic KFE8 peptide. KFE8 (Ac-(FKFE)_2_-NH_2_, herein referred to as **L1**) is an oft-studied self-assembling peptide composed of alternating hydrophobic/hydrophilic amino acids. We have previously compared the self-assembly of **L1** with isomeric peptides that have identical amino acid constituents but differing sequence patterns, including Ac-(FK)_2_(FE)_2_-NH_2_ (**L2**), Ac-KE­(F)_4_KE-NH_2_ (**L3**), Ac-(KFFE)_2_-NH_2_ (**L4**), and Ac-FF­(KE)_2_FF-NH_2_ (**L5**).^40^ These varied sequence patterns range in similarity to **L1**, with **L2** retaining the alternating hydrophobic/hydrophilic amino acid pattern but altering the order of the hydrophilic residues, **L3** clusters the hydrophobic Phe residues in the center of the peptide, and **L4** and **L5** have pairs of Phe residues in differing symmetrical positions. The comparative self-assembly propensity of these peptides was assessed experimentally, revealing that **L1** and **L2** have a strong self-assembly propensity, **L3** has an intermediate self-assembly propensity, and **L4** and **L5** have significantly lower self-assembly propensity. These experimental studies were complemented by computational molecular dynamics studies, which confirmed the impact of sequence order on relative self-assembly ability for these peptide sequences.^41^ Collectively, this work provided significant insight into the relationship between amino acid sequence and the pleated β-sheet self-assembly characteristics of these sequences.

Herein, we revisit these sequences to interrogate the impact of amino acid sequence order on pleated versus rippled β-sheet assembly. In an earlier study of rippled β-sheet assembly from racemic mixtures of **L1** (equimolar mixture of **L1** with its enantiomeric d-sequence, **D1**) we discovered that rippled β-sheet formation from **L1**/**D1** mixtures was enthalpically favored over pleated β-sheet formation of **L1** by ∼9 kcal mol^–1^.^18^ Based on this observation, we hypothesized that this increased propensity for racemic mixtures of **L1** to coassemble into rippled β-sheets compared to self-assembly of the single enantiomer into pleated β-sheets would be reflected across the complete series of **L1**–**L5** sequences with their corresponding **D1**–**D5** enantiomers. Accordingly, we herein report the outcome of these integrated experimental and computational studies. Experimentally, all self-assembling peptides have associated critical concentrations (C_r_) at which self- or coassembly occurs, reflective of the thermodynamics of assembly for the system. We have analyzed the concentration dependence of pleated and rippled β-sheet formation for peptides **L1**–**L5** (pleated β-sheets) and **L1**–**L5**/**D1**–**D5** mixtures (rippled β-sheets) and have found that in all cases, coassembly of enantiomeric mixtures of these peptides into putative rippled β-sheets occurs at significantly lower critical concentrations than single enantiomer self-assembly into pleated β-sheets at identical total peptide concentrations. Computational molecular dynamics simulations provide complementary insight into these experimental observations. The details of these studies described herein significantly expand our understanding of the paradigms of pleated and rippled β-sheet assembly as a function of peptide sequence.

## Experimental Procedures

### Materials

Fmoc-Rink Amide OctaGel resin (Aapptec, 120–150 mesh, 1% DVB, 0.6 mmol/g, Louisville, KY, USA), *N*,*N*-dimethylformamide (DMF, Thermo Fisher Scientific), 1-hydroxybenzotriazole (HOBt, Aapptec), *O*-(benzotriazol-1-yl)-*N*,*N*,*N*′,*N*′-tetramethyluronium hexafluorophosphate (HBTU, Aapptec), *N*,*N*-diisopropylethylamine (DIPEA, Oakwood), fluorenylmethoxycarbonyl (Fmoc)-protected amino acids (including Fmoc-Phe (Aapptec), Fmoc-Lys­(Boc) (Aapptec), Fmoc-Glu­(O^t^Bu) (Aapptec), Fmoc-Phe with carbonyl 13C-label (Cambridge Isotope Laboratories)), piperidine (Thermo Fisher Scientific), acetic anhydride (Oakwood), trifluoroacetic acid (TFA, Oakwood), triisopropylsilane (Oakwood), acetonitrile (Thermo Fisher Scientific), deuterium oxide (Cambridge Isotope Laboratories), deuterium chloride (Sigma-Aldrich) were purchased commercially from the indicated sources and used without further purification.

### Peptide Synthesis, Purification, and Characterization

Peptides were manually synthesized as C-terminal amides on Fmoc-Rink Amide OctaGel resin using standard Fmoc-protected solid-phase peptide synthesis techniques. Fmoc-protected amino acids were used in 4× excess, dissolved in 8–10 mL of DMF, and activated by addition of 4 molar equivalents of HOBt and HBTU with 300 μL of DIPEA. These Fmoc-protected amino acid activation solutions were allowed to stand for 20 min. After 20 min, activated Fmoc-protected amino acids were added to the resin and allowed to couple for 1 h with gentle mixing. After filtration and washing of the resin, Fmoc-deprotection was performed with 20% v/v piperidine/DMF. This process of deprotection and coupling was repeated for the entire peptide sequence. After coupling the last amino acid, peptides were acetylated using 20% v/v acetic anhydride/DMF. After each step (deprotection, coupling, acetylation), the resin was rinsed thoroughly with 3–6 reaction vessel volumes of DMF. Peptides were deprotected and cleaved from the resin using standard cleavage conditions of TFA, triisopropylsilane, and water (95:2.5:2.5 v/v) for 1 h. The cleavage solution was collected, and the resin was subjected to another round of cleavage for 1 h. The cleavage solutions were combined, concentrated to ∼5.0 mL, and the synthetic peptides were precipitated by addition of cold (−78 °C) diethyl ether, followed by collection after centrifugation. The supernatant was decanted, and the pellet was resuspended in another volume of cold diethyl ether, followed by centrifugation and decantation of ether. The resulting peptide pellet was dissolved in 60% v/v acetonitrile/water with 0.1% TFA, frozen, and lyophilized overnight. Following lyophilization, peptides were dissolved in 60% v/v acetonitrile/water with 0.1% TFA or DMSO. Peptides were then purified via preparatory scale reverse-phase high-performance liquid chromatography (RP-HPLC) on an Interchim PuriFlash 4125 instrument equipped with a Phenomenex Gemini column (10 μm C18 Axia, 50 × 250 mm, Phenomenex, Torrance, CA, USA) heated to 55 °C, and injected using a 2 mL sample loop (see Figures S1–S16 in Supporting Information for HPLC traces of purified peptides). A gradient of acetonitrile and water with 0.1% TFA was used as the mobile phase at a flow rate of 100 mL min^–1^. The eluent was monitored using UV absorbance at 215 and 254 nm for fraction collection (see Tables S1–S6 in Supporting Information for peptide retention times and HPLC purification gradients). d-peptides and labeled peptides were purified with the same gradient that their corresponding l-peptides were purified with. Peptide purity was confirmed using analytical RP-HPLC using a Phenomenex Gemini column (5 μm, C18, 110 Å, 4.6 × 250 mm) heated to 40 °C on a Shimadzu LC-2010A instrument (see Tables S7–S8 in Supporting Information for peptide retention times and analytical HPLC gradients). After purification, peptide identity was confirmed using matrix-assisted laser desorption ionization-time-of-flight (MALDI-TOF) mass spectrometry (Shimadzu Axia Performance MALDI, Shimadzu Corporation, Nakagyo-ku, Kyoto, Japan). See Figures S17–S32 in Supporting Information for MALDI-TOF spectra and Table S9 for the calculated and observed m/z for peptides.

### Peptide Self-Assembly

Purified peptides were dissolved in 60% v/v acetonitrile/water with 0.1% TFA to prevent assembly, enabling RP-HPLC analysis. Concentrations of synthesized peptides were determined by correlation of the HPLC peak area of a specified volume injection of the acetonitrile/water stock solution to a RP-HPLC concentration curve calibrated by amino acid analysis (UC Davis, see Figures S33–S37 in Supporting Information for calibration curves). Concentration determinations were performed on the exact column on which the concentration curve was constructed under the identical mobile phase conditions used to construct the concentration curve. Once the concentration of each stock solution was determined, peptides were distributed in required quantities for their respective analyses, frozen, and lyophilized. Assembly of each peptide was induced via dissolution of lyophilized powders in unbuffered water (for use in TEM experiments) or deuterium oxide (for use in FTIR experiments). Upon addition of solvent, these samples were subjected to three cycles of manual mixing (1 min) and sonication (5 min). For coassembly of enantiomeric mixtures, the l- and d-peptides were mixed while dissolved in acetonitrile/water to minimize self-assembly of the individual peptides prior to mixing of the enantiomers. These racemic mixtures were frozen, lyophilized, and dissolved in water to initiate assembly.

### Fourier Transform Infrared (FTIR) Spectroscopy

FT-IR spectra were obtained at 0, 24, and 96 h after peptide assembly using a PerkinElmer 8400 FT-IR spectrometer (See figures in the Results and Discussion section and Figures S38–S47 in Supporting Information for time point comparison of each peptide assembly). Residual water and TFA were removed through two cycles of anion exchange by lyophilization from 60% v/v D_2_O/acetonitrile with 1% DCl and 60% v/v D_2_O/acetonitrile with 0.1% DCl. Lyophilized peptides were dissolved and assembled in D_2_O as described above. An aliquot (∼100 μL) of each peptide was injected into a 25 mm × 4 mm CaF_2_ sandwich plate (International Crystal Laboratories). Background and absorbance measurements were taken using the Happ-Genzel method from 1500–1800 cm^–1^ with a 1 cm^–1^ resolution for 8 scans.

Python was employed using custom scripts based on NumPy, SciPy, and Matplotlib to assign structural contributions of random coil and β-sheet structures in FT-IR spectra (see Figures S48–S67 in Supporting Information). To quantify the spectral contributions, the amide I band was modeled as the combination of two Lorentzian functions representing a dominant peak and a secondary shoulder peak. Peak center positions were fixed at 1620 cm^–1^ and 1650 cm^–1^, corresponding to the presence of β-sheet and random coil structures, respectively. FTIR spectra were baseline-corrected, smoothed, and normalized before analysis. A constant offset term was included to account for baseline correction. Nonlinear least-squares fitting was performed using the curve fit function from SciPy. Initial parameter estimates were chosen conservatively to ensure robust convergence. Peak amplitudes were constrained to positive values, and Lorentzian half-widths at half-maximum (HWHM) were restricted to physically reasonable ranges for the amide I bands. Individual peak components were reconstructed from the optimized parameters, and the total fitted spectra were obtained by the summation of all components and the baseline. Peak areas were calculated analytically using the integral of a Lorentzian function (area = a × π × γ), allowing direct comparison of the relative contributions of each spectral component. Fractional areas were reported as percentages of the total amide I signal (for details regarding peak fitting integration and peak fitting code, see Supporting Information).

### Circular Dichroism Spectroscopy

Circular dichroism (CD) spectra were recorded using a Jasco-815 circular dichroism spectrometer with a 0.1 mm path-length quartz cuvette (see Figures S68–S87 in Supporting Information for CD spectra). Scans were performed at 25 °C in duplicate, covering the wavelength range of 190–260 nm. The scanning parameters included a 1.0 nm data pitch, 1.0 nm bandwidth, and a scanning speed of 100 nm/min.

Background correction was applied to the spectra by subtracting the spectra of water alone. Peptides were assembled and diluted to a working concentration to ensure the High Tension (HT) voltage value was within the required limits (see Table S10 for assembly concentrations and working concentrations for CD spectroscopy). This was done to ensure good data quality. Ellipticity (θ) was converted into molar ellipticity ([θ]) using the formula:

1 $$\left[\theta \right]=\frac{\theta }{10\;x\;c\;x\;n\;x\;l}$$

Where [θ] has units of deg cm^2^ dmol^–1^, θ is the measured ellipticity in millidegrees, c is the molar concentration of the peptide, n is the number of amino residues in the peptide, and l is the path length of the cuvette in cm.

### Transmission Electron Microscopy

An aliquot of 5 μL of preassembled peptide sample was deposited onto the surface of a 200-mesh carbon-coated copper grid and allowed to stand for 15 s. Without disrupting the integrity of the grid, the sample was then carefully removed using filter paper via capillary action. Samples were diluted to 0.2 mM before being added to the TEM grid to reduce fibril concentration on the grid. Immediately after sample removal, the grids were stained by depositing 5 μL of 5% uranyl acetate onto the surface of the grid; the stain was allowed to sit for 2 min. This was followed by careful removal of the stain solution by filter paper via capillary action. The stained grids were air-dried before storage and imaging. To obtain the electron micrographs of each peptide assembly, a Hitachi 7650 transmission electron microscope in high-contrast mode was used at an accelerating voltage of 80 kV. Fibril dimensions were measured using the software program ImageJ. To obtain reliable representative dimensions, 100 measurements were performed across unique fibers.

### Sedimentation Analysis

Critical aggregation concentrations (C_r_) were measured using a modified Wetzel protocol.^42^ Peptide assembly samples were prepared to have a total volume of 300 μL and assembled at a total peptide concentration at which complete β-sheet structures were observed during FTIR analysis. If β-sheet structures were not observed at the concentrations tested, samples were assembled and analyzed at 4 mM. **L1**, **L2**, **L1/D1**, **L2/D2**, and **L3/D3** were assembled at 1 mM, whereas **L3**, **L4**, **L5**, **L4/D4**, and **L5/D5** were assembled at 4 mM to ensure assembly. Samples were dissolved in 300 μL of water and allowed to assemble for 24 h. They were then pipetted into ultracentrifuge tubes and centrifuged at 436,000 × g for 90 min at 17 °C to separate large aggregates from unassembled “monomeric” species. “Monomeric” peptides may not be strictly monomer but are not part of stable aggregate species. The supernatant was then carefully removed, and the concentration of monomer (C_r_) was determined by injection of a known volume of the supernatant onto an analytical HPLC instrument (specific instrument and column as described above) and correlating the peptide peak area to an HPLC concentration curve. This modified Wetzel protocol was applied to each sample in triplicate and analyzed on analytical HPLC in triplicate to ensure data integrity. Error is reported as the standard deviation of the average value.

The C_r_ values can be used to make thermodynamic comparisons between the respective pleated and rippled β-sheet systems. Specifically, the C_r_ values can be used to determine the net change in free energy (ΔG) for each peptide assembly, enabling a thermodynamic comparison for pleated and rippled β-sheets. For each assembly, the following simplified equilibrium is assumed:

2 $$fibril_{n}+\mathrm{monomer}⇌fibril_{n+1}$$

From this equilibrium state, the association constant, K_a_, for addition of monomer to fibril is defined as:

3 $$K_{a}=\frac{\left[fibril_{n+1}\right]}{\left[fibril_{n}\right]\left[\mathrm{monomer}\right]}$$

The K_a_ value for each peptide system can be determined from the experimental C_r_ value, which approximates [monomer]. Since [fibril_n_] is essentially equal to [fibril_n+1_], K_a_ and C_r_ have a reciprocal relationship:

4 $$K_{a}=\frac{1}{C_{r}}$$

Thus, the K_a_ values, can be used to calculate ΔG for each assembly condition using the standard equation ΔG = −RTln­(K_a_). In this equation, R is the inert gas constant (1.987 cal (mol K)^−1^), and T is the temperature in Kelvin (298.15 K). ΔΔG values were then calculated for each corresponding pleated and rippled β-sheet system to compare the thermodynamic preference of pleated β-sheet self-assembly to rippled β-sheet enantiomer coassembly.

### Molecular Dynamics Simulation and Analysis

All-atom molecular dynamics simulations were performed using GROMACS 2021, with atomic interactions described by the AMBER99SB-ILDN force field.^43,44^ For each of the five peptide sequences studied here, simulations were carried out by placing 5 × l-enantiomers and 5 × d-enantiomers in a box of size 10 × 10 × 10 nm^3^, which was filled with approximately 32,000 TIP3P water molecules.^45^ This corresponds to a peptide concentration of 16 mM. These systems with more than 100,000 atoms were simulated for 3 μs.

Initially, the energy of the system was minimized using the steepest descent algorithm until the maximum computed force fell below 1000 kJ mol^–1^ nm^–1^ or a maximum number of 5000 steps was reached. This was followed by short (500 ps) NVT and NPT equilibration simulations.^46^ Temperature was maintained at 350 K using the Bussi velocity-rescaling thermostat with a coupling time constant (τ_t_) of 1 ps, applied to both solvent and peptides.^47^ The use of high temperatures in simulations was shown previously to speed up the dynamics of peptide aggregation in homochiral simulations of **L1**, **L3**, and **L4** peptides, enabling the formation of cross-β structures within a 2–4 μs.^41^ Pressure was maintained at 1 atm using an isotropic Parrinello–Rahman barostat with a coupling time constant (τ_p_) of 2 ps.^48^ Long-range electrostatic interactions were treated using the Particle Mesh Ewald (PME) method with a real-space cutoff of 1.2 nm.^49,50^ van der Waals interactions were smoothly force-switched to zero over the range 1.0 and 1.2 nm. All bonds involving hydrogen atoms were constrained using the LINCS algorithm, and periodic boundary conditions were applied in all three dimensions.^51,52^

## Results and Discussion

### Experimental Design

The purpose of this study was to gain insight into the relative propensity of closely related sequences to self-assemble into pleated β-sheet structures compared to the coassembly of racemic mixtures of the same sequences into multicomponent rippled β-sheet structures. We revisited peptides **L1**–**L5** and the corresponding enantiomers (Table 1) for these studies.^40^ These peptides have identical amino acid content but with differing sequence order. In our prior work, we found that the self-assembly of these peptides into pleated β-sheets occurs most effectively for the **L1** and **L2** peptides, which self-assemble rapidly into nanoribbons and nanotubes (**L1**) and nanofibrils (**L2**) even at low concentrations (0.2 mM). In comparison, the **L3** peptide with the Phe residues clustered in the central position of the peptide self-assembles into broad, polymorphic nanotapes only at higher concentrations (1 mM). Finally, the **L4** and **L5** peptides were found to have a low self-assembly propensity, failing to show dominant β-sheet spectroscopic signatures even at 1 mM concentrations. **L4** showed no evidence of β-sheet assemblies by TEM imaging even at a higher 1 mM concentration. TEM imaging of the **L5** peptide solutions showed no significant β-sheet assemblies at low concentrations (0.2 mM), and only minimal spherical aggregates with sparse fiber structures at higher 1 mM concentrations. Thus, we were able to classify the pleated β-sheet self-assembly propensity of these sequences as following the order **L1** ≈ **L2** > **L3** ≫ **L4** ≈ **L5**. Subsequently, we interrogated pleated β-sheet self-assembly of these peptides computationally by conducting long molecular dynamics simulations, which confirmed the experimental study, showing that **L1** and **L2** have strong self-assembly properties, followed by **L3**, with **L4** and **L5** having significantly lower self-assembly efficiencies.^41^ These data support the privileged self-assembly facility of amphipathic peptides with alternating hydrophobic/hydrophilic amino acid sequences due to the ability of these peptides to form a pleated β-sheet that sequesters hydrophobic and hydrophilic functionality to opposite faces of the β-sheet, facilitating the formation of bilayer structures.^20^ This study also demonstrates that larger patches of hydrophobic surface area clusters have a greater self-assembly propensity than peptides with lower hydrophobic cluster area.

**Table 1:** Peptide Sequences^a^

Peptide Designation	Peptide Sequence
**L1**	Ac- **F** K **F** E **F** K **F** E-NH_2_
**D1**	Ac- **f** k **f** e **f** k **f** e-NH_2_
**L2**	Ac- **F** K **F** K **F** E **F** E-NH_2_
**D2**	Ac- **f** k **f** k **f** e **f** e-NH_2_
**L3**	Ac-KE **FFFF** KE-NH_2_
**D3**	Ac-ke **ffff** ke-NH_2_
**L4**	Ac-K **FF** EK **FF** E-NH_2_
**D4**	Ac-k **ff** ek **ff** e-NH_2_
**L5**	Ac- **FF** KEKE **FF** -NH_2_
**D5**	Ac- **ff** keke **ff** -NH_2_

a Amino acids with l-stereochemistry are capitalized and amino acids with d-stereochemistry are shown in lower case. Hydrophobic Phe residues are underlined in bold font to emphasize the differing sequence order for each of the peptides.

In this work, we reevaluate these isomeric peptide sequences and their enantiomers to compare the relative propensity for pleated β-sheet self-assembly of **L1**–**L5** to the coassembly of rippled β-sheets from racemic mixtures of these peptides with their d-enantiomers. In our initial report of rippled β-sheet formation, we conducted isothermal titration calorimetry measurements of modified **L1** sequence variants by titrating cationic l-Ac-(FK)_4_-NH_2_ with either l-Ac-(FE)_4_-NH_2_ or d-Ac-(FE)_4_-NH_2_ and observed that coassembly of the charge complementary enantiomers into putative rippled β-sheets was enthalpically more favorable than self-assembly of the two l-isomers into pleated β-sheets by ∼9 kcal mol^–1^.^18^ We hypothesized that the enthalpic advantage for rippled β-sheet over pleated β-sheet assembly is due to the less sterically constrained packing architecture of the rippled β-sheet compared to the pleated β-sheet (Figure 1). Further, based on this hypothesis, we expected that rippled β-sheet formation should therefore be more favorable for a broad range of self-assembling peptide sequences. To test this hypothesis, we prepared the peptides in Table 1 by solid-phase peptide synthesis and compared the self-assembly of the l-enantiomers to the coassembly of racemic l/d-mixtures at identical total peptide concentrations to determine if rippled β-sheet formation was observed to occur more favorably than the corresponding formation of pleated β-sheets.

### Characterization of β-Sheet Secondary Structure by Fourier Transform Infrared Spectroscopy

For these studies, we repeated our previously reported analyses of the self-assembly of the **L1**–**L5** peptides and compared these to the assembly of equimolar mixtures of **L1**/**D1**, **L2**/**D2**, **L3**/**D3**, **L4**/**D4**, and **L5**/**D5**. In our previously published work on the pleated β-sheet self-assembly of **L1**–**L5**, we assessed self-assembly at 0.2 mM and 1.0 mM concentrations.^40^ Herein, we utilize higher peptide concentrations of 1.0 mM, 2.0 mM, and 4.0 mM to increase the likelihood of self-assembly for those sequences that are less prone to self-assemble, particularly **L4** and **L5**. Coassembly studies of racemic mixtures into putative rippled β-sheets was undertaken at the same total peptide concentrations, with half the peptide concentration contributed by the l-stereoisomer and the other half contributed by the d-enantiomer.

To compare the efficiency of pleated β-sheet self-assembly of l-isomers to the rippled β-sheet coassembly of enantiomeric l/d-peptide mixtures, we first utilized Fourier transform infrared (FTIR) spectroscopy to measure the spectroscopic emergence of β-sheet secondary structure in solutions of the isomeric peptide sequences in Table 1. The appearance of characteristic β-sheet secondary structure correlates strongly with supramolecular self-assembly for these peptides. Specifically, FTIR provides structural insights for peptide systems by examination of the amide I vibrational band from ∼1600–1700 cm^–1^. β-Sheet structures typically exhibit a peak near 1620 cm^–1^, whereas random structures appear at ∼1650 cm^–1^.^53,54^ Additionally, a minor peak of high frequency above 1670 cm^–1^ is often associated with an antiparallel β-sheet strand orientation.^55,56^

We also employed circular dichroism (CD) as a complementary secondary structure analysis method to interrogate pleated and rippled β-sheet assembly (see Figures S68–S87 in Supporting Information for CD spectra). Along with FTIR, CD has served as a vital structural tool for secondary structure characterization for discrete proteins and for supramolecular peptide assemblies. The classical CD β-sheet signal for proteins and peptides features a characteristic negative band at 218 nm and a positive band at 195 nm.^57^ However, the morphological and spectral diversity found in β-sheet-rich protein structures significantly limits the estimation of structural elements via conventional CD.^58^ Advances in predictive algorithms have been developed to overcome this challenge. Likewise, the structures of supramolecular peptide assemblies are intrinsically diverse and complex and often display unconventional spectral effects in CD spectra. This is particularly evident in the CD spectrum of KFE8 (L1), in which the complex morphological nanoribbon and nanotube characteristics give rise to CD signatures that do not comport with typical β-sheet structures. We have previously reported that CD spectra of L1 pleated β-sheet assemblies feature a strong nonstandard minimum at 204 nm in addition to the expected minimum at 215 nm.^18,59,60^ Conticello and Egelman have also reported a nearly identical L1 spectrum and further shown that, after heating and cooling, the CD spectrum switches to positive ellipticity while nonetheless still maintaining β-sheet secondary structure as determined by cryo-EM reconstruction studies.^20^ These nonstandard CD spectra arise from the chiral L1 nanoribbons and nanotubes which also interact with plane-polarized light in CD experiments. Further, the mixtures of l- and d-enantiomers in putative rippled β-sheets often exhibit exceptionally weak CD signals, and this is also true in the l/d mixtures reported herein (see **Figures S68–S87** and **Table S10** in Supporting Information). Thus, the ambiguity of the unique CD signatures that these types of β-sheet assemblies display make FTIR analysis in the amide I region (1800–1500 cm^–1^) a more reliable and unambiguous assessment of β-sheet secondary structure in these systems. Nonetheless, we report the CD data for each peptide assembly in the interest of comprehensive characterization.

Self-assembly was promoted by dissolution of the peptides at the desired concentrations into unbuffered water. The pH of all solutions was between 2 and 4 due to residual trifluoroacetic acid from HPLC purification. Purified peptides were first dissolved in acetonitrile/water mixtures to disfavor self-assembly, and peptide concentrations were precisely determined by correlation to HPLC peptide concentration curves. The peptide was then divided into aliquots of the desired amounts, frozen, and concentrated by lyophilization. These lyophilized samples were dissolved in water at the desired concentration to initiate self-assembly. For FTIR experiments, each sample was first prepared by repeated dissolution in D_2_O with DCl followed by freezing and lyophilization in order to exchange the triflate counterions, which have IR signatures in the amide I region, for chloride counterions. FTIR samples were dissolved in D_2_O for analysis to avoid interference from water in the amide I region of the IR spectrum. FTIR analysis for each sample was performed after 0 (within an hour after dissolution in water), 24, or 96 h after initiation of self-assembly.

Solutions of **L1**–**L5** were first analyzed at 1 mM concentrations. The observed outcomes of these analyses were consistent with our initial report.^40^ FTIR spectra of solutions of **L1** and **L2** at 1 mM concentrations showed strong IR peaks at 1618 cm^–1^, consistent with pleated β-sheet self-assembly (Figure 2A). Solutions of **L3** displayed peaks at both 1623 cm^–1^ and 1646 cm^–1^, indicative of a mixture of **L3** self-assembled into pleated β-sheets and random aggregates (Figure 2A). To estimate the relative ratios of β-sheet and random structure, we applied a Lorentzian function to the amide I region of the spectra. Based on this data-fitting analysis, the FTIR spectrum of **L3** indicates 78% random coil and 22% β-sheet structure at 1 mM peptide concentrations immediately after peptide dissolution, with β-sheet structure increasing to 31% (69% random coil) after 96 h (see Table 2 and Figure S52 in Supporting Information). In contrast, solutions of **L4** and **L5** at 1 mM exhibited only broad peaks at ∼1650 cm^–1^, suggesting that these solutions primarily consist of random aggregates (Figure 2A). The reduced pleated β-sheet self-assembly propensity of the **L3**, **L4**, and **L5** sequence variants compared to the **L1** and **L2** peptides arises from the altered amino acid sequence. Whereas **L1** and **L2** feature the alternating hydrophobic/hydrophilic sequence pattern, **L3**, **L4**, and **L5** are composed of a diphenylalanine motif separated by 0, 1, or 2 pairs of hydrophilic amino acids, respectively. Even though diphenylalanine has been shown to be highly effective at supramolecular self-assembly,^61–65^ the hydrophobic phenylalanine residues in the context of the **L3**, **L4**, and **L5** peptides are now found on opposite faces of the β-strand, thus preventing self-assembly into optimal bilayer architectures.^20^ This result highlights the privileged nature of an alternating sequence pattern of hydrophobic/hydrophilic amino acids in supramolecular pleated β-sheet-forming self-assembly.

**Figure 2:**
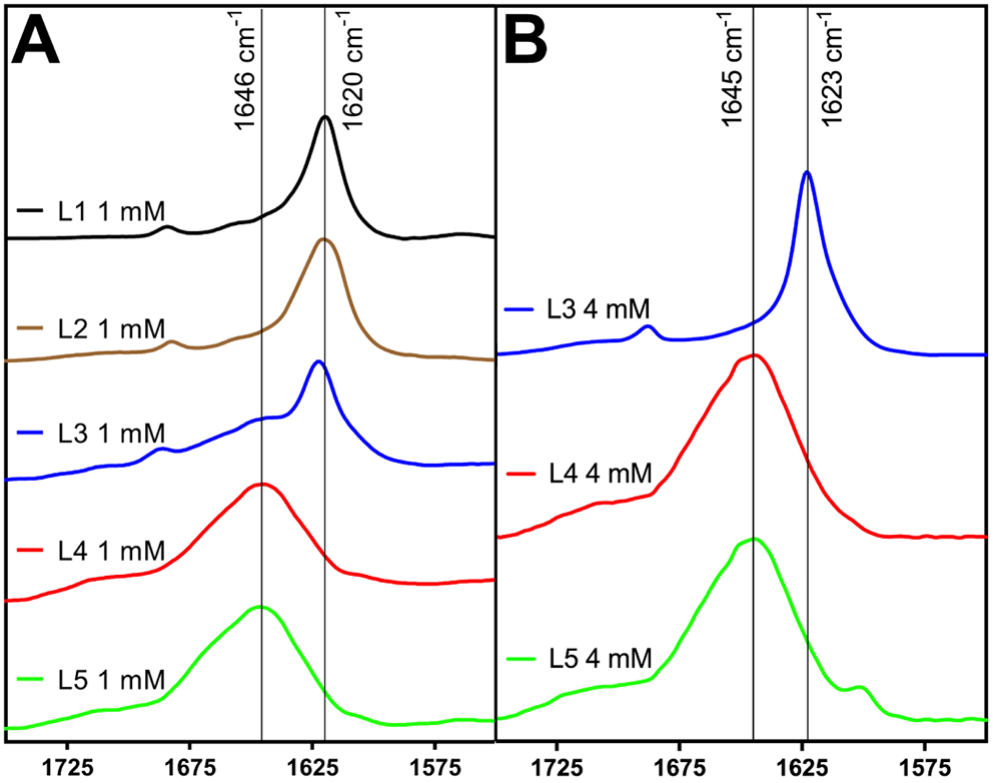
Fourier transform infrared spectra of L1–L5 peptide solutions at 1 mM and 4 mM peptide concentrations. (A) L1, L2, L3, L4, and L5 peptides at a total peptide concentration of 1 mM. (B) L3, L4, and L5 peptides at a total peptide concentration of 4 mM.

**Table 2:** Percent Contributions of Random Coil and β-Sheet Structures in Peptide Assemblies Based on Lorentzian Peak Fitting of the Amide I Region of FTIR Spectra^a^

	0 h		24 h		96 h	
	Random %	β-sheet %	Random %	β-sheet %	Random %	β-sheet %
**L1** 1 mM	22	78	4	96	16	84
L1/D1 1 mM	29	71	0	100	5	95
**L2** 1 mM	15	85	4	96	15	85
L2/D2 1 mM	28	72	37	63	23	77
**L3** 1 mM	78	22	84	16	69	31
**L3** 2 mM	69	31	70	30	72	28
**L3** 4 mM	34	66	30	70	24	76
L3/D3 1 mM	26	74	30	70	27	73
**L4** 1 mM	100	0	100	0	99	1
**L4** 2 mM	100	0	100	0	100	0
**L4** 4 mM	100	0	100	0	100	0
L4/D4 1 mM	78	22	43	57	37	63
L4/D4 2 mM	28	72	25	75	24	76
L4/D4 4 mM	27	73	25	75	30	70
**L5** 1 mM	100	0	100	0	100	0
**L5** 2 mM	100	0	100	0	99	1
**L5** 4 mM	100	0	100	0	100	0
L5/D5 1 mM	98	2	95	5	81	19
L5/D5 2 mM	88	12	76	24	61	39
L5/D5 4 mM	80	20	48	52	37	63

a Peak fitting spectra can be found in Supporting Information, Figures S48–S67.

We next examined the self-assembly of the **L3**, **L4**, and **L5** peptides at higher concentrations to determine approximate critical concentration (C_r_) values at which pleated β-sheet assembly would occur for these sequences. We assessed solutions of **L3**, **L4**, and **L5** at 2 mM and 4 mM concentrations (Figures S42, S44, and S46). Note that in our previous report of the self-assembly of these peptides, we did not assess self-assembly at concentrations higher than 1 mM.^40^ Increasing the concentration of L3 to 2 mM resulted in a slight increase of β-sheet character (Figure S42), with approximately 31% of the secondary structure from β-sheet and 69% from random structure, with negligible changes to β-sheet structure contribution over 96 h (Table 2 and Figure S53). At 4 mM concentrations of **L3**, the random aggregate contribution was dramatically reduced, and the spectrum was dominated by β-sheet structure (Figure 2B and Table 2). In contrast, **L4** and **L5** continued to display predominantly random structure in FTIR spectra at both 2 mM (Figures S44B and S57, and Figures S46B and S63, respectively) and 4 mM (Figure 2B, Figures S44C and S54, and Figures S46C and S64, respectively) concentrations, with no meaningful contributions from β-sheet structure (Table 2). The sequence pattern of **L4** and **L5** significantly limits the formation of β-sheet assemblies of these peptides even at concentrations as high as 4 mM.

We next compared the pleated β-sheet formation of the l-peptides with the putative rippled β-sheet formation of the corresponding racemic l/d mixtures. The racemic l/d-peptide mixtures were assembled under the same solvent and concentration conditions as in the self-assembly experiments, with the total peptide concentration representing the sum of both enantiomers. For example, **L1/D1** was coassembled at a total peptide concentration of 1 mM, consisting of 0.5 mM **L1** and 0.5 mM **D1**. Enantiomers were mixed in acetonitrile/water solutions to prevent self-assembly of the single enantiomers, and the mixtures were then lyophilized and dissolved in water. The mixtures of **L1/D1** and **L2/D2** at 1 mM concentration yielded FTIR spectra nearly identical to their self-assembled counterparts, displaying strong β-sheet peaks at 1618 cm^–1^ (Figure 3A). Interestingly, mixing **L3** with its enantiomer, **D3**, at 1 mM produced an FTIR spectrum that is entirely β-sheet secondary structure, displaying a sharp peak at 1624 cm^–1^ (Figure 3A). This is in sharp contrast to the FTIR spectrum of the **L3** peptide alone, which was a mixture of random structure and β-sheet, with random structure the dominant feature (Figure 2A). The mixed **L3** and **D3** enantiomers thus exhibit a strongly enhanced β-sheet signal compared to the self-assembled **L3** peptide at 1 mM. This observation is consistent with a greater propensity for rippled β-sheet formation compared to pleated β-sheet formation. Likewise, the **L4/D4** mixture at 1 mM shows a minor contribution of 22% β-sheet signal at early time points that evolves to 63% β-sheet after 96 h (Figure 3A and Table 2). The **L5/D5** mixture at 1 mM total peptide concentration was nearly entirely random structure immediately after peptide dissolution (Figure 3A) but showed increased β-sheet content (19%) after 96 h (Figure S65 and Table 2). Collectively, these data indicate that mixtures of enantiomeric peptides have, to varying extents, a much stronger propensity to form β-sheet structures than the single enantiomer solutions, consistent with rippled β-sheet formation being favored relative to pleated β-sheet formation.

**Figure 3:**
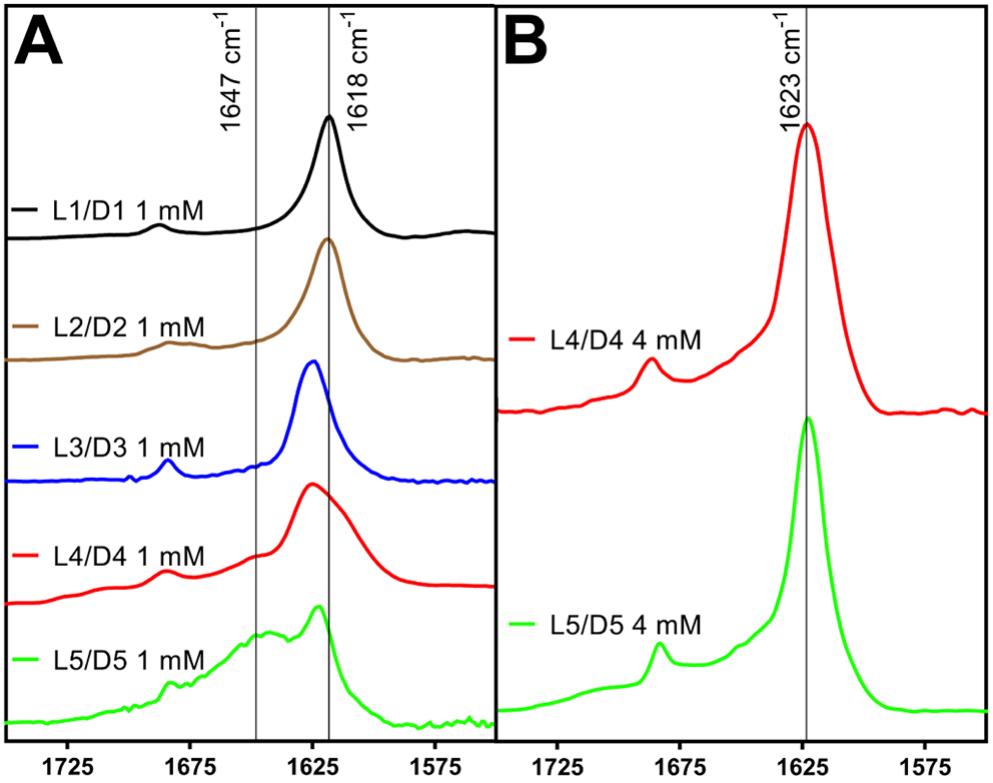
Fourier transform infrared (FTIR) spectra of enantiomeric mixtures of peptides at low (1 mM) and high (4 mM) concentrations. (A) FTIR spectra of racemic mixtures of **L1/D1**, **L2/D2**, **L3/D3**, **L4/D4**, and **L5/D5** at a total peptide concentration of 1 mM. (B) FTIR spectra of racemic mixtures of **L4/D4** and **L5/D5** at a total peptide concentration of 4 mM.

Akin to enantiopure solutions of **L4** and **L5**, we also assessed β-sheet formation in enantiomeric mixtures of **L4/D4** and **L5/D5** at higher concentrations of 2 mM and 4 mM. While solutions of **L4** at 2 mM were random structures (Figures S44B and S57), the **L4/D4** mixtures at 2 mM total peptide were 72% β-sheet immediately after peptide dissolution, increasing only marginally after 96 h (Table 2, Figures S45B and S60). At 2 mM concentration, **L5** solutions were dominated by random structure at all time points (Figures S46B and S63), whereas **L5/D5** mixtures were 12% β-sheet immediately after dissolution and 39% β-sheet after 96 h (Table 2, Figures S47B and S66). Upon increasing the total peptide concentration of L4/D4 and L5/D5 to 4 mM, the associated spectra were strongly β-sheet structure, indicated by the peaks at 1624 cm^–1^ and 1618 cm^–1^, respectively (Figure 3B). The **L4/D4** mixture at 4 mM concentration was ∼70% β-sheet content and remained the same after 96 h (Figure S45C and Figure S61). The **L5/D5** mixture at 4 mM peptide increased in β-sheet content over 96 h from 20% β-sheet immediately after being dissolved in D_2_O to 63% β-sheet (Figure S47C and Figure S67). The formation of putative rippled β-sheets from enantiomeric mixtures of **L4/D4** and **L5/D5** was thus significantly favored over self-assembly of **L4** or **L5** into pleated β-sheets.

### Morphological Comparison of Pleated and Rippled β-Sheet Assemblies by Transmission Electron Microscopy

The results from FTIR analysis of enantiomeric mixtures are consistent with putative rippled β-sheets forming in these systems more favorably than the corresponding enantiomers self-assembling into pleated β-sheets. This was particularly evident based on the strengthened β-sheet signals observed in the enantiomeric mixtures of **L3/D3** (1 mM), **L4/D4** (2 and 4 mM), and **L5/D5** (2 and 4 mM). While data from FTIR analysis is consistent with formation of putative rippled β-sheets based on prior work, additional studies are required to further support rippled β-sheet assembly from the enantiomeric mixtures. While FTIR spectroscopy provides valuable insights into peptide aggregation and secondary structure, this technique alone is insufficient to confirm the presence of ordered β-sheets forming supramolecular structures. In conjunction with FTIR, transmission electron microscopy (TEM) enables validation of the formation of supramolecular self-assembled structures. The morphology of the assemblies provides clues as to whether the enantiomeric mixtures are undergoing coassembly into multicomponent rippled β-sheet structures or are self-sorting into stereouniform pleated β-sheets. For instance, enantiomers that are self-sorting into l- and d-pleated β-sheets will appear as morphologically identical mirror image structures. In our prior work with rippled β-sheets, rippled β-sheet coassembled structures are morphologically distinct from the corresponding pleated β-sheet assemblies.^18,24^ Accordingly, negative-stain TEM was employed to assess the morphology of each of the peptide solutions described above. TEM images were obtained from 1 mM solutions of all peptides, with additional imaging at 4 mM for **L3**, **L4**, **L4/D4**, **L5**, and **L5/D5** to corroborate FTIR results with self-assembled or coassembled structures.

TEM images of all self-assembled pleated β-sheet structures at 1 mM concentrations were consistent with our previous work.^40^ **L1** solutions at 1 mM formed the typical pleated β-sheet helical nanoribbons at early time points, which gradually evolved into nanotubes over time (Figure 4A).^18,20–22,40,59,66,67^ The helical nanoribbons measured 8.9 ± 1.2 nm in width with a helical pitch of 19.5 ± 1.8 nm. The nanotubes that emerge later in the assembly process exhibited similar widths (9.2 ± 1.0 nm). Both assemblies are consistent with structures that have been previously reported based on cryo-EM structural analysis.^20^ In contrast, the putative rippled β-sheet coassembled **L1/D1** fibers were characterized by an irregular morphology and variable widths along the fiber axis that averaged 4.0 ± 0.8 nm (Figure 4B). This is approximately half the width of the **L1** self-assembled structures with dramatically different morphology, consistent with our previous findings that support the formation of rippled β-sheets.^18^

**Figure 4:**
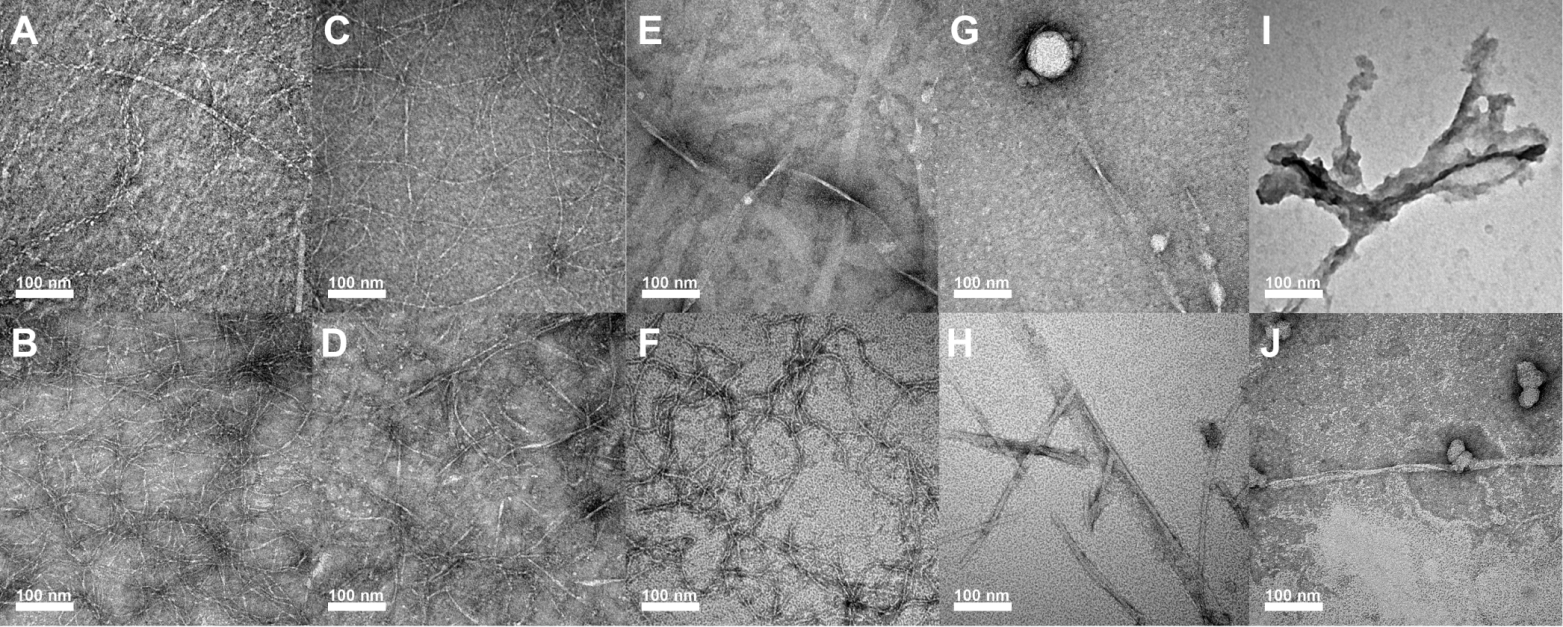
Negative stain transmission electron microscopy (TEM) images of pleated β-sheets of single enantiomer solutions and putative rippled β-sheets of racemic mixtures of the various peptides. **(A) L1** (1 mM), **(B) L1/D1** (1 mM), **(C) L2** (1 mM), **(D) L2/D2** (1 mM), **(E) L3** (1 mM), **(F) L3/D3** (1 mM), **(G) L4** (1 mM), **(H) L4/D4** (1 mM), **(I) L5** (1 mM), **(J) L5/D5** (1 mM).

TEM images of pleated and rippled β-sheet assemblies of **L2** and **L2/D2** likewise revealed morphological differences between the two systems. TEM analysis of the **L2** peptide revealed fibrils with an average width of 4.3 ± 0.7 nm (Figure 4C), as we have previously reported.^40^ Fibers of this dimension have β-sheets that are approximately one sheet in width, in contrast to the **L1** pleated β-sheet nanoribbons and nanotubes that have two β-sheets aligned side-by-side. Interestingly, the **L2/D2** assemblies were more variable in appearance, with a broader range and average width of 5.8 ± 1.3 nm (Figure 4D). While the **L2** pleated β-sheets were much more uniform in width along the entire length of each fibril, the **L2/D2** rippled β-sheets were more obviously polymorphic along the length of each fibril. This variability of width along the fibril suggests a greater degree of disorder within the peptide packing structure. Regardless, the assemblies of **L2/D2** are structurally distinct from **L2** pleated β-sheet fibrils, providing additional evidence for coassembly into the enantiomers into rippled β-sheets.

Again, as previously reported, the **L3** peptide self-assembled into pleated β-sheets with a unique nanotape morphology. The **L3** nanotapes were flat and irregularly twisted nanotapes with large average widths of 29.6 ± 9.5 nm (Figure 4E). While FTIR analysis at 1 mM showed predominantly random structure with minor contributions from β-sheet structure, TEM analysis revealed the presence of these assembled nanotapes. In contrast, the **L3/D3** mixtures displayed abundant fibrils that were strikingly different in morphology from the pleated β-sheet **L3** nanotapes. The putative **L3/D3** rippled β-sheet assemblies were much thinner, nontwisted fibrils with an average width of 4.4 ± 0.8 nm (Figure 4F). This width is consistent with a one-peptide-wide packing arrangement. The dramatic difference in morphology between assemblies observed in **L3** versus **L3/D3** solutions is strong evidence for the formation of rippled β-sheets in the enantiomeric mixtures.

**L4** solutions at 1 mM were primarily random structures by FTIR analysis. TEM images of these solutions showed spherical micellar aggregates and twisted fibers with an average width of 27.1 ± 9.5 nm (Figure 4G). Occasionally, nanotapes like those seen in **L3** solutions were observed emerging from these spherical aggregates. We hypothesize that **L4** self-assembly may initiate with hydrophobic collapse into the spherical, random-structured aggregates, within which pleated β-sheet self-assembly may occur slowly. Distinct from **L4** solutions, TEM images of racemic **L4/D4** mixtures at 1 mM revealed well-defined fibers with an average width of 15. 5 ± 5.1 nm—less than half the width of the self-assembled **L4** nanotapes (Figure 4H). No visible twisting was observed in these **L4/D4** fibrils, and no spherical aggregates were observed in the racemic solutions. Again, the distinctive morphology of the assemblies formed from the **L4/D4** mixtures compared to the **L4** solutions is further evidence of rippled β-sheet formation from the mixed enantiomers.

Finally, **L5** solutions exhibited an overall lack of well-defined assembled structures (Figure 4I), consistent with the FTIR results, which showed random structures at all concentrations. TEM imaging revealed sparse amorphous aggregate, reinforcing the limited self-assembly capacity of **L5**. However, mixtures of **L5/D5** resulted in the formation of fiber structures interspersed with micellar aggregates (Figure 4J). These assemblies displayed some degree of polymorphism, which could be attributed to an incomplete assembly. Overall, this TEM analysis corroborated the FTIR findings, reinforcing the consistent observance of putative rippled β-sheets from racemic mixtures of enantiomers under conditions when pleated β-sheets of single enantiomers were less likely to be observed. In addition, the observed rippled β-sheets had distinctive morphologies relative to the self-assembled pleated β-sheet counterparts. These results further support the formation of rippled β-sheets in these peptides and their increased propensity to form ordered structures compared to corresponding pleated β-sheet self-assembly.

To gain further insight into the morphology of assemblies for peptide systems with reduced β-sheet propensity, the **L4**, **L4/D4**, **L5**, and **L5/D5** systems were also assembled at a concentration of 4 mM for TEM analysis. **L4** fibers at 4 mM were notably thinner than those observed at 1 mM, with an average width of 9.1 ± 1.7 nm—nearly a 4-fold decrease in width (Figure 5A). Another distinct difference at this higher concentration was the increased presence of micelle-like aggregates along the **L4** fibers. The narrower width observed in **L4** fibers upon increasing the concentration is not well understood. However, the large increase in observed micellar, spherical aggregates agrees with the **L4** FTIR spectrum at 4 mM, which showed mostly random structure aggregates. A more pronounced change was observed in the **L4/D4** rippled β-sheet coassembly at 4 mM (Figure 5B). Unlike the uniform fibers seen at 1 mM, these assemblies exhibited polymorphic structures with varied widths. The average width of the fibers was 37.7 ± 17.1 nm, but a broad distribution was noted, with widths ranging from 18 to 108 nm (Figure 5B). This high variability suggests that increasing concentration promotes structural heterogeneity within the **L4/D4** system, possibly due to the increased rate of formation at higher concentration. **L5** solutions showed minimal fibril formation at 1 mM but exhibited more abundant ordered structures in TEM images at 4 mM. Fiber-like structures were observed, though their widths varied considerably, averaging 22.4 ± 5.8 nm (Figure 5C). While **L5** does not readily self-assemble into pleated β-sheet fibrils at low concentrations, a greater degree of structural organization was observed at these higher concentrations, even though FTIR spectra indicated a significant amount of random structures. Lastly, **L5/D5** mixtures at 4 mM exhibited more abundant fiber structures compared to **L5** alone (4 mM), though they remained irregular in morphology. These **L5/D5** fibers had an average width of 27.5 ± 7.7 nm, with considerable variation across the sample (Figure 5D). These TEM images are again consistent with morphologically distinct fibers supporting the preferential assembly of racemic mixtures into rippled β-sheets compared to self-assembly into single-component pleated β-sheets at the same concentrations.

**Figure 5:**
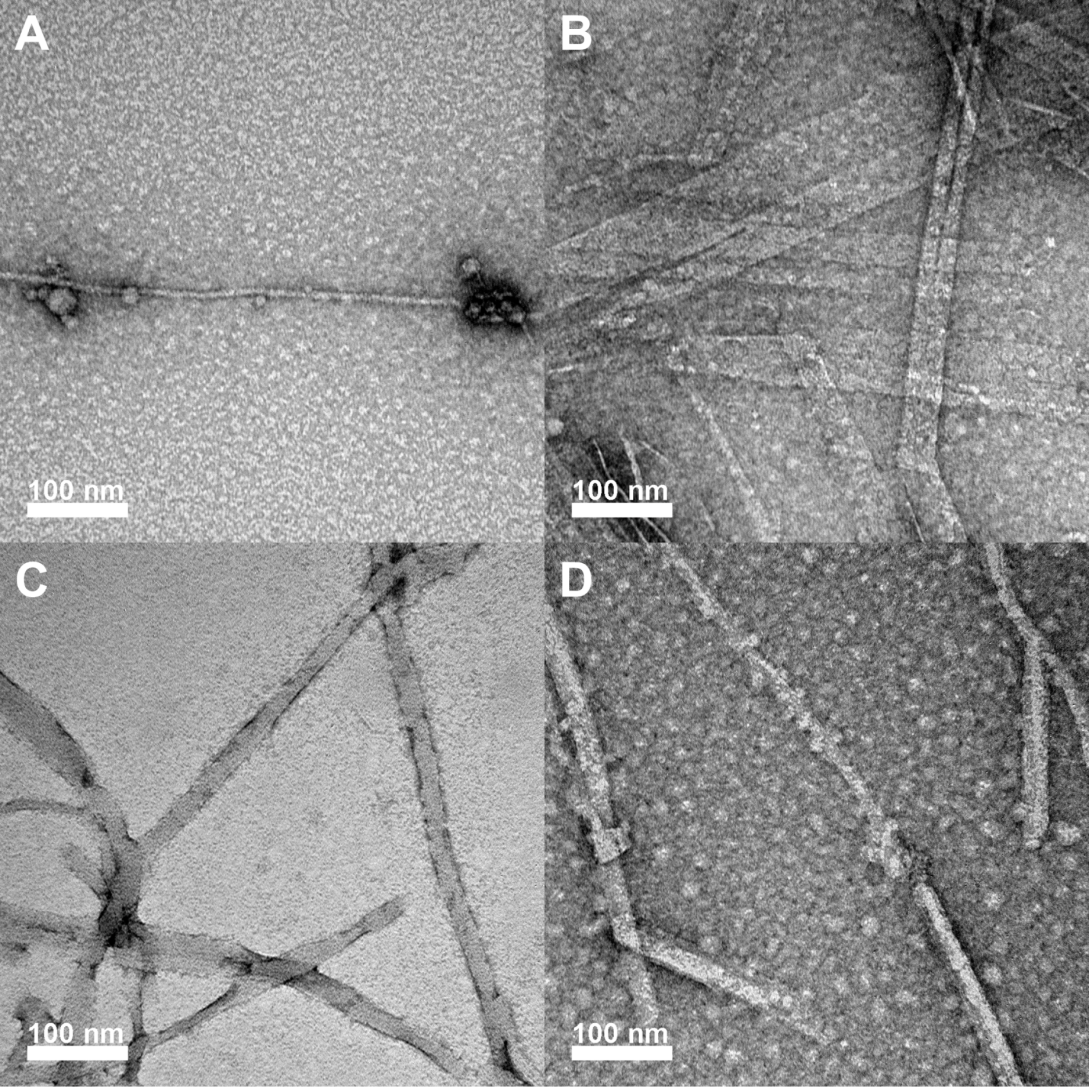
Negative stain TEM images of **(A) L4**, **(B) L4/D4**, **(C) L5**, **(D) L5/D5** solutions at 4 mM concentrations. All samples were assembled at a total peptide concentration of 4 mM, with the racemic mixtures assembled at 2 mM concentrations of each enantiomer. **L4**, **L4/D4**, **L5**, and **L5/D5** solutions were diluted to 1 mM before deposition on TEM grids.

### Isotope-Edited Fourier Transform Infrared Spectroscopy to Support l/d-Packing Arrangements in Putative Rippled β-Sheets

FTIR analysis provided evidence that enantiomeric mixtures of these variants have an enhanced propensity to assemble into putative rippled β-sheets compared to the self-assembly of the enantiopure counterparts into pleated β-sheets. Likewise, TEM analysis provides additional evidence for the formation of rippled β-sheets based on the morphological differences observed in the nanostructures of enantiomeric mixtures compared to the self-assembled complements. We next sought additional verification for rippled β-sheet assembly from enantiomeric mixtures by attempting to confirm the predicted alternating l/d packing structure in the racemic mixtures.

To validate the structural l/d arrangement of enantiomeric peptides within the expected rippled β-sheets, we employed an isotope-edited FTIR (IE-IR) analysis. This technique utilizes site-specific isotopic labeling and the specific through-space coupling interactions between labeled atoms to provide structural insights. Peptides were synthesized with strategically placed ^13^C stable isotopes at carbonyl carbons of selected amino acids. The 1-^13^C carbonyl isotope introduces a shifted amide I FTIR peak to a lower wavenumber due to the vibrational shift of the heavier ^13^C isotope. In a hypothetical antiparallel pleated β-sheet system, a ^13^C-label placed near one end of a peptide would result in cross-strand labels within the β-sheet residing on opposite ends of the β-sheet (Figure 6A). In the case of the same hypothetical peptide, if two ^13^C labels are introduced on either end of the peptide, the resulting ^13^C labels within an assembled pleated β-sheet will be in close proximity, leading to isotopic coupling, which will shift the labeled peaks to an even lower frequency (Figure 6B). It is possible to use strategic ^13^C-labeling and the resulting coupling effects to estimate β-sheet packing arrangements in pleated β-sheet systems.^18,24,68–70^ Using this approach, it is also possible to differentiate between self-sorting into pleated β-sheets or coassembling into rippled β-sheets in mixtures of enantiomeric peptides. This differentiation can be achieved by comparing the coupling effects in appropriately labeled pleated β-sheet systems (Figure 6B) with an analogous mixture of enantiomers in which the l-peptide replicates the same ^13^C-labeling pattern, and the d-peptide is unlabeled (Figure 6C). If the enantiomers are coassembled into rippled β-sheets with alternating l/d peptides, any coupling relationship between ^13^C-labels will be interrupted by the unlabeled d-peptide, and the resulting FTIR spectrum should exhibit amide I peaks that are consistent with an uncoupled system. We have previously used this strategy to confirm rippled β-sheet formation in **L1/D1** mixtures and in racemic mixtures of l- and d-amyloid β fragments.^18,24^ We applied this strategy to gather additional evidence for the formation of rippled β-sheets for the **L1/D1**, **L2/D2**, and **L3/D3** systems. Because the self-assembly of the **L4** and **L5** peptides was highly inefficient, this strategy could not be used for analysis of rippled β-sheet formation in the corresponding **L4/D4** and **L5/D5** systems, since it was not possible to obtain IE-IR spectra for the pleated β-sheets of **L4** and **L5**, which is a necessary control to establish the appearance of coupling between chosen labels.

**Figure 6:**
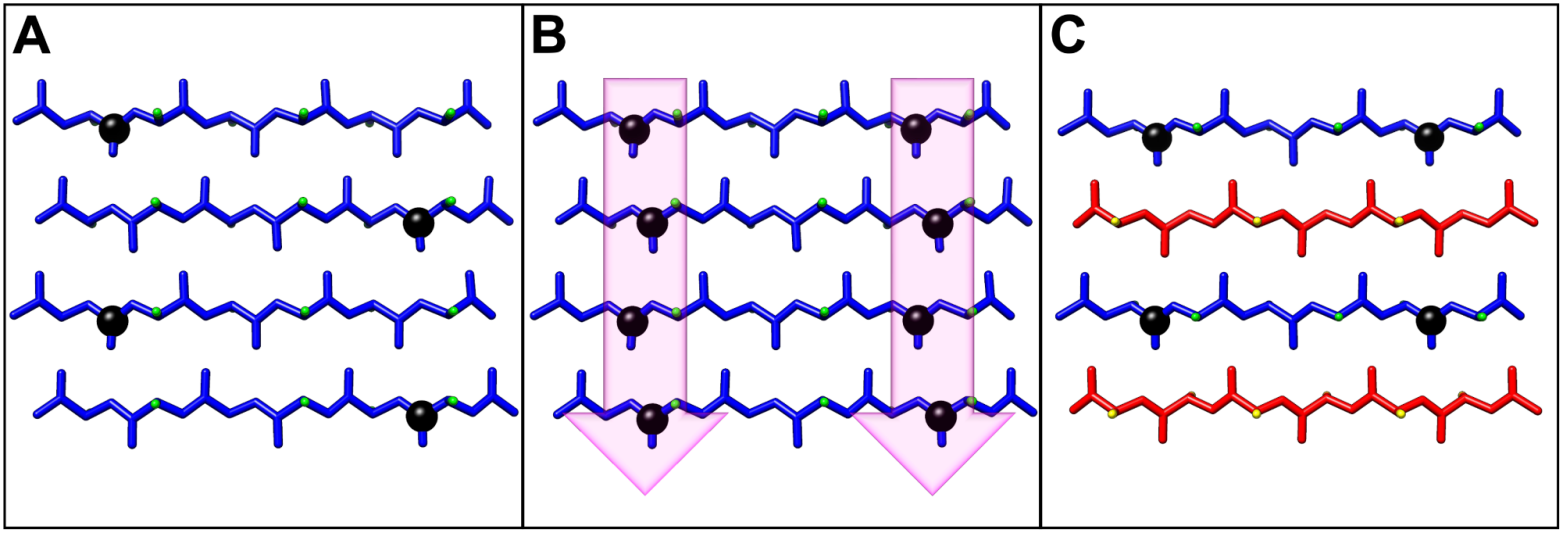
Schematic representation of ^13^C-isotopically labeled peptides to interrogate packing structure in β-sheets. (A) Antiparallel pleated β-sheet with a stable isotope 1-^13^C carbonyl-labeled peptide at a single amino acid. The ^13^C label is represented as a black sphere. In this antiparallel orientation, ^13^C-labels in neighboring β-strands are not in close proximity and will not be engaged in a coupling relationship. (B) Antiparallel pleated β-sheet with stable isotope 1-^13^C carbonyl-labels (black spheres) included at two amino acids. With two labels in the selected positions, the ^13^C-labels in neighboring β-strands are now in close proximity, facilitating a coupling relationship between the two labels. The carbonyl peaks in this coupled relationship will exhibit a pronounced shift in the amide I region of the FTIR spectrum. (C) An antiparallel rippled β-sheet with a ^13^C-double-labeled l-strand (blue, ^13^C-labels represented as black spheres) and an unlabeled d-strand (red). In this rippled β-sheet configuration, the unlabeled d-peptides interfere with the coupling of the ^13^C-labeled in the l-enantiomers. If this l/d pattern exists, the FTIR spectrum of these paired peptides in the rippled β-sheet would be expected to show a shift consistent with an uncoupled relationship.

For the **L1** and **L2** systems, several ^13^C-labels were used to enable IE-IR analysis. Single-labeled **L1** and **L2** peptides were synthesized with 1-^13^C labels at the Phe-1 position (**L1X**, Ac-FKFEFKFE-NH_2_; **L2X**, Ac-FKFKFEFE-NH_2_; underlined Phe residues are 1-^13^C-labeled, see Tables S1, S7, and S9 and Figures S11–S16 and Figures S27–S32 in Supporting Information for peptide purification and characterization data). The single-labeled peptides were used to establish the appearance of pleated β-sheet FTIR spectra in which the labeled residues are uncoupled in antiparallel pleated β-sheet arrangements but may be coupled in the case of parallel pleated β-sheets. This is significant, since cryo-EM structural analysis of **L1** pleated β-sheet bilayer nanoribbons and nanotubes has established that both arrangements are present, with the exterior β-sheets having an antiparallel arrangement and the interior β-sheets having a parallel orientation.^20^ Double-labeled **L1** and **L2** peptides were also synthesized with 1-^13^C labels at the Phe-1 and Phe-7 positions (**L1XX**, Ac-FKFEFKFE-NH_2_; **L2XX**, Ac-FKFKFEFE-NH_2_; underlined Phe residues are 1-^13^C-labeled). With labels in these two positions, all ^13^C atoms are in a coupled orientation in the context of both antiparallel and parallel pleated β-sheets. FTIR spectra were obtained from several mixtures of these peptides: **L1X** and **L2X**, **L1XX** and **L2XX**, and **L1XX/D1** and **L2XX/D2** (Figure 7A and B). These various spectra were compared to the FTIR spectra of unlabeled **L1**, **L2**, **L1/L2**, and **L2/D2** to determine whether the racemic mixtures displayed coupling patterns consistent with coassembled rippled β-sheets or self-sorted pleated β-sheets.

**Figure 7:**
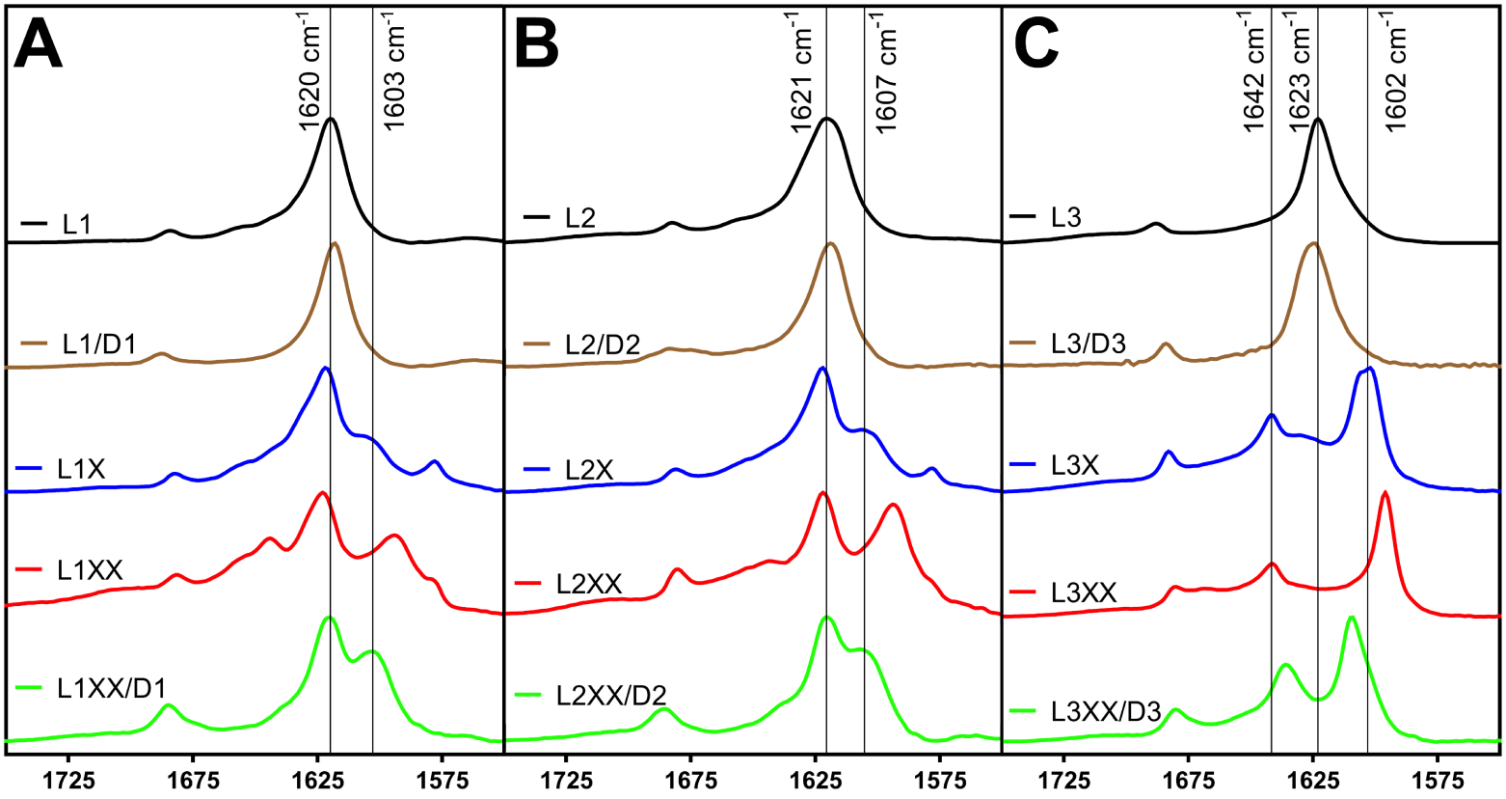
Isotope-edited Fourier transform infrared spectra for confirmation of pleated and rippled β-sheet arrangements for **L1**, **L2**, **L3**, **L1/D1**, **L2/D2**, and **L3/D3** solutions. **(A) L1** and **L1/D1** solutions (1 mM). Unlabeled **L1** is shown in black (reproduced from Figure 2), unlabeled **L1/D1** is shown in brown (reproduced from Figure 3), **L1X** is shown in blue, **L1XX** is shown in red, and **L1XX/D1** is shown in green. **(B) L2** and **L2/D2** solutions (1 mM). Unlabeled **L2** is shown in black (reproduced from Figure 2), unlabeled **L2/D2** is shown in brown (reproduced from Figure 3), **L2X** is shown in blue, **L2XX** is shown in red, and **L2XX/D2** is shown in green. **(C) L3** and **L3/D3** solutions (4 mM). Unlabeled **L3** is shown in black (reproduced from Figure 2), unlabeled **L3/D3** is shown in brown (reproduced from Figure 3), **L3X** is shown in blue, **L3XX** is shown in red, and **L3XX/D3** is shown in green.

**L1** pleated and **L1/D1** rippled β-sheets were analyzed using unlabeled **L1** and **L1/D1** solutions compared with labeled **L1X**, **L1XX**, and **L1XX/D1** solutions. These spectra were obtained at total peptide concentrations of 1 mM. As described previously (Figure 2A), **L1** and **L1/D1** assemblies had nearly identical FTIR spectra, with a strong amide I peak at 1618 cm^–1^. These spectra are reproduced in Figure 7A for convenience. We then compared the unlabeled **L1** spectrum to that of single-labeled **L1X** peptide, which displayed a typical β-sheet peak at 1622 cm^–1^ and a lower frequency peak at 1600 cm^–1^, corresponding to the^13^C isotope label at Phe-1 (Figure 7A). This establishes the manifestation of a pleated β-sheet spectrum in which the carbonyl labels are not highly coupled. The double-labeled **L1XX** assembly exhibited more pronounced peak separation, with amide I stretches at 1622 cm^–1^ and 1591 cm^–1^. The shifting of the isotope-labeled peak from 1600 cm^–1^ to 1591 cm^–1^ is evidence of strong coupling between the ^13^C-carbonyl labels at Phe-1 and Phe-7. Finally, in the **L1XX/D1** spectrum the isotope peak appears at 1602 cm^–1^, consistent with decoupling of the Phe-1 and Phe-7 ^13^C-labels. This indicates that the unlabeled **D1** and the labeled **L1XX** peptides are arranged in an alternating fashion supporting rippled β-sheet formation in this system. These results are consistent with our previously reported work investigating rippled β-sheet formation from **L1/D1** mixtures.^18^

Next, **L2** pleated and **L2/D2** rippled β-sheets were likewise analyzed using unlabeled **L2** and **L2/D2** solutions compared with labeled **L2X**, **L2XX**, and **L2XX/D1** solutions. These spectra were also obtained at total peptide concentrations of 1 mM. These **L2** analyses gave similar results to those observed with **L1** and **L1/D1** assemblies. Unlabeled **L2** and **L2/D2** assemblies produced FTIR spectra with amide I shifts at 1618 cm^–1^ (Figure 2A and reproduced in Figure 7B). The FTIR spectrum for Phe-1-labeled **L2X** exhibited peaks at 1622 cm^–1^ and 1604 cm^–1^, while the double-labeled **L2XX** spectrum showed both the 1622 cm^–1^ absorbance and a larger magnitude shifted absorbance for the heavy Phe-1 and Phe-7 ^13^C-labels at 1593 cm^–1^, indicative of coupling (Figure 7B). When **L2XX** is mixed with the unlabeled **D2** enantiomer, the isotope peak returned to 1604 cm^–1^, consistent with an alternating orientation between the **L2XX** and **D2** peptides in a rippled β-sheet configuration.

Finally, the **L3** and **L3/D3** system was interrogated by comparing the spectra of **L3** and **L3/D3** with those of **L3X**, **L3XX**, and **L3XX/D3** peptide solutions. The **L3** and **D3** sequences differ significantly from the **L1**, **L2**, **D1**, and **D2** peptides, thus requiring a unique pattern for ^13^C label incorporation. The **L3X** sequence has a single 1-^13^C label at Phe-3 (Ac-KEFFFFKE-NH_2_, with the underlined Phe residue having a ^13^C atom at the carbonyl carbon). The **L3XX** sequence has two 1-^13^C labels at Phe-3 and Phe-6 (Ac-KEFFFFKE-NH_2_, with the underlined Phe residues having ^13^C atoms at the carbonyl carbons). The **L3** and **L3/D3** IE-IR experiments were conducted at 4 mM peptide concentrations to ensure more complete β-sheet assembly and to avoid complications in the spectra from random structured peptides that were observed at lower concentrations of **L3** (Figure 2A and B).

The similar packing architecture and labeling patterns of the **L1** and **L2** and the corresponding **L1/D1** and **L2/D2** systems provided comparable FTIR spectral characteristics, whereas the differing morphological packing and ^13^C-label positions of the **L3** and **L3/D3** systems gave rise to unique spectral signatures. The unlabeled spectra for **L3** and **L3/D3** showing characteristic β-sheet signatures at 1623 cm^–1^ (Figures 2B and 3B) are reproduced in Figure 7C. The FTIR spectrum of the single-labeled **L3X** had absorbances at 1642 cm^–1^ and 1602 cm^–1^, in addition to the parent peak at 1623 cm^–1^ (Figure 7C). The peak at 1602 cm^–1^ appears to be the product of two closely spaced peaks. The spectrum of **L3XX** was similar to that of **L3X**, although it showed a slightly more pronounced peak separation at 1641 cm^–1^ and 1596 cm^–1^ and the parent peak at 1623 cm^–1^ was no longer strongly visible. In addition, the 1595 cm^–1^ absorbance appeared to be a single signal without the close doubling observed in the **L3X** spectrum. These coupling patterns were distinct from those observed in the **L1** and **L2** pleated β-sheet spectra due to a distinct packing arrangement in **L3** assemblies, as evidenced by the unique **L3** nanotape morphology. The **L3XX/D3** mixture gave a unique FTIR spectra, with major peaks at 1636 cm^–1^ and 1609 cm^–1^, strongly shifted from those observed in **L3X** or **L3XX** spectra. While the exact packing architecture of the L3 pleated β-sheets or the L3/D3 rippled β-sheets cannot be extrapolated from these spectroscopic analyses, they provide strong corroborating evidence for the formation of multicomponent assemblies consistent with rippled β-sheets for the racemic mixtures.

The **L4**, **L5**, **L4/D4**, and **L5/D5** systems are not candidates for IE-IR analysis to validate rippled β-sheet formation from the mixed enantiomers. The central complication with these systems is that **L4** and **L5** have a low self-assembly propensity, with FTIR spectra showing significant random structures and only minor contributions from β-sheet structures (Figures 2 and 3). Even though the enantiomeric mixtures, **L4/D4** and **L5/D5**, have significantly higher β-sheet content, IE-IR analysis can only be effectively conducted in systems where the single enantiomer spectra are dominantly β-sheet. Since the spectra for **L4** and **L5** are primarily random structure, it is impossible to establish strong coupling interactions from these solutions. Without the presence of coupling relationships in the pleated β-sheet systems, it is impossible to achieve meaningful comparisons for the multicomponent **L4/D4** and **L5/D5** solutions.

Nonetheless, the IE-IR experiments have provided strong additional evidence for the formation of rippled β-sheets in mixed enantiomer solutions. The **L1/D1** and **L2/D2** systems show clear evidence of interrupted coupling when the unlabeled d-enantiomers are mixed with l-stereoisomers that have coupled ^13^C-labels in the corresponding pleated β-sheets. Likewise, IE-IR analysis of the **L3/D3** mixture also shows clear evidence of altered coupling when labeled l-peptide is mixed with unlabeled d-peptide. By extrapolation, we expect on the basis of increased β-sheet structure in racemic mixtures and altered assembly morphology, that the **L4/D4** and **L5/D5** systems are also forming rippled β-sheets even though IE-IR studies were not possible.

### Thermodynamic Comparison of Pleated β-Sheet Self-Assembly and Rippled β-Sheet Coassembly of Enantiomeric Mixtures

Based on the qualitative observation that racemic mixtures have higher β-sheet content than corresponding solutions of enantiopure peptides, we conducted a thermodynamic comparison of pleated β-sheet assembly versus rippled β-sheet enantiomer coassembly. Self-assembling peptides have characteristic critical aggregation concentrations (C_r_) that are reflective of the peptide sequence and the self-assembly conditions (solvent, pH, temperature, etc.).^24,42,66,71–73^ The C_r_ represents the minimum peptide concentration required for self-assembly to occur. At this concentration, it is assumed that the peptide assembly is in equilibrium with monomer. This equilibrium state can be represented by the expression: fibril_n_ + monomer ⇌ fibril_n+1_(eq 2).^42^ Based on this expression, the equilibrium association constant, K_a_, can be expressed as K_a_ = [fibril_n_+1]/[fibril_n_]­[monomer] (eq 3). The equilibrium association constant is the reciprocal of the critical concentration (K_a_ = 1/C_r_) (eq 4). Measurement of C_r_ values for related peptide systems can be used to calculate differences in Gibbs free energy (ΔΔG) of assembly, facilitating a thermodynamic comparison between pleated β-sheet assembly of enantiopure peptides and rippled β-sheet assembly of the corresponding racemic mixture of enantiomeric peptides.

We determined experimental critical concentration (C_r_) values for each peptide assembly system using an ultracentrifugation sedimentation analysis.^42^ These assays are conducted by allowing the peptides to self-assemble and then subjecting them to ultracentrifugation to separate aggregate structures from monomeric peptides. The monomer concentration in the supernatant is measured by injection of aliquots of predetermined volume onto an analytical HPLC column and correlation of the peak area to an HPLC concentration curve that is calibrated by amino acid analysis. The determined monomer concentration is the critical concentration (C_r_) for the peptide under the assembly conditions. For these studies, peptides were assembled at 1 mM (**L1**, **L1/D1**, **L2**, **L2/D2**, and **L3/D3**) or 4 mM (**L3**, **L4**, **L4/D4**, **L5**, **L5/D5**) concentrations based on the β-sheet content as determined from FTIR experiments. Peptides were allowed to assemble for 24 h and were then subjected to ultracentrifugation to separate aggregates from monomeric (unassembled) peptide.^42^ The concentration of monomeric peptides in the supernatant was quantified via analytical HPLC. C_r_ values for self-assembled pleated β-sheet (**L1**–**L5**) and racemic rippled β-sheet (**L1/D1**–**L5/D5**) assemblies are summarized in Figure 8. The C_r_ values along with the calculated ΔG and comparative ΔΔG values for corresponding pleated and rippled β-sheet systems are also presented in Table 3.

**Figure 8:**
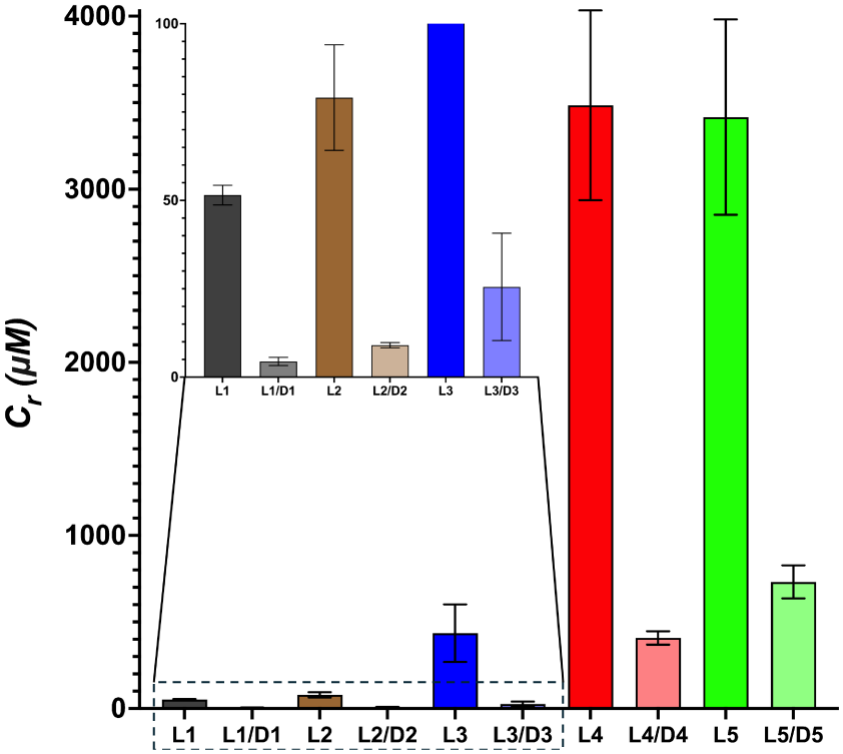
Experimentally determined critical concentration values (C_r_) for each pleated β-sheet (**L1**–**L5**) and rippled β-sheet (**L1/D1**–**L5/D5**) system.

**Table 3:** Thermodynamic Comparison of Pleated β-Sheet (**L1**–**L5**) and Rippled β-Sheet (**L1/D1**–**L5/D5**) Assembly^a^

Peptide Assembly	Critical Concentration (C_r_) (μM)	ΔG (kcal mol^–1^)	ΔΔG (Rippled β-Sheet to Pleated β-Sheet) (kcal mol^–1^)
**L1**	52 ± 3	–5.8 ± 0.03	–
L1/D1	4 ± 1	–7.4 ± 0.1	ΔΔG_L1/D1–L1_ = −1.6
**L2**	79 ± 14	–5.6 ± 0.1	–
L2/D2	9 ± 0.7	–6.9 ± 0.04	ΔΔG_L2/D2-L2_ = −1.3
**L3**	435 ± 157	–4.6 ± 0.2	–
L3/D3	26 ± 14	–6.3 ± 0.4	ΔΔG_L3/D3-L3_ = −1.7
**L4**	3485 ± 517	–3.4 ± 0.1	–
L4/D4	408 ± 39	–4.6 ± 0.1	ΔΔG_L4/D4–L4_ = −1.2
**L5**	3417 ± 532	–3.4 ± 0.1	–
L5/D5	732 ± 90	–4.3 ± 0.1	ΔΔG_L5/D5–L5_ = −0.9

a Critical concentration values (C_r_) in each peptide assembly condition are reported as the average value of at least 3 experiments with error reported as the standard deviation. ΔG values for each assembly condition were determined by converting the C_r_ value to the equilibrium association constant, K_a_, using the expression K_a_ = 1/C_r_. ΔG was obtained using the equation ΔG = −RTlnK_a_. ΔΔG values were then obtained by the difference between the ΔG values for corresponding pleated and rippled β-sheet systems.

These sedimentation analysis experiments clearly illustrate the strong thermodynamic preference for coassembly of racemic mixtures of enantiomers into rippled β-sheets compared to self-assembly of pure enantiomers into pleated β-sheets. The **L1** peptide had a C_r_ of 52 μM, and the **L1/D1** mixture displayed an even lower C_r_ of 4 μM, indicating a thermodynamic preference of 1.6 kcal mol^–1^ for formation of rippled β-sheets over pleated β-sheets. We have previously reported an enthalpic preference of 9.3 kcal mol^–1^ for rippled β-sheet over pleated β-sheet assembly in an **L1/D1**-related system based on isothermal titration calorimetry experiments. The sedimentation analysis herein confirms this preference. **L2** had similarly low C_r_ of 79 μM, with the **L2/D2** coassembly yielding a lower C_r_ of 9 μM, corresponding to a 1.3 kcal mol^–1^ preference for rippled β-sheet formation. As the peptide sequences deviated from an alternating hydrophobic/hydrophilic sequence pattern, the observed C_r_ values increased substantially. The C_r_ value for **L3** was 435 μM, nearly an 8-fold increase compared to **L1**, highlighting the critical role of sequence patterning in pleated β-sheet self-assembly propensity. The **L3/D3** coassembly had a significantly reduced C_r_ value of 26 μM. This corresponds to an astonishing 1.7 kcal mol^–1^ advantage for rippled β-sheet formation by **L3/D3** compared to pleated β-sheet formation by **L3**. The C_r_ for coassembled **L3/D3** is nearly half the C_r_ of **L1** pleated β-sheets, which assembles with great efficiency. **L4** and **L5** pleated β-sheet self-assembly had much higher comparative C_r_ values of 3485 μM and 3417 μM, respectively. These higher values are consistent with FTIR spectroscopy experiments that showed a relatively low self-assembly propensity for these sequences. However, when these peptides are mixed with their enantiomers at identical total peptide concentrations, both **L4/D4** and **L5/D5** exhibited a marked reduction in C_r_ values (408 μM and 732 μM, respectively). These C_r_ values correspond to thermodynamic preferences of 1.2 kcal mol^–1^ for **L4/D4** rippled β-sheet formation over **L4** pleated β-sheet formation and 0.9 kcal mol^–1^ for **L5/D5** rippled β-sheet formation over **L5** pleated β-sheet formation. These experiments strongly illustrate the enhanced assembly properties observed for enantiomeric mixtures compared to enantiopure solutions of the same peptides.

### All-Atom Molecular Dynamics Simulations of Pleated β-Sheet and Rippled β-Sheet Assembly

We subsequently compared the relative heterochiral coassembly of racemic mixtures of the l- and d-peptide sequences using unbiased all-atom molecular dynamics simulations. Every pair of enantiomeric sequences was studied using up to four independent simulations performed at a peptide concentration of 16 mM. Figure 9A shows the time dependence of the number $NHB_{inter}^{b-b}$ of interbackbone hydrogen bonds per peptide in the simulations. Initially, this quantity is close to zero in all simulations $(NHB_{inter}^{b-b}∼0$ ) as peptides are dispersed in the solution. For mixtures **L1/D1**, **L2/D2**, and **L3/D3**, $NHB_{inter}^{b-b}$ increases and saturates when at least 3 hydrogen bonds are formed on average per peptide. This takes place in less than 1 μs in three out of 4 trials for **L1/D1** sequences, at approximately 1 μs in two trials using **L2/D2** peptides, and in more than 1 μs for **L3/D3** mixtures. Simulations with **L4/D4** and **L5/D5** do not show a significant increase in $NHB_{inter}^{b-b}$ within 3 μs. This highlights a rate of aggregation that decreases in the following order: **L1/D1** > **L2/D2** > **L3/D3** > **L4/D4** ≈ **L5/D5**. The number of residues adopting a β-conformation per peptide is shown in Figure 9B. This quantity correlates strongly with $NHB_{inter}^{b-b}$ increasing and saturating within the same timeframes for sequences **L1/D1**, **L2/D2**, and **L3/D3**. Saturation takes place when four or more residues adopt a β-conformation. For sequences **L4/D4** and **L5/D5**, residues do not form a significant number of β-conformations at the end of 3 μs, although there is continuous increase in β-conformations after 2 μs for **L4/D4**. This suggests that equimolar mixtures of **L4** and **D4** peptides may form β-assemblies if simulations were extended beyond 3 μs, highlighting the importance of time in nonequilibrium systems.

**Figure 9:**
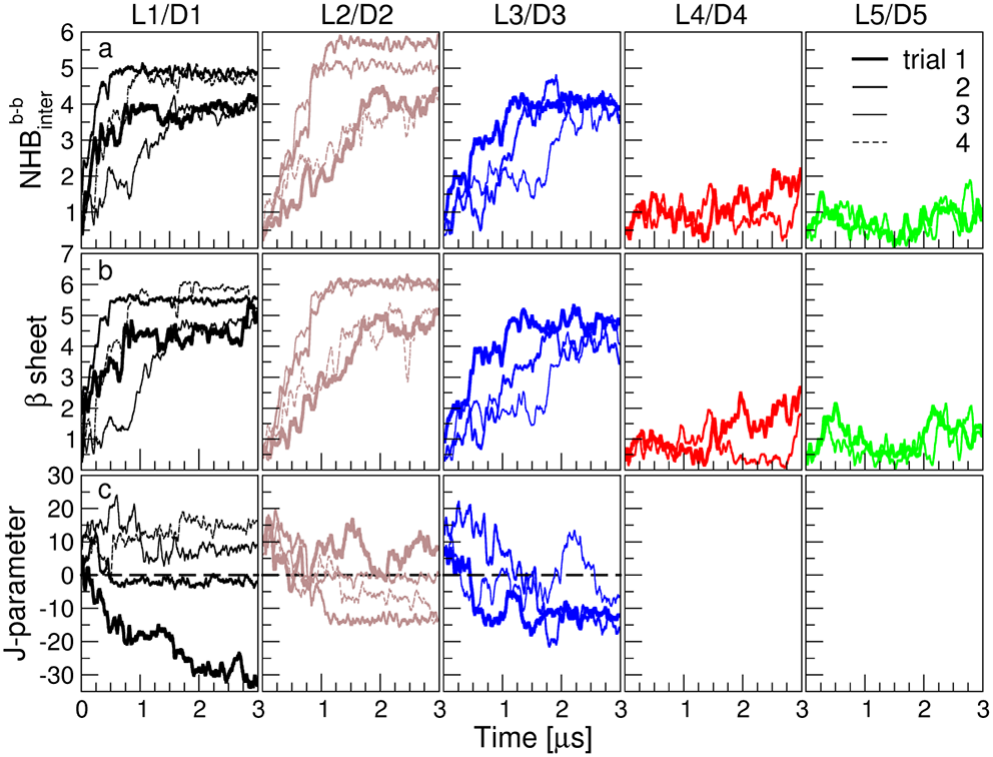
Long unbiased molecular dynamics simulations of equimolar mixtures of l- and d-peptides. Columns from left to right correspond to sequences **L1/D1**, **L2/D2**, **L3/D3**, **L4/D4**, and **L5/D5**. (A) Time evolution of the number of interbackbone hydrogen bonds per peptide. (B) Number of residues adopting a β-conformation per peptide as a function of time. (C) J-parameter, which quantifies the relative frequency of interactions between peptides of identical (l/l and d/d) versus opposite (l/d) stereochemistry. Independent simulations are distinguished by line thickness.

In Figure 9C, we quantify the position of l- relative to d-peptides in β-sheets by computing the J-parameter. The latter is defined as

5 $$\mathrm{J-parameter}=\left[NHB_{inter}^{b-b}\left(L-L\right)+NHB_{inter}^{b-b}\left(D-D\right)\right]-NHB_{inter}^{b-b}\left(L-D\right)$$

where the two terms inside the bracket and the last term of the equation correspond to the numbers of hydrogen bonds between peptides of the same (l–l and d–d) and opposite (l–d) chirality, respectively. Accordingly, this quantity distinguishes between aggregates in which l- and d-peptides are added to β-sheets randomly (J-parameter = 0), in an alternating fashion (negative J-parameter), or are partitioned to distinct β-sheets (positive J-parameter). However, for the small number of peptides in our simulations, the J-parameter from independent simulations exhibit a large dispersion. In the Supporting Information (Figure S88), we show that in simulations containing a total of 10 peptides, where l- peptides interact without any preference for l- and d-peptides in solution, the mean J-parameter is close to zero as expected, with a standard deviation of σ ∼ 15. In Figure 9C, the J-parameter ranges from −30 to +15, which, due to the small number of simulations carried out, does not allow us to exclude the possibility that the aggregates are formed randomly. However, the J-parameter in one of the **L1/D1** simulations is −30 (more than 2σ from the mean), it is −10 and −15 (∼1σ from the mean) in two of the **L2/D2** simulations, and it is negative (∼1σ from the mean) in all three **L3/D3** simulations. This bias for negative values suggests a preferential interaction of l-peptides with d-peptides, and vice versa. The J-parameter was not computed for **L4/D4** and **L5/D5** systems as peptides in these simulations do not form a significant number of interbackbone hydrogen bonds.

For each peptide sequence investigated, Figure 10 shows the final structure from the racemic simulation with the lowest J-parameter (calculated using eq 5). For **L1/D1**, **L2/D2**, and **L3/D3** systems, these structures are characterized by the formation of cross-β assemblies. In **L1/D1** and **L2/D2** systems, phenylalanine and charged residues alternate along the peptide sequence, giving rise to β-sheets with one face enriched in nonpolar residues and the other in charged residues. This leads to packing of nonpolar β-sheet faces against each other to minimize the exposure of hydrophobic phenylalanine side chains to water, resulting in a single well-defined hydrophobic core (Figure 10A–B). In contrast, the presence of four consecutive phenylalanine residues in the **L3/D3** sequence leads to phenylalanine exposure on both faces of the β-sheet. This loss of strict amphipathic segregation yields a cross-β assembly in which hydrophobic contacts are distributed across both interfaces rather than confined to a single, well-defined stacking surface (Figure 10C). In the **L4/D4** and **L5/D5** systems, peptides form a loosely packed disordered cluster (Figure 10D–E).

**Figure 10:**
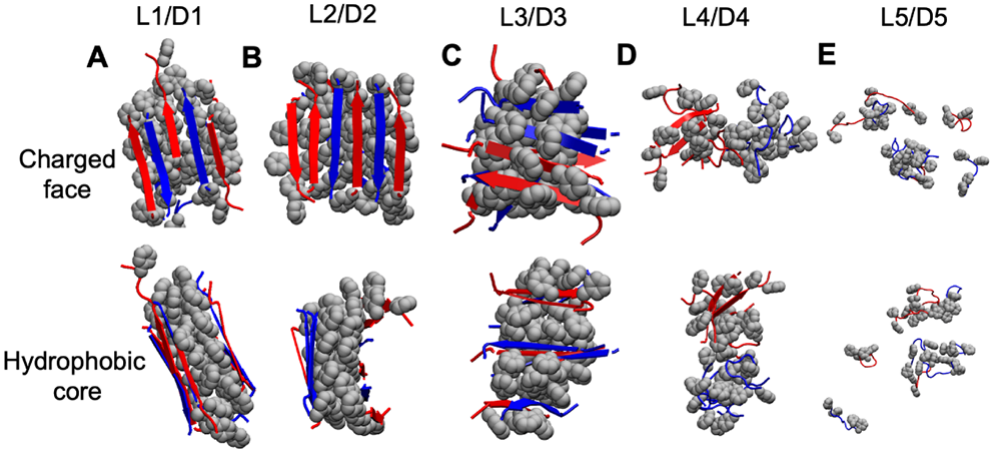
Structures of the aggregates formed at the end of simulations performed using (A) **L1/D1**, (B) **L2/D2**, (C) **L3/D3**, (D) **L4/D4**, and (E) **L5/D5** peptides. (Top) View of the charged face of one of the β-sheets and (bottom) the hydrophobic core of the different assemblies. Blue and red colors are used to represent l- and d-peptides, and phenylalanine side chains are shown in gray.

Figure 10 shows the backbone of l- and d-peptides in red and blue, respectively. This provides visual insights into the alternation of mirror image peptides in β-sheets, which was quantified by the J-parameter in Figure 9C using eq 5. The charged faces of the **L1/D1**, **L2/D2**, and **L3/D3** systems that are depicted in Figure 10 contain 4, 5, and 3 β-sheet dimers, respectively. All four dimers from the **L1/D1** structure are made by alternating between l- and d-peptides. In the **L2/D2** system, only one out of 5 β-sheet dimers is made by peptides with the same chirality, i.e., **D2/D2**. The **L3/D3** system shows a more diverse composition with one and two β-sheet dimers being made from peptides of opposite and identical stereochemical composition (**L3/L3** and **D3/D3**), respectively.

Figure 11 compares the atomic structure of antiparallel pleated and rippled β-sheets that formed spontaneously in our simulations of **L1/L1** and **L1/D1** peptides. In both architectures, the backbone of each individual peptide adopts the corrugated (i.e., zigzag-like) conformation that is characteristic of extended β-strands, with side chains projected out of the β-sheet plane. This projection alternates between the two faces of the sheet, segregating phenylalanine (gray color in Figure 11) to one face and charged side chains (orange and cyan) to the other. The direction of backbone amine and carbonyl groups also alternates along the strand as it depends on the orientation of the side chain (Figure 11C). This alternation is indicated by brown arrows in Figure 11D for a pair of antiparallel **L1** peptides and in Figure 11E for **L1** and **D1** peptides oriented in opposite directions. The formation of interstrand hydrogen bonds requires amine and carbonyl groups from adjacent peptides to face one another. For two antiparallel **L1** peptides, this occurs between residues whose side chains point in the same direction, leading to phenylalanine–phenylalanine or charge–charge pairing (Figure 11D). In contrast, for antiparallel **L1** and **D1** peptides, hydrogen bonding occurs between residues with oppositely oriented side chains, pairing phenylalanine with charged residues (Figure 11E). In pleated β-sheets, pairing of like residues synchronizes the corrugated backbone pattern across neighboring strands (Figure 11A), whereas in rippled β-sheets, mismatched residue pairing leads to out-of-phase corrugation, resulting in the less pronounced zigzag pattern in Figure 11B.

**Figure 11:**
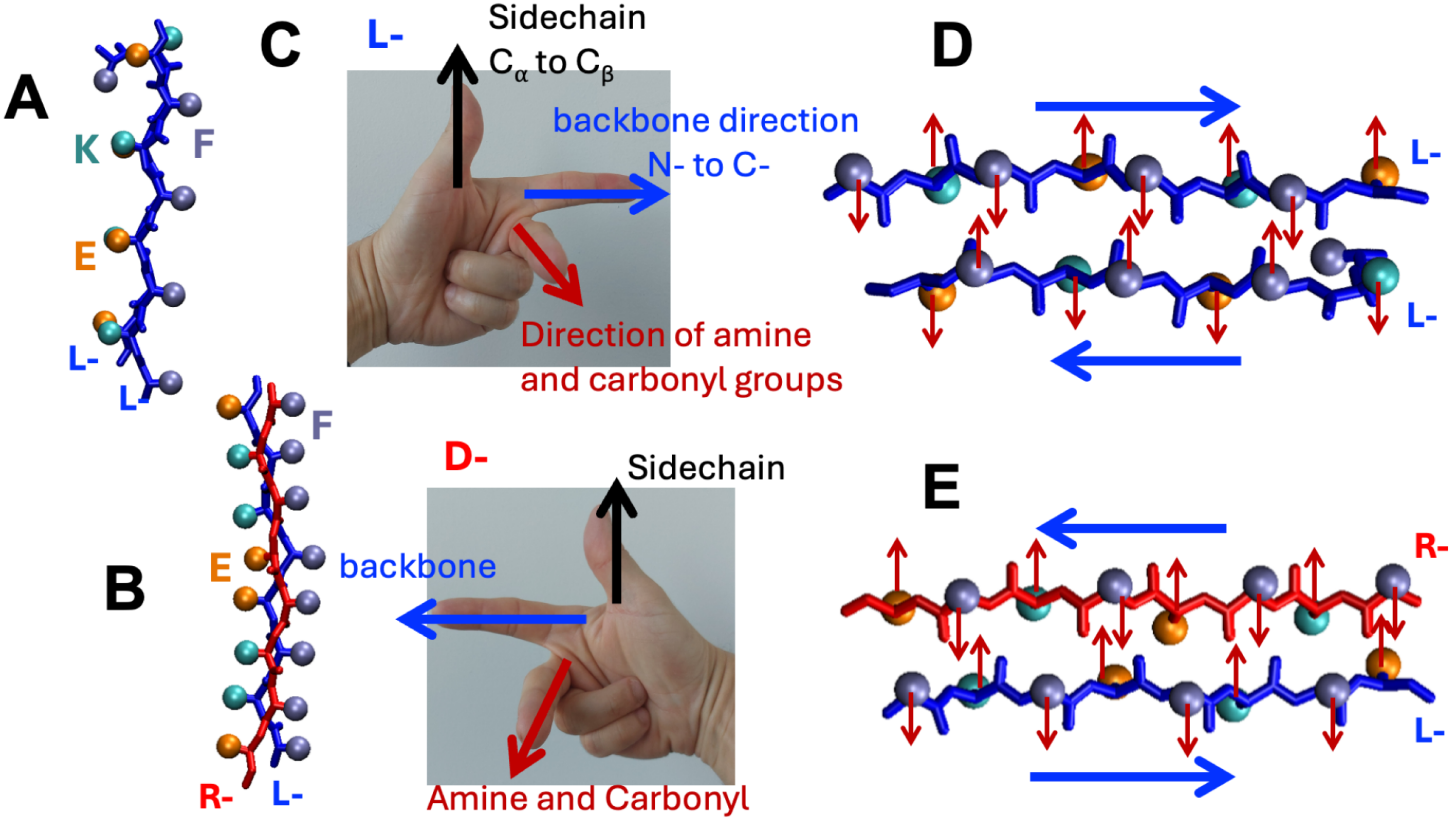
Corrugated pattern of the backbone in (A) pleated and (B) rippled β-sheets. (C) The direction of backbone amine and carbonyl groups can be determined by the orientation of the middle finger when the index finger points along the peptide sequence (from N- to C- termini) and the thumb points toward the side chain (from C_α_ to C_β_ atoms). Left and right hands are used for l-enantiomer and d-enantiomer peptides. Brown arrows represent the direction of backbone amine and carbonyl groups of (D) two antiparallel l-peptides and (E) antiparallel l- and d-peptides.

Notice that the space available to accommodate side chains is the same in rippled and pleated β-sheets. However, in pleated β-sheets, side chains lying on the same face of the sheet pair up at a closer distance than in rippled β-sheets where they are staggered (Figure 1). In our simulations, the average distance between C_β_ atoms in nearby phenylalanine and nearby charged residues is larger in rippled compared to pleated β-sheets by 0.1 and 0.07 nm, respectively. These larger distances can better accommodate bulky side chains, and we speculate that they may affect the solvation of exposed side chains.^74^ Also, the pattern of staggered side chains in rippled β-sheets may affect electrostatic interactions of charged side chains and aromatic interactions. These different factors may account for the preference of rippled over pleated β-sheets.

## Discussion

Collectively, the experimental and computational work presented herein support a marked preference for the formation of rippled β-sheets from racemic mixtures of enantiomers compared to pleated β-sheets of single enantiomers. This effect was universal for all peptide sequences studied. We contend that the thermodynamic preference for rippled β-sheet formation is due to the altered packing conformation that has been proposed^11^ and observed^28–32^ for rippled β-sheets (Figure 1). Pleated β-sheet structure features an eclipsed alignment of side chain groups in cross-strand positions, while rippled β-sheet structure has an alternative staggered conformation. We propose that the more relaxed side chain packing in rippled β-sheets reduces steric crowding compared to that experienced in pleated β-sheets, thus providing a thermodynamic advantage for coassembly of enantiomeric peptides.

While these studies support a general preference for rippled β-sheet versus pleated β-sheet formation, we cannot yet conclude that this preference is universal for all peptide sequences. For example, work from Nowick^36,38^ and Gellman^37^ show that in templated peptide systems, pleated β-sheet formation is favored over rippled β-sheet formation. A major difference in the systems we have studied herein and in the Nowick and Gellman systems is the constraint of the peptide conformation. For example, Nowick demonstrated with hydrazide-templated dimers that enantiomers self-sorted into homochiral pleated β-sheets with a preference of 3.1–4.2 kcal mol^–1^ compared to heterochiral pairing.^36^ They likewise demonstrated a preference for homochiral pleated β-sheet self-sorting in templated amyloid-β fragments over heterochiral rippled β-sheet formation. Gellman and coworkers likewise demonstrated that folding in hairpin systems in which paired strands are either homochiral (pleated β-sheet hairpin) or heterochiral (rippled β-sheet hairpin) occurs preferentially when the templated strands are homochiral. It is possible that in these systems, the conformational constraints that have been introduced influence the energetics of pleated versus rippled β-sheet interaction, introducing energetic penalties that disfavor rippled β-sheet formation. In the systems reported herein, no conformational constraints exist. We hypothesize that this lack of conformational constraint enables penalty-free assembly into rippled β-sheet structures with thermodynamic advantage.

Another point of interest in these studies is that the rippled β-sheet assemblies often have polymorphic appearances. For example, the pleated β-sheet nanoribbons and nanotubes observed from solutions of **L1** are highly ordered and are conserved across all samples. In contrast, the putative rippled β-sheets of **L1/D1** mixtures are polymorphic in appearance. Instead of fibrils that have uniform widths across the entire length of the fibril, we instead observe fibrils in which width measurements vary along the length of the fibril. We propose that the energetically favorable rippled β-sheet packing structure reduces the capacity of peptide strands that are added to fibril ends to come on and off the fibril until the most energetically preferred conformation is achieved. That is, the less energetically favorable pleated β-sheet conformation results in more uniform assemblies because, as a new peptide is added to an assembly, the peptide can readily undergo conformational rearrangements until the most energetically favorable alignment is achieved. This type of rearrangement may be less common in rippled β-sheet conformations because any alignment is sufficiently energetically favorable that disengagement and rearrangement to a more energetically ideal arrangement is less common.

The all-atom molecular dynamics simulations presented in this study are, to our knowledge, the first to describe the unbiased coassembly of equimolar mixtures of l- and d-peptides into rippled β-sheets. The trend of peptide aggregation extracted from these simulations follows the sequence dependence **L1/D1 > L2/D2 > L3/D3 > L4/D4 ≈ L5/D5**, in agreement with the experimentally observed propensity of these peptides to form rippled β-sheet architectures. These findings complement our group’s recent homochiral simulations, which demonstrated that **L1** peptides form cross-β structures more rapidly than **L3**, which in turn aggregate faster than **L4**, highlighting a consistent sequence-dependent trend across both racemic and homochiral systems.^41^ Recently, our group has also employed unbiased all-atom simulations of homochiral **L1** peptides to investigate fibril elongation mechanisms.^75,76^ Although these simulations are computationally demanding, extending this approach to racemic systems would be highly informative, as it would enable a direct comparison between simulation results and the experimentally measured free energies of elongation reported in this work.

Finally, the polymorphism of putative rippled β-sheets has complicated our early efforts to obtain structural evidence for the rippled β-sheet fold in these systems. We have initiated efforts to use cryo-EM analysis to study the structure of these materials. While highly ordered pleated β-sheet assemblies have been successfully defined using cryo-EM,^20^ thus far, the polymorphic nature of the rippled β-sheets have complicated our own efforts to study these materials. These efforts are ongoing, and we hope to be able to provide structural evidence for the rippled β-sheet structure in these materials in due course. Absent this level of structural evidence for the rippled β-sheet structure in these materials, we believe the evidence strongly supports rippled β-sheet formation in the sequence isomers reported herein. Additional studies will undoubtedly continue to illuminate our understanding of both rippled and pleated β-sheets in the context of supramolecular self-assembly.

## Conclusion

Herein, we have compared the supramolecular assembly properties of a range of sequence isomer peptides to the assembly properties of racemic mixtures of these peptides with their enantiomers. These studies have shown that for all sequences, there is a strong preference for coassembly of the enantiomers compared to self-assembly of the enantiopure counterparts. Evidence for these observed preferences is in the form of spectroscopic analysis, morphological differences in the homochiral and heterochiral assemblies, and strong thermodynamic advantages for coassembly of enantiomers relative to self-assembly of homochiral sequences. Unbiased all-atom molecular dynamics simulations agreed with experimental analysis, showing increased β-sheet content for rippled β-sheet systems compared to pleated β-sheet systems. Collectively, this data supports a preference for supramolecular rippled β-sheet formation relative to pleated β-sheet formation in these peptide systems. Isotope-edited FTIR experiments support the adoption of alternating l/d-peptide arrangements in the putative rippled β-sheets formed from mixtures of enantiomers. These studies provide valuable insight into the scope of pleated and rippled β-sheet formation and confirm the thermodynamic preference for rippled β-sheets in unconstrained systems. Future investigations will focus on elucidation of the molecular basis for these observations by seeking high-resolution structural confirmation of rippled β-sheets in these peptide systems. Current and future work will facilitate the exciting design and use of rippled β-sheets in novel next-generation materials.

## Supplementary Material



## Data Availability

All primary data are available from the corresponding authors upon request.

## Notes

Peptide characterization data (MALDI-TOF mass spectra; HPLC purification conditions and traces); peptide concentration curves; additional FTIR spectra (including spectra analyzed with Lorentzian line fitting) (PDF)
