# Supplementary material for: Comparison of Pleated and Rippled β‑Sheet Assembly of Sequence Isomers of an Amphipathic Self-Assembling Peptide
Source: Biochemistry. 2026 Mar 20;65(7):994–1012. doi: 10.1021/acs.biochem.6c00035 (PMC13063417; doi:10.1021/acs.biochem.6c00035)
Supplement: Supplementary file 1 [file bi6c00035_si_001.pdf]

Supporting Information

Comparison of Pleated and Rippled β-Sheet Assembly of Sequence Isomers of an Amphipathic Self-Assembling Peptide

*Christopher W. Jones,[a] Jianping Chen,[b] Rishab Panda,[a] Sharareh Jalali,[b] Loren P. Cardani,[a] Yahui Guo,[a] Ian M. Arnold,[a] Cristiano L. Dias,[b] and Bradley L. Nilsson[a,c]**

[a] Department of Chemistry, University of Rochester, Rochester, NY, 14627-0216, USA

[b] Department of Physics, New Jersey Institute of Technology, Newark, NJ 07102-1982, USA

[c] Materials Science Program, University of Rochester, Rochester, NY 14627-0166, USA

E-mail: bradley.nilsson@rochester.edu

Tel. +1 585 276-3053

Analytical HPLC Traces **6–13**
Figure S1. Ac-$(FKFE)_2$-NH_2 6
Figure S2. Ac-$(FK)_2(FE)_2$-NH_2 6
Figure S3. Ac-KE$(F)_4$KE-NH_2 7
Figure S4. Ac-$(KFFE)_2$-NH_2 7
Figure S5. Ac-FF$(KE)_2$FF-NH_2 8
Figure S6. Ac-$(fkfe)_2$-NH_2 8
Figure S7. Ac-$(fk)_2(fe)_2$-NH_2 9
Figure S8. Ac-ke$(f)_4$ke-NH_2 9
Figure S9. Ac-$(kffe)_2$-NH_2 10
Figure S10. Ac-ff$(ke)_2$ff-NH_2 10
Figure S11. Ac-F*KFEFKFE-NH_2 11
Figure S12. Ac-F*KFEFKF*E-NH_2 11
Figure S13. Ac-F*KFKFEFE-NH_2 12
Figure S14. Ac-F*KFKFEF*E-NH_2 12
Figure S15. Ac-KEF*FFFKE-NH_2 13
Figure S16. Ac-KEF*FFF*KE-NH_2 13
Peptide Purification Details **14–15**
Table S1. Interchim Prep HPLC Retention Times 14
Table S2. Interchim Prep HPLC Gradient Conditions for L1 14
Table S3. Interchim Prep HPLC Gradient Conditions for L2 14
Table S4. Interchim Prep HPLC Gradient Conditions for L3 14
Table S5. Interchim Prep HPLC Gradient Conditions for L4 15
Table S6. Interchim Prep HPLC Gradient Conditions for L5 15
Table S7. Analytical HPLC Retention Times 15
Table S8. Analytical HPLC Gradient Conditions 15
MALDI-TOF-MS Spectra **16–24**
Figure S17. Ac-$(FKFE)_2$-NH_2 16
Figure S18. Ac-$(FK)_2(FE)_2$-NH_2 16
Figure S19. Ac-KE$(F)_4$KE-NH_2 17

Figure S20. Ac-$(KFFE)_2$-NH_2 17
Figure S21. Ac-FF$(KE)_2$FF-NH_2 18
Figure S22. Ac-$(fkfe)_2$-NH_2 18
Figure S23. Ac-$(fk)_2(fe)_2$-NH_2 19
Figure S24. Ac-ke$(f)_4$ke-NH_2 19
Figure S25. Ac-$(kffe)_2$-NH_2 20
Figure S26. Ac-ff$(ke)_2$ff-NH_2 20
Figure S27. Ac-F*KFEFKFE-NH_2 21
Figure S28. Ac-F*KFEFKF*E-NH_2 21
Figure S29. Ac-F*KFKFEFE-NH_2 22
Figure S30. Ac-F*KFKFEF*E-NH_2 22
Figure S31. Ac-KEF*FFFKE-NH_2 23
Figure S32. Ac-KEF*FFF*KE-NH_2 23
Table S9. Calculated and Observed Masses 24
HPLC Calibration Curves 24–26
Figure S33. Ac-$(FKFE)_2$-NH_2 24
Figure S34. Ac-$(FK)_2(FE)_2$-NH_2 25
Figure S35. Ac-KE$(F)_4$KE-NH_2 25
Figure S36. Ac-$(KFFE)_2$-NH_2 26
Figure S37. Ac-FF$(KE)_2$FF-NH_2 26
FTIR Timepoints 27–31
Figure S38. L1 1 mM 27
Figure S39. L1/D1 1 mM 27
Figure S40. L2 1 mM 28
Figure S41. L2/D2 1 mM 28
Figure S42. L3 1 mM, 2 mM, and 4 mM 29
Figure S43. L3/D3 1 mM 29
Figure S44. L4 1 mM, 2 mM, and 4 mM 30

Figure S45. L4/D4 1 mM, 2 mM. and 4 mM 30
Figure S46. L5 1 mM, 2 mM, and 4 mM 31
Figure S47. L5/D5 1 mM, 2 mM, and 4 mM 31
FTIR Timepoints with Peak Fitting 32–51
Figure S48. L1 1 mM 32
Figure S49. L1/D1 1 mM 33
Figure S50. L2 1 mM 34
Figure S51. L2/D2 1 mM 35
Figure S52. L3 1 mM 36
Figure S53. L3 2 mM 37
Figure S54. L3 4 mM 38
Figure S55. L3/D3 1 mM 39
Figure S56. L4 1 mM 40
Figure S57. L4 2 mM 41
Figure S58. L4 4 mM 42
Figure S59. L4/D4 1 mM 43
Figure S60. L4/D4 2 mM 44
Figure S61. L4/D4 4 mM 45
Figure S62. L5 1 mM 46
Figure S63. L5 2 mM 47
Figure S64. L5 4 mM 48
Figure S65. L5/D5 1 mM 49
Figure S66. L5/D5 2 mM 50
Figure S67. L5/D5 4 mM 51
Lorentzian Function Details 52
Peak Fitting Code 53–57
CD Spectra 58–68
Figure S68. L1 1 mM 58

Figure S69. L1/D1 1 mM....58
Figure S70. L2 1 mM....59
Figure S71. L2/D2 1 mM....59
Figure S72. L3 1 mM....60
Figure S73. L3 2 mM....60
Figure S74. L3 4 mM....61
Figure S75. L3/D3 1 mM....61
Figure S76. L4 1 mM....62
Figure S77. L4 2 mM....62
Figure S78. L4 4 mM....63
Figure S79. L4/D4 1 mM....63
Figure S80. L4/D4 2 mM....64
Figure S81. L4/D4 4 mM....64
Figure S82. L5 1 mM....65
Figure S83. L5 2 mM....65
Figure S84. L5 4 mM....66
Figure S85. L5/D5 1 mM....66
Figure S86. L5/D5 2 mM....67
Figure S87. L5/D5 4 mM....67
Table S10. Peptide Assembly and Working Concentrations....68
J-Parameter Details....68–69
Figure S88. J-Parameter Distribution....69

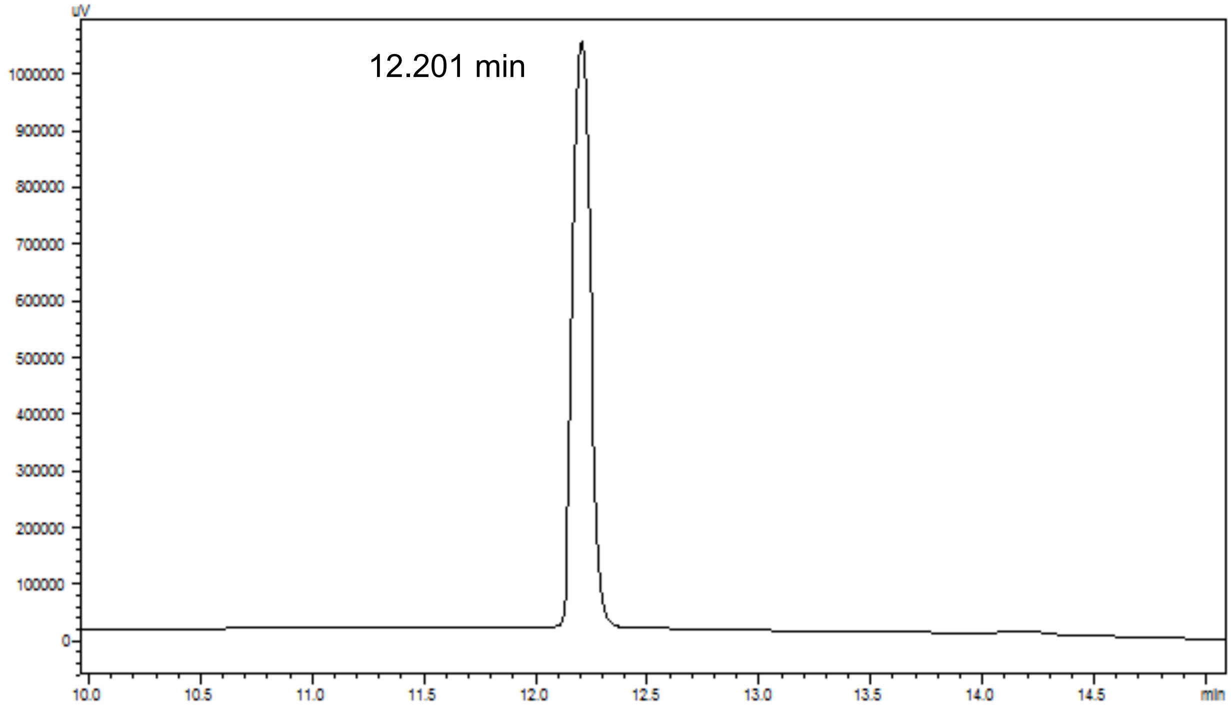

Figure S1. Analytical HPLC chromatogram of Ac-(FKFE)$_2$-NH$_2$ (L1)

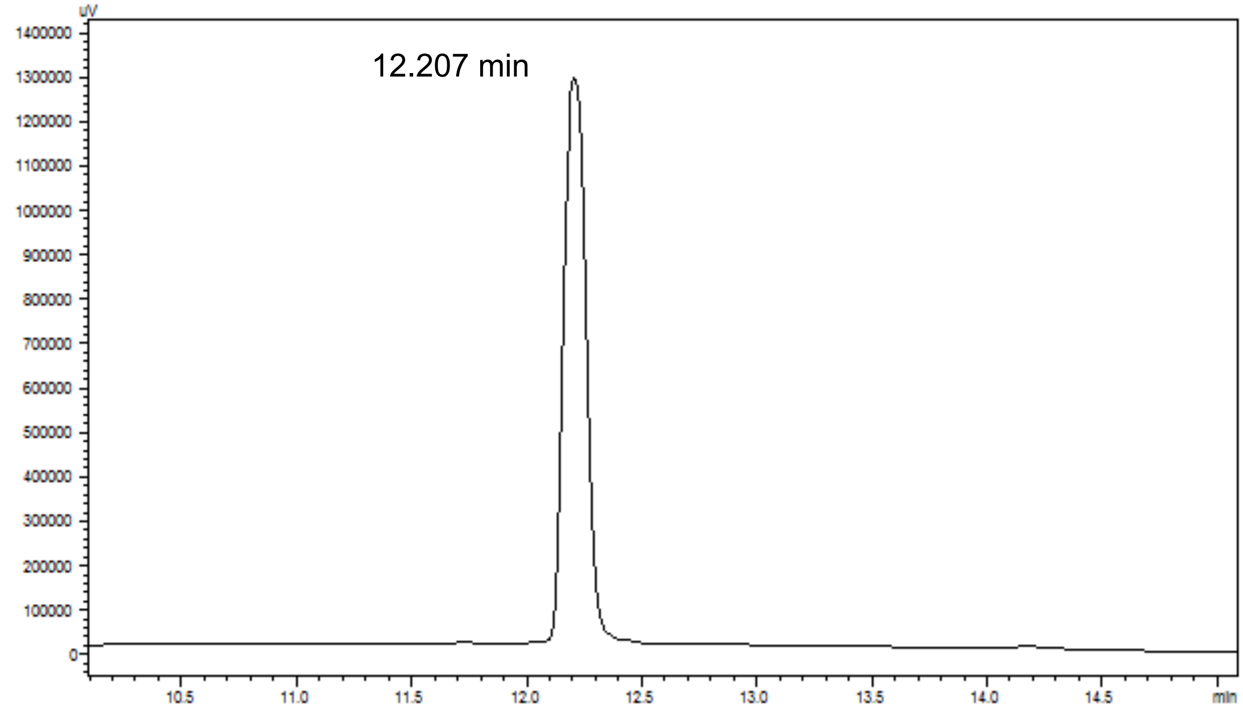

Figure S2. Analytical HPLC chromatogram of Ac-(FK)$_2$(FE)$_2$-NH$_2$ (L2)

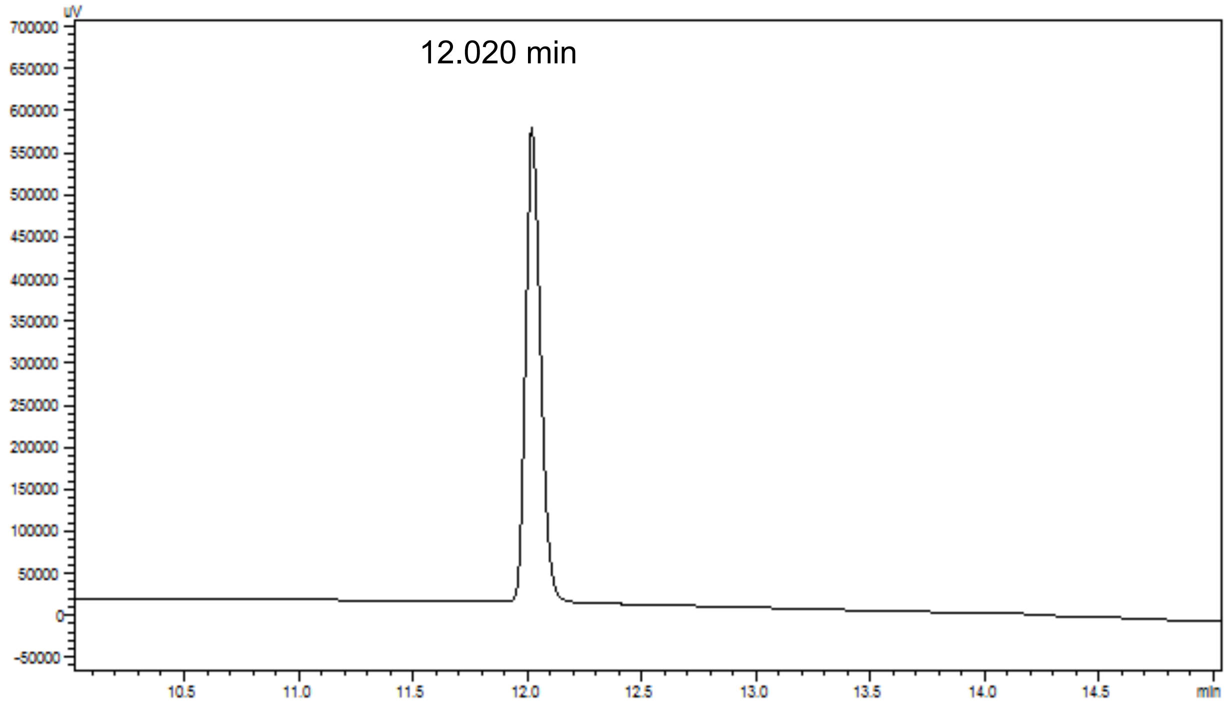

Figure S3. Analytical HPLC chromatogram of Ac-KE(F)$_4$KE-NH$_2$ (L3)

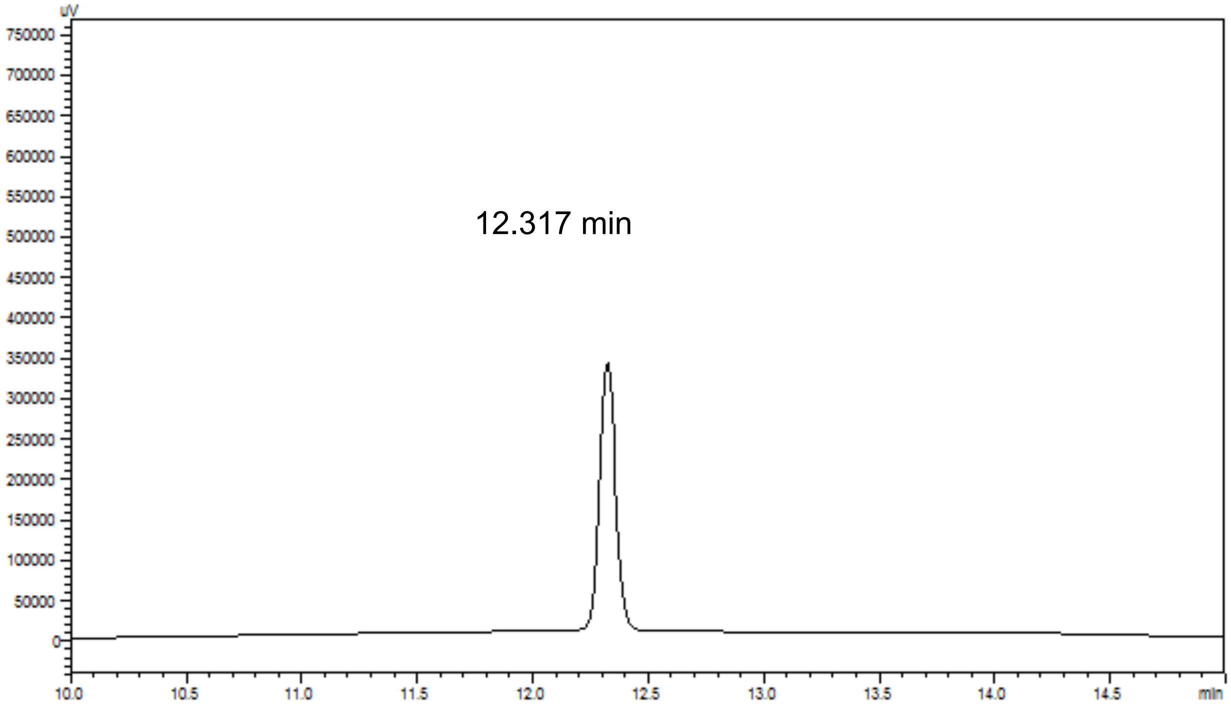

Figure S4. Analytical HPLC chromatogram of Ac-(KFFE)$_2$-NH$_2$ (L4)

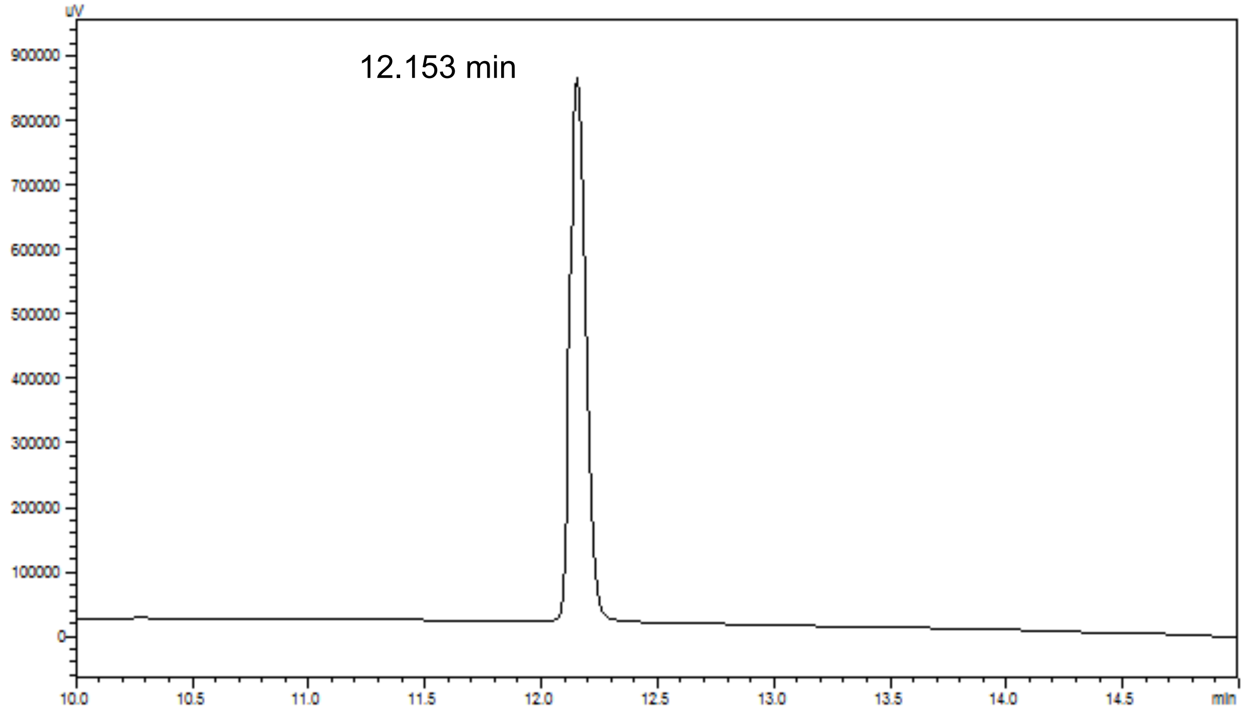

Figure S5. Analytical HPLC chromatogram of Ac-FF(KE)$_2$FF-NH$_2$ (L5)

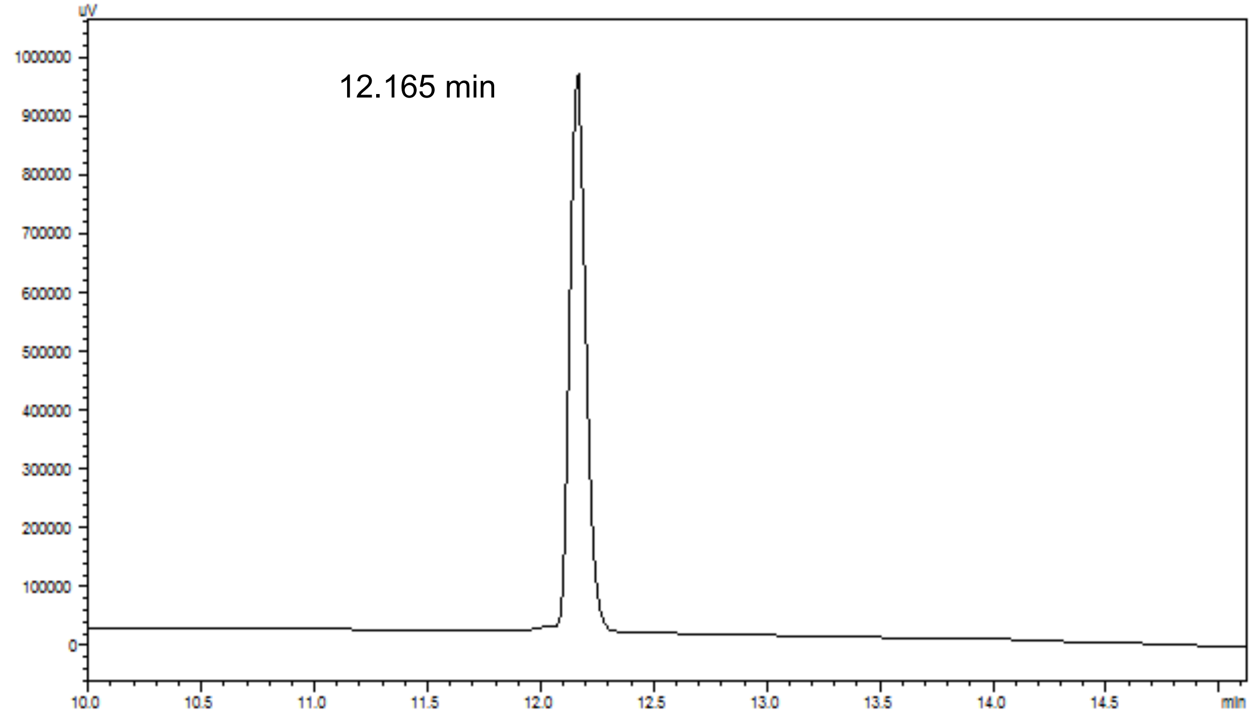

Figure S6. Analytical HPLC chromatogram of Ac-(fkfe)$_2$-NH$_2$ (D1)

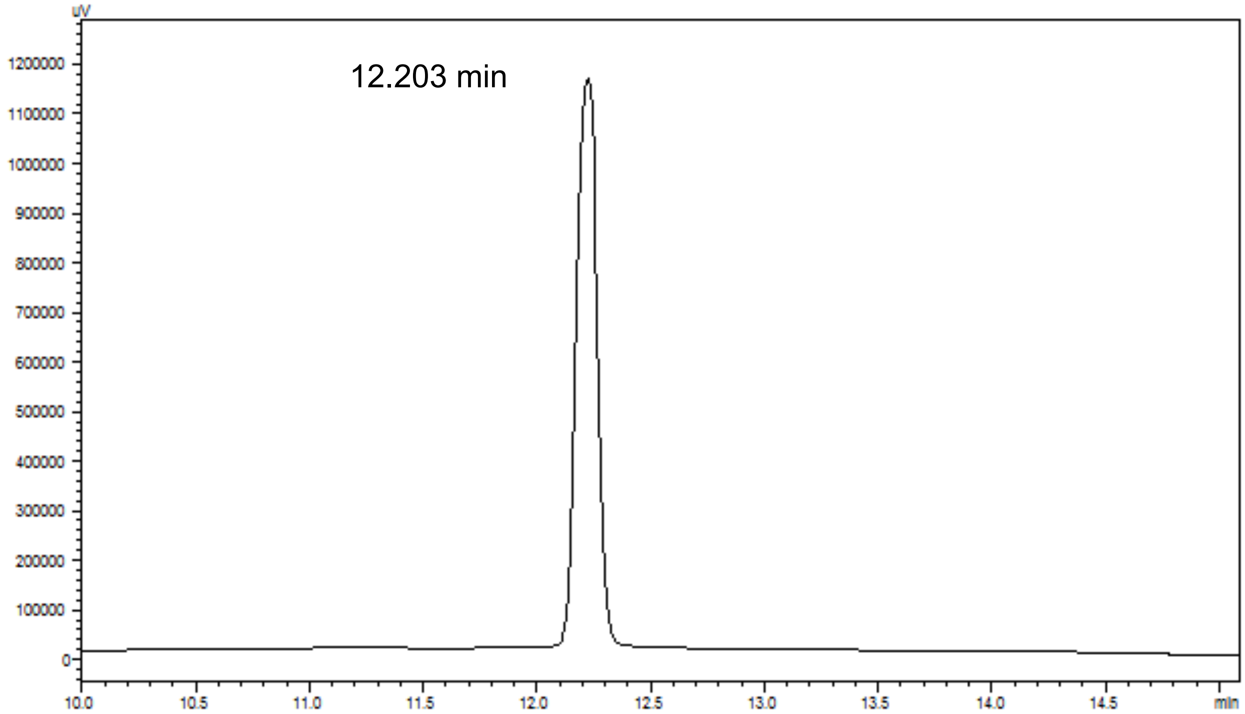

Figure S7. Analytical HPLC chromatogram of Ac-(fk)$_2$(fe)$_2$-NH_2 (D2)

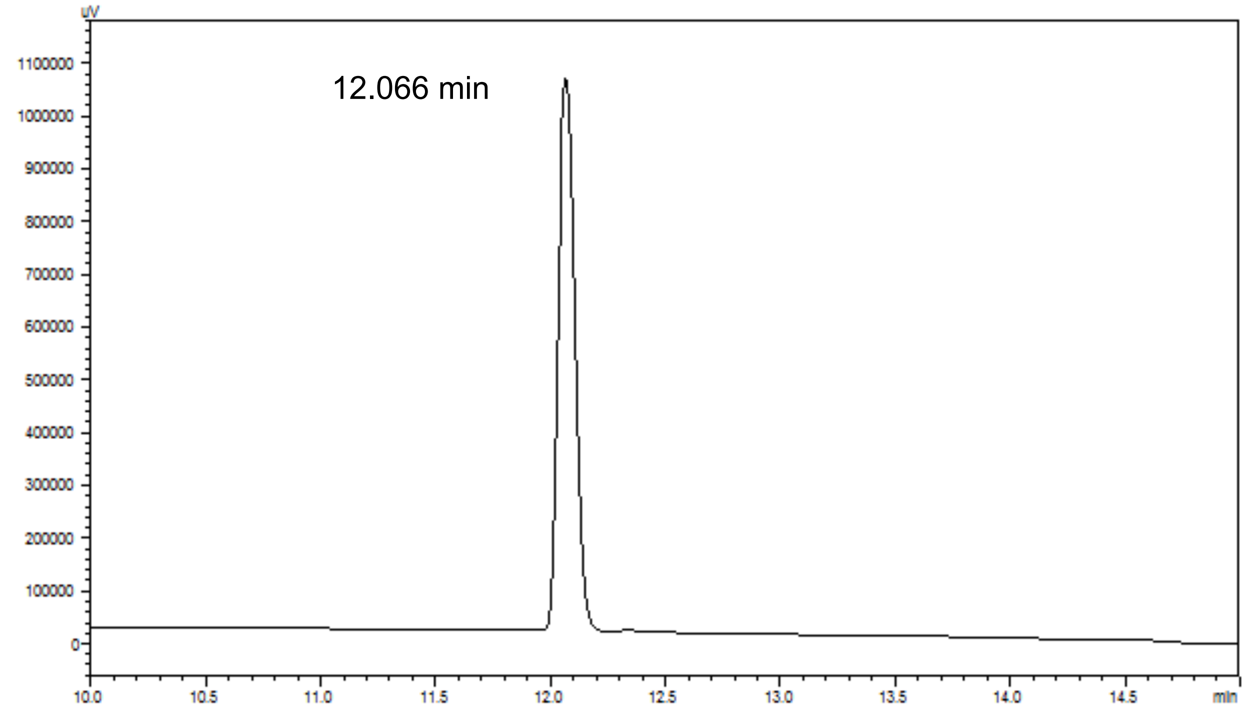

Figure S8. Analytical HPLC chromatogram of Ac-ke(f)$_4$ke-NH_2 (D3)

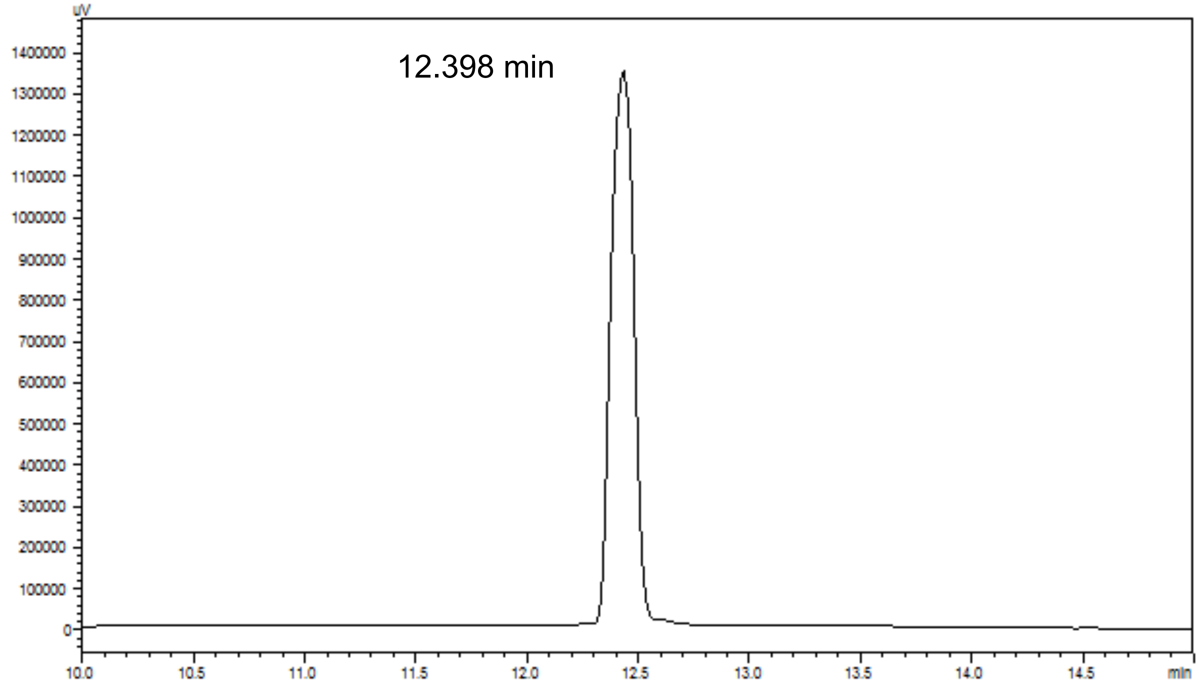

Figure S9. Analytical HPLC chromatogram of Ac-(kffe)$_2$-NH$_2$ (D4)

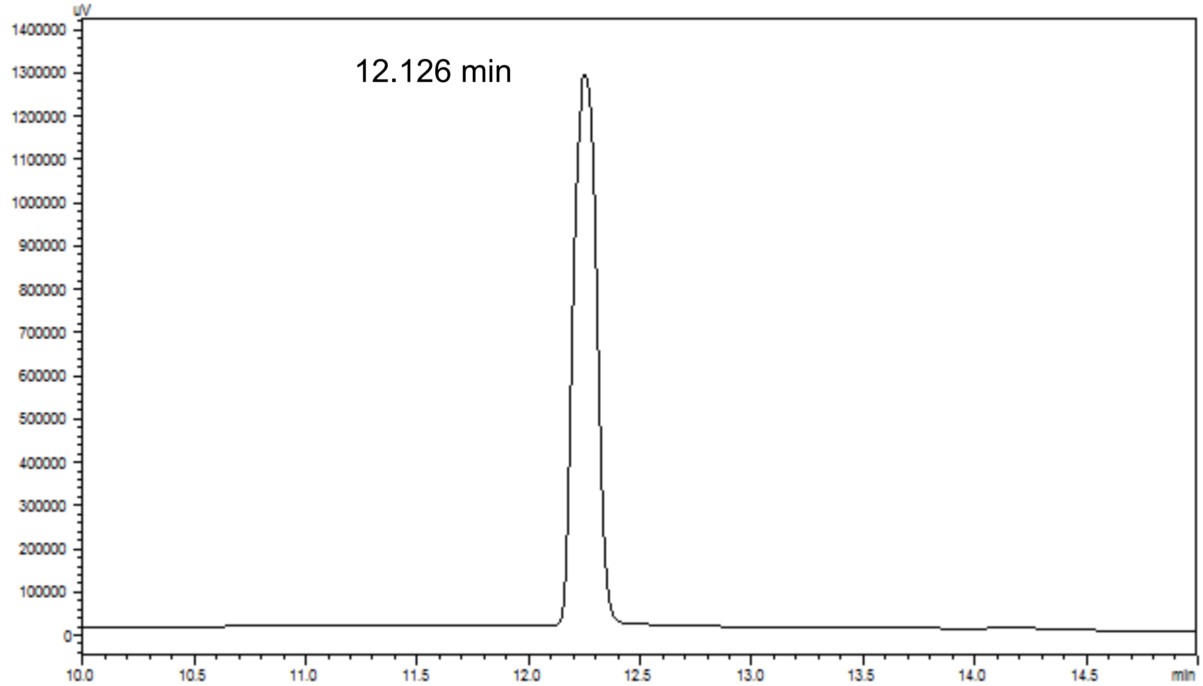

Figure S10. Analytical HPLC chromatogram of Ac-ff(ke)$_2$ff-NH$_2$ (D5)

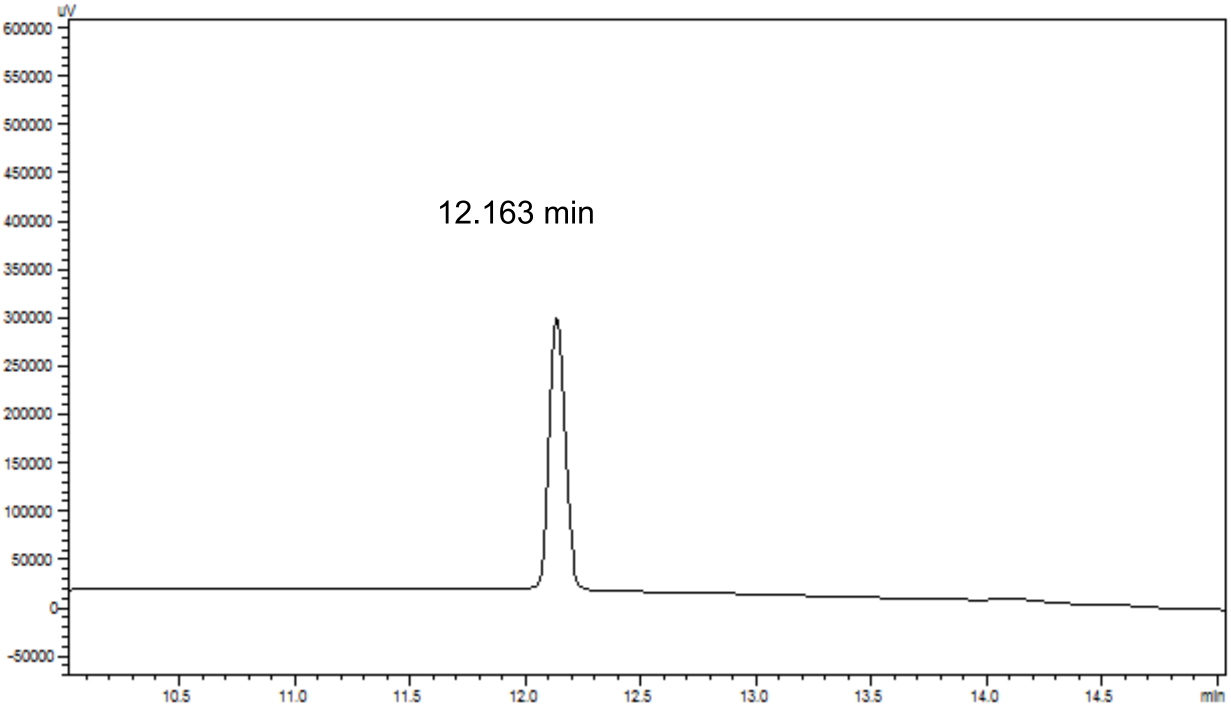

Figure S11. Analytical HPLC chromatogram of Ac-F*KFEFKFE-NH_2 (L1X)

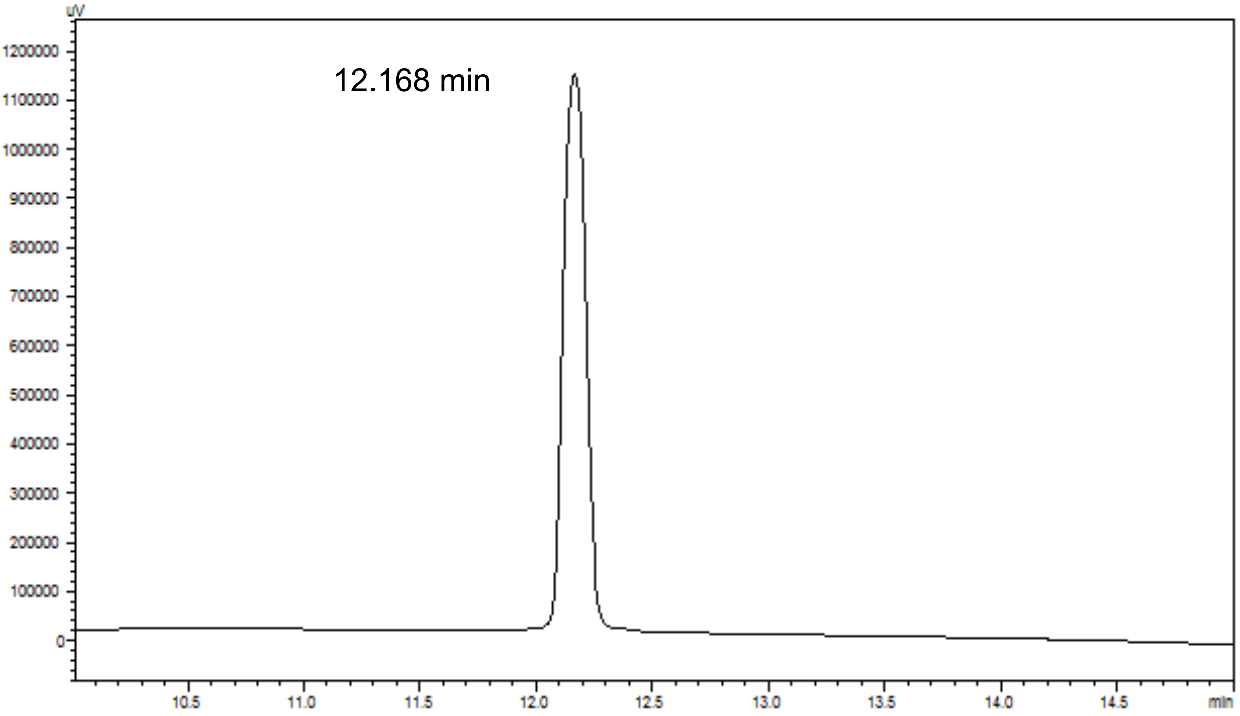

Figure S12. Analytical HPLC chromatogram of Ac-F*KFEFKF*E-NH_2 (L1XX)

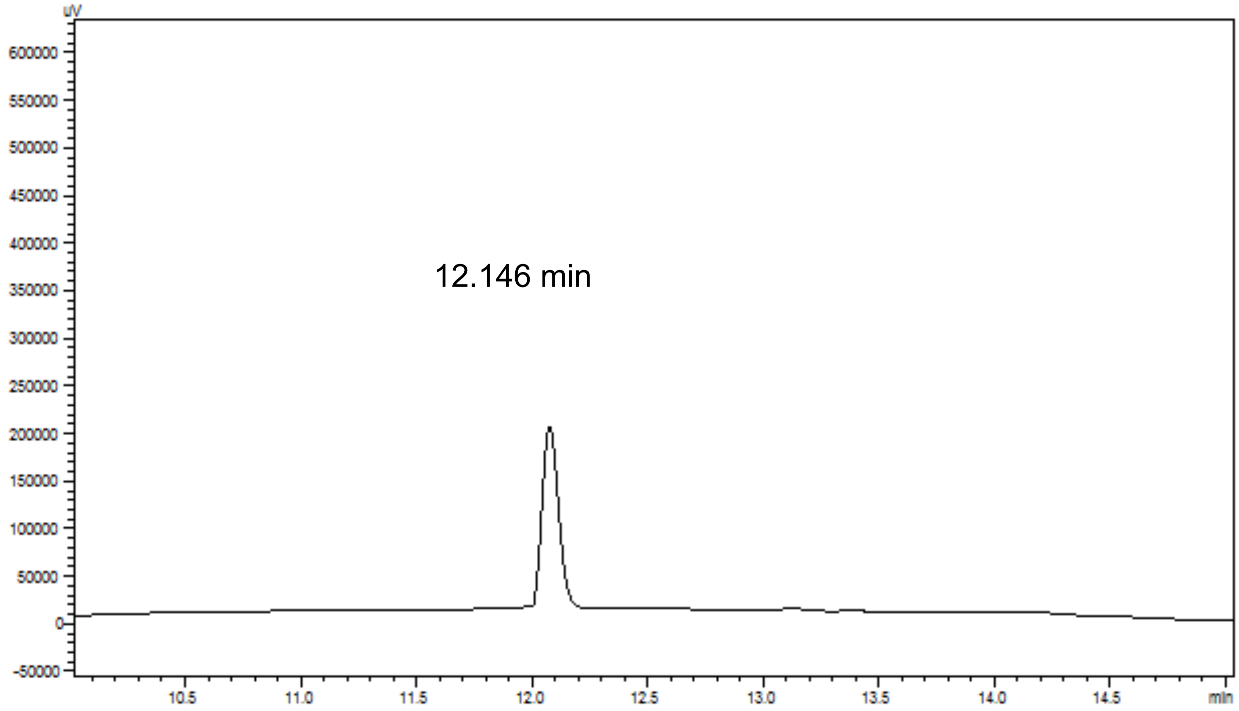

Figure S13. Analytical HPLC chromatogram of Ac-F*KFKFEFE-NH_2 (L2X)

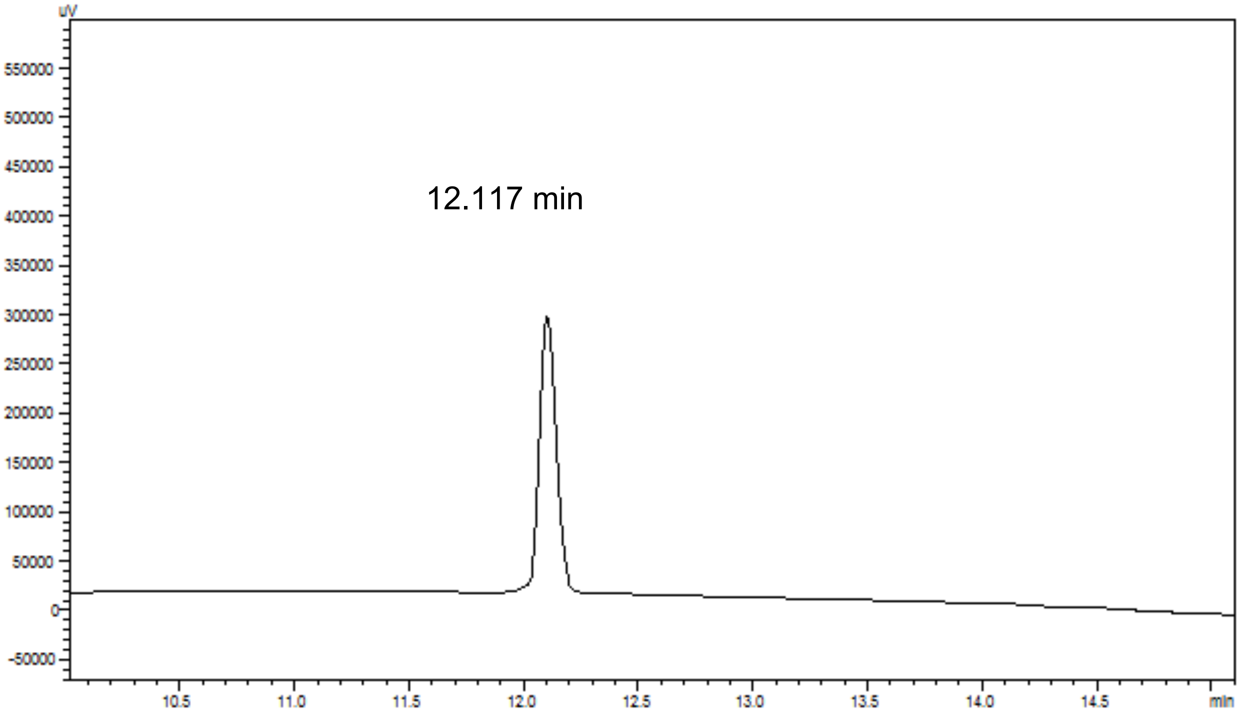

Figure S14. Analytical HPLC chromatogram of Ac-F*KFKFEF*E-NH_2 (L2XX)

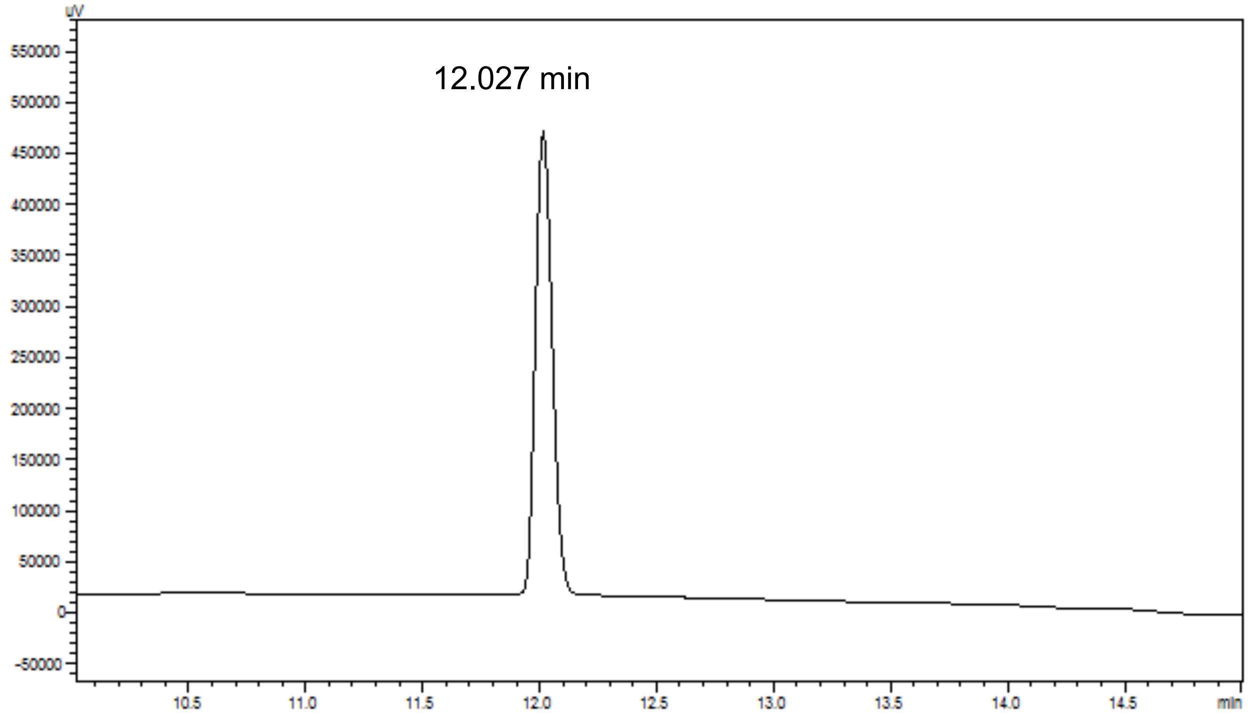

Figure S15. Analytical HPLC chromatogram of Ac-KEF*FFFKE-NH_2 (L3X)

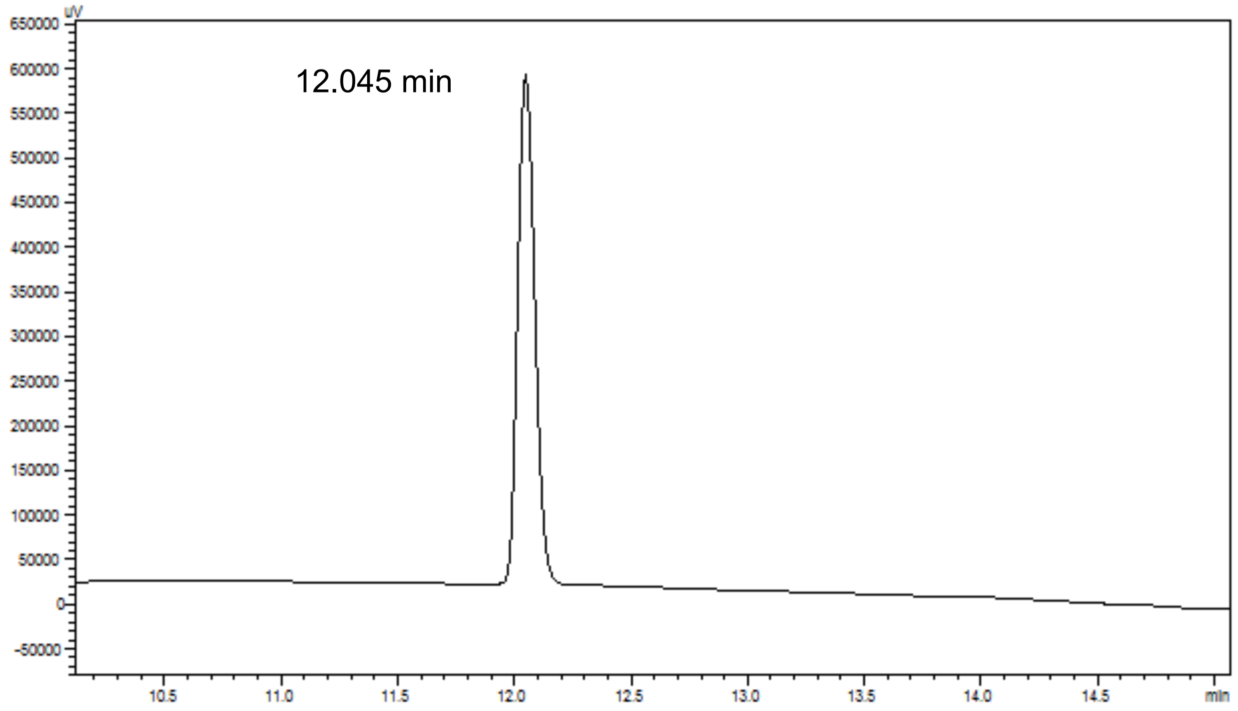

Figure S16. Analytical HPLC chromatogram of Ac-KEF*FFF*KE-NH_2 (L3XX)

Table S1. Interchim Prep HPLC Retention Times

Peptide	Sequence	Retention Time (Min)
L1, D1, L1X, L1XX	Ac-(FKFE)$_2$-NH$_2$	~17:06
L2, D2, L2X, L2XX	Ac-(FK)$_2$(FE)$_2$-NH$_2$	~12:04
L3, D3, D3X, D3XX	Ac-KE(F)$_4$KE-NH$_2$	~17:01
L4, D4	Ac-(KFFE)$_2$-NH$_2$	~9:39
L5, D5	Ac-FF(KE)$_2$FF-NH$_2$	~18:39

Table S2. Interchim Prep HPLC Gradient Conditions for L1

Time	Flow rate (mL/min)	% Water	% Acetonitrile
0:00	100	70	30
8:30	100	65	35
9:30	100	5	95
14:00	100	5	95
15:00	100	70	30
19:30	100	70	30

Table S3. Interchim Prep HPLC Gradient Conditions for L2

Time	Flow rate (mL/min)	% Water	% Acetonitrile
0:00	100	80	20
15:30	100	55	45
16:30	100	5	95
21:00	100	5	95
22:00	100	80	20
26:30	100	80	20

Table S4. Interchim Prep HPLC Gradient Conditions for L3

Time	Flow rate (mL/min)	% Water	% Acetonitrile
0:00	100	80	20
15:30	100	70	30
16:30	100	5	95
21:00	100	5	95
22:00	100	80	20
26:30	100	80	20

Table S5. Interchim Prep HPLC Gradient Conditions for L4

Time	Flow rate (mL/min)	% Water	% Acetonitrile
0:00	100	75	25
15:30	100	60	40
16:30	100	5	95
21:00	100	5	95
22:00	100	75	25
26:30	100	75	25

Table S6. Interchim Prep HPLC Gradient Conditions for L5

Time	Flow rate (mL/min)	% Water	% Acetonitrile
0:00	100	80	20
15:30	100	55	45
16:30	100	5	95
21:00	100	5	95
22:00	100	80	20
26:30	100	80	20

Table S7. Analytical HPLC Retention Times

Peptide	Sequence	Retention Time (Min)
L1, D1, L1X, L1XX	Ac-(FKFE)$_2$-NH$_2$	12.201, 12.165, 12.163, 12.168
L2, D2, L2X, L2XX	Ac-(FK)$_2$(FE)$_2$-NH$_2$	12.207, 12.203, 12.146, 12.117
L3, D3, D3X, D3XX	Ac-KE(F)$_4$KE-NH$_2$	12.020, 12.066, 12.027, 12.045
L4, D4	Ac-(KFFE)$_2$-NH$_2$	12.317, 12.398
L5, D5	Ac-FF(KE)$_2$FF-NH$_2$	12.153, 12.126

Table S8. Analytical HPLC Gradient Conditions

Time	Flow Rate (mL/Min)	% Water	% Acetonitrile
0:00	1	95	5
5:00	1	95	5
15:00	1	5	95
20:00	1	5	95
22:00	1	95	5
25:00	1	95	5

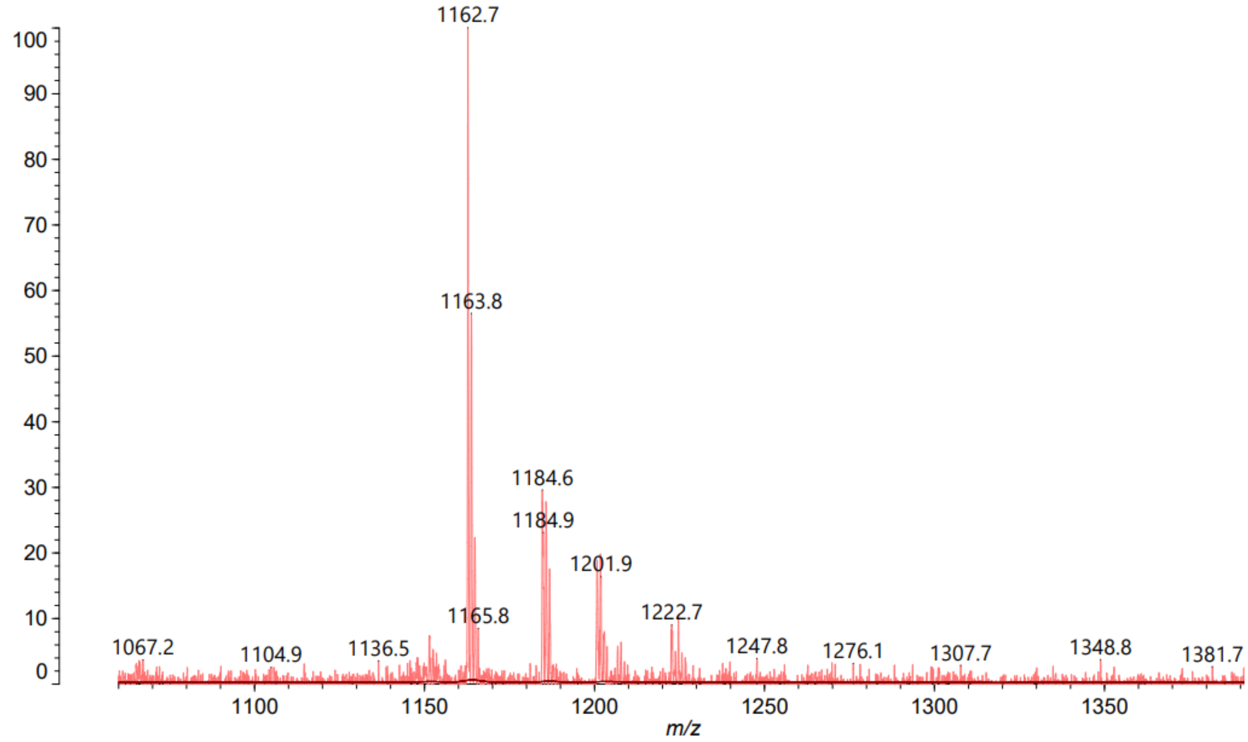

Figure S17. MALDI-TOF spectrum of Ac-$(FKFE)_2$-NH_2 (L1)

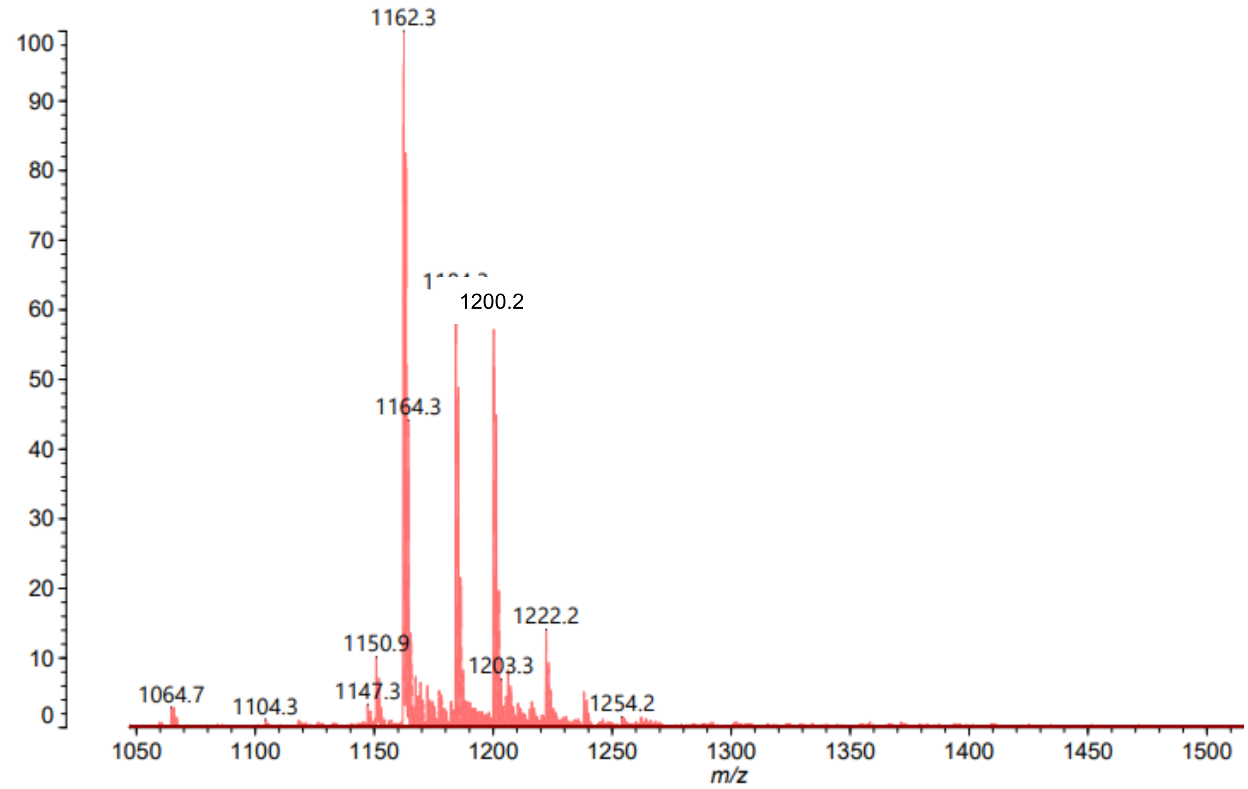

Figure S18. MALDI-TOF spectrum of Ac-$(FK)_2(FE)_2$-NH_2 (L2)

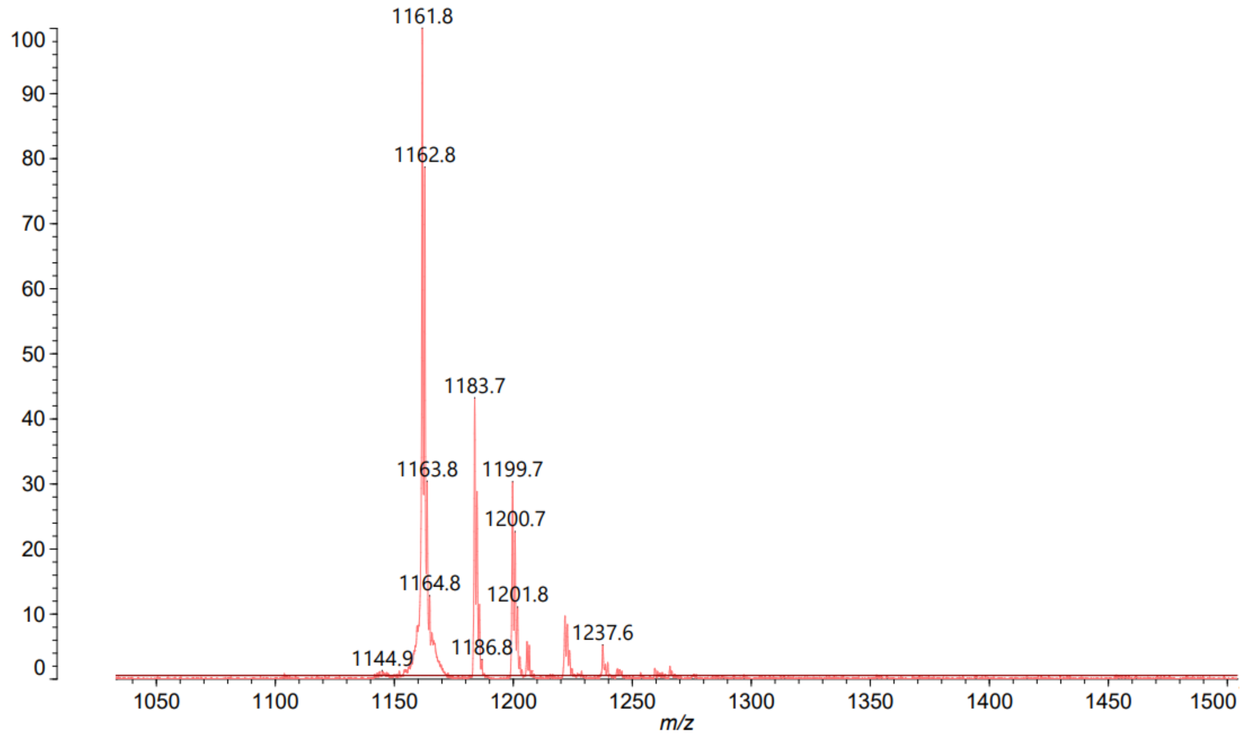

Figure S19. MALDI-TOF spectrum of Ac-KE(F)$_4$KE-NH$_2$ (L3)

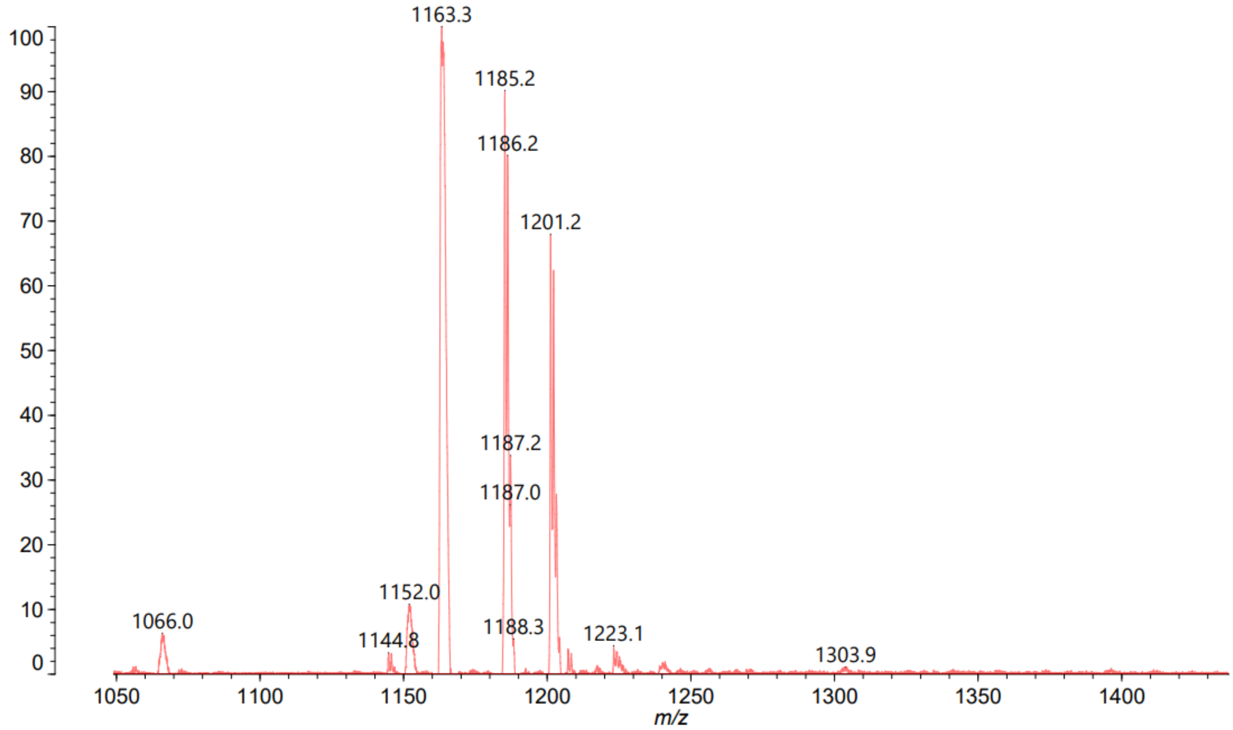

Figure S20. MALDI-TOF spectrum of Ac-(KFFE)$_2$-NH$_2$ (L4)

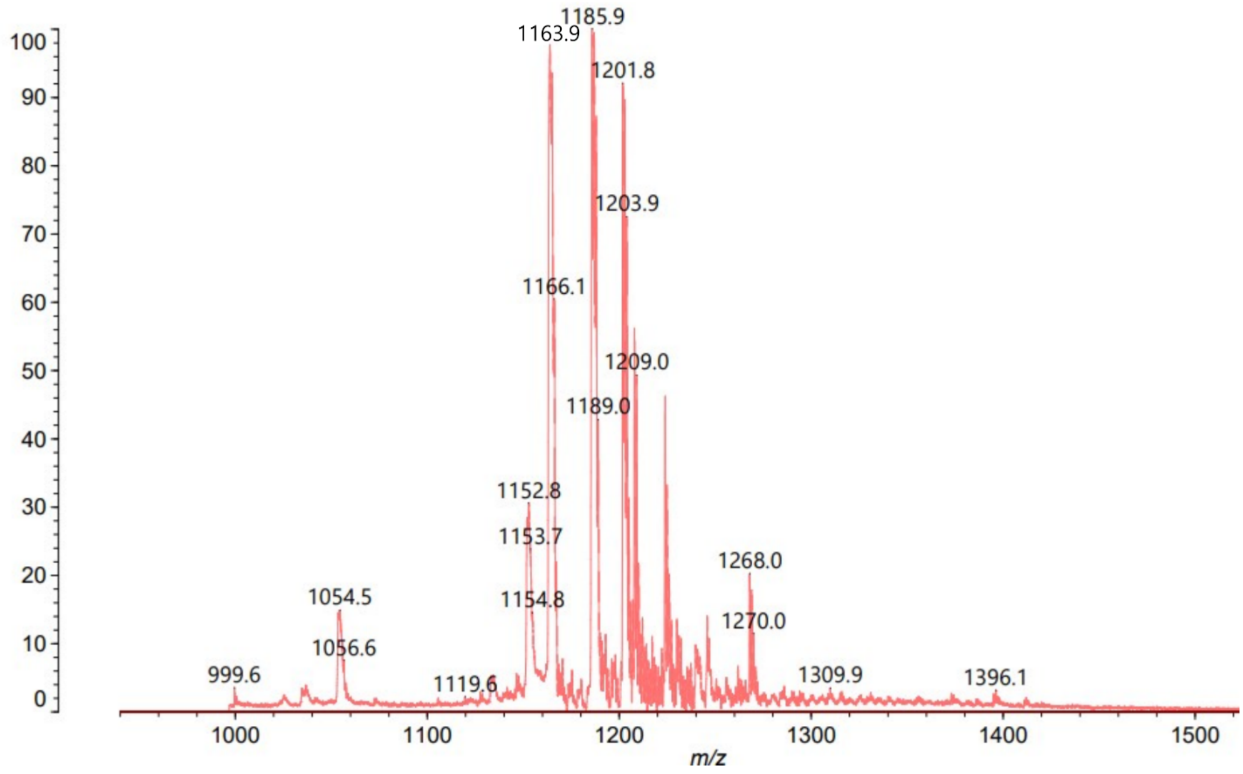

Figure S21. MALDI-TOF spectrum of Ac-FF(KE)$_2$FF-NH$_2$ (L5)

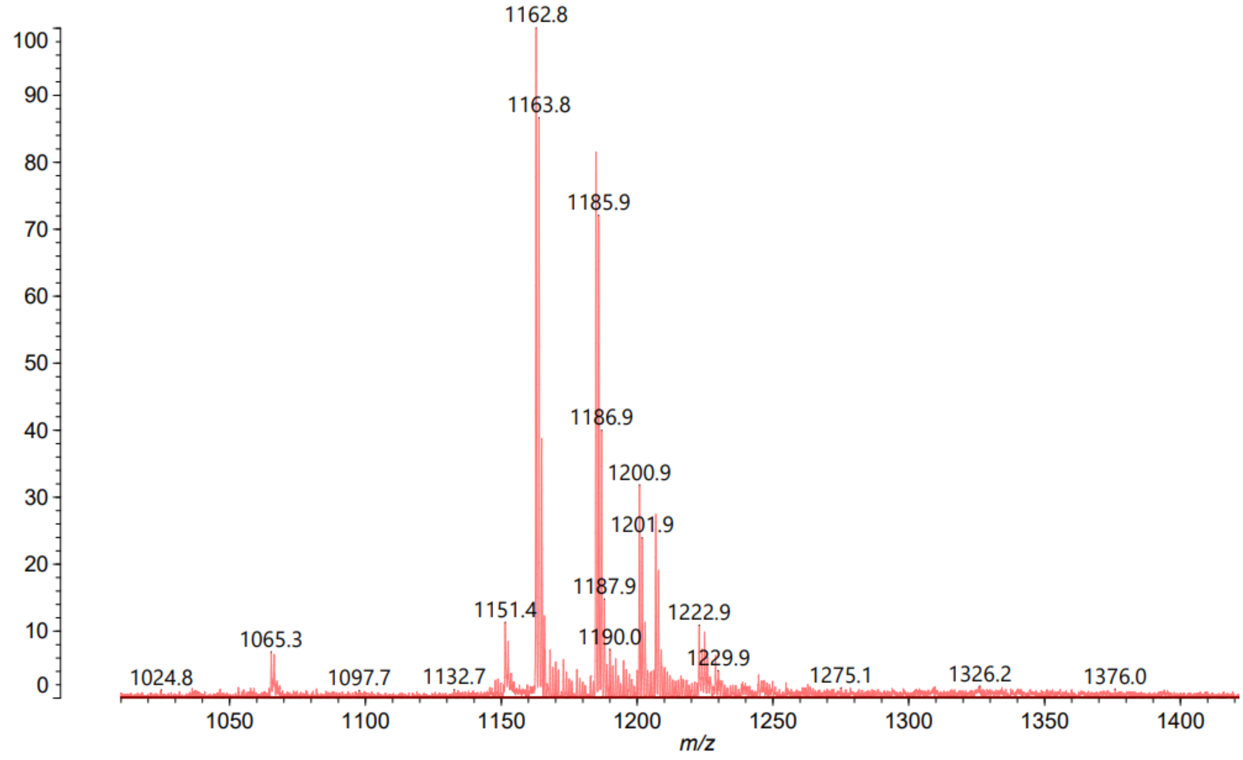

Figure S22. MALDI-TOF spectrum of Ac-(fkfe)$_2$-NH$_2$ (D1)

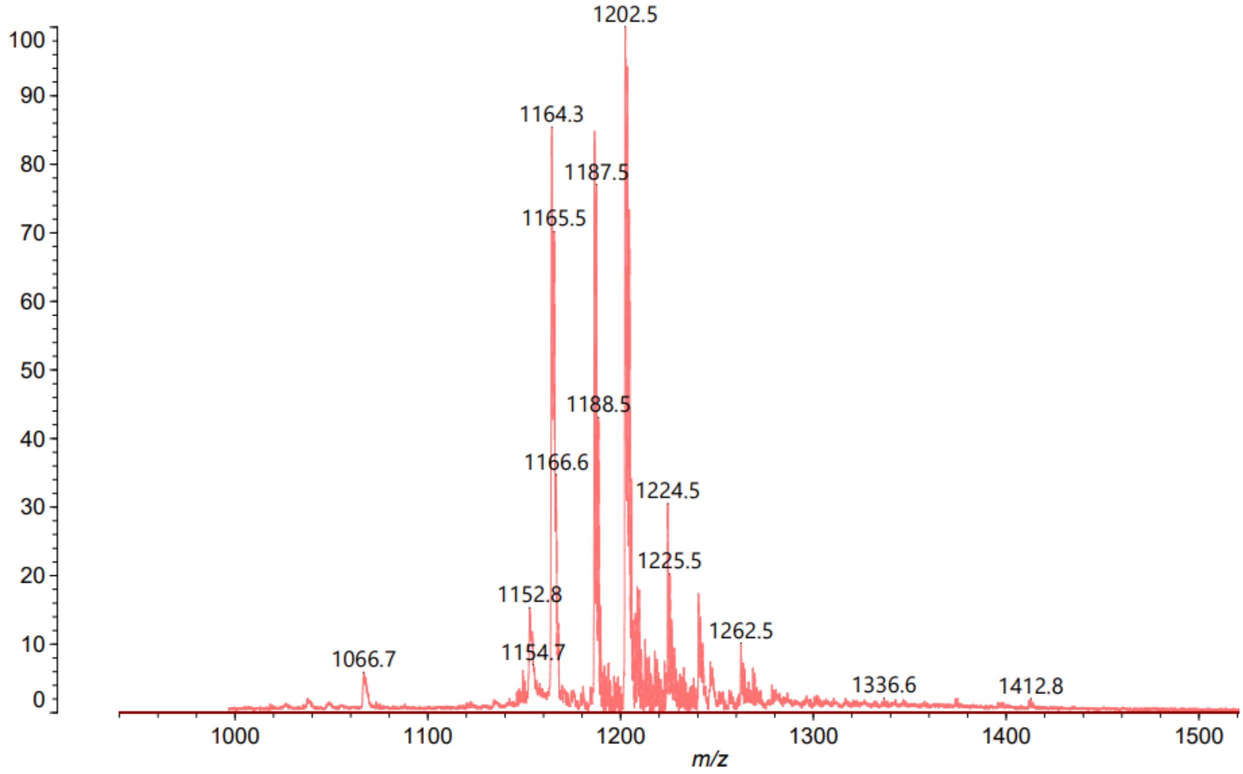

Figure S23. MALDI-TOF spectrum of Ac-(fk)$_2$(fe)$_2$-NH$_2$ (D2)

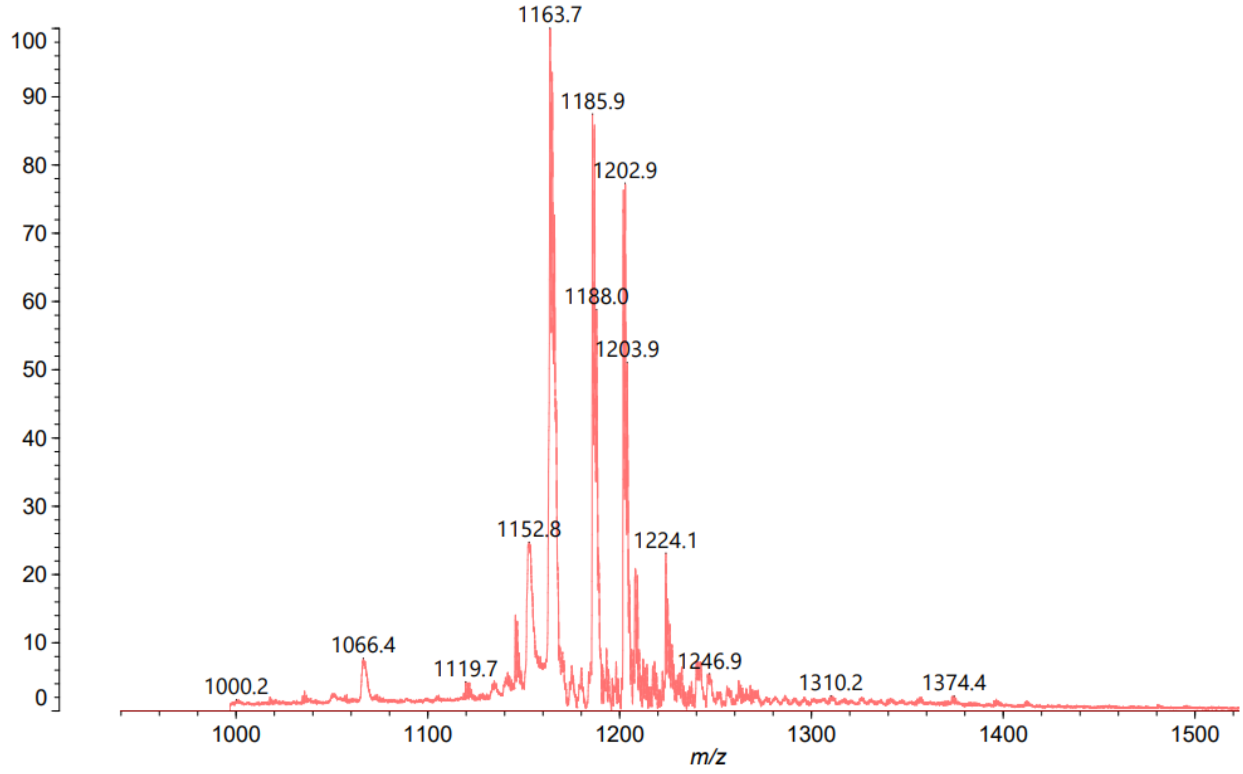

Figure S24. MALDI-TOF spectrum of Ac-ke(f)$_4$ke -NH$_2$ (D3)

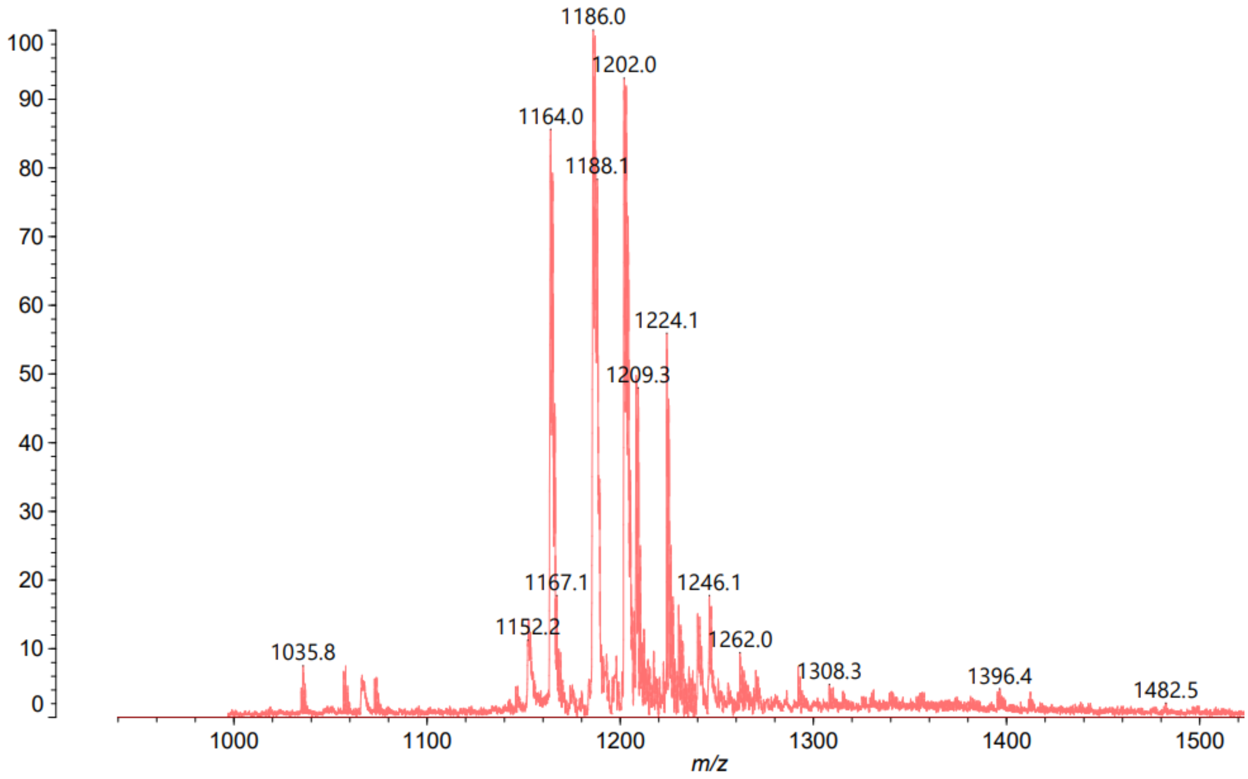

Figure S25. MALDI-TOF spectrum of Ac-$(kffe)_2$-NH_2 (D4)

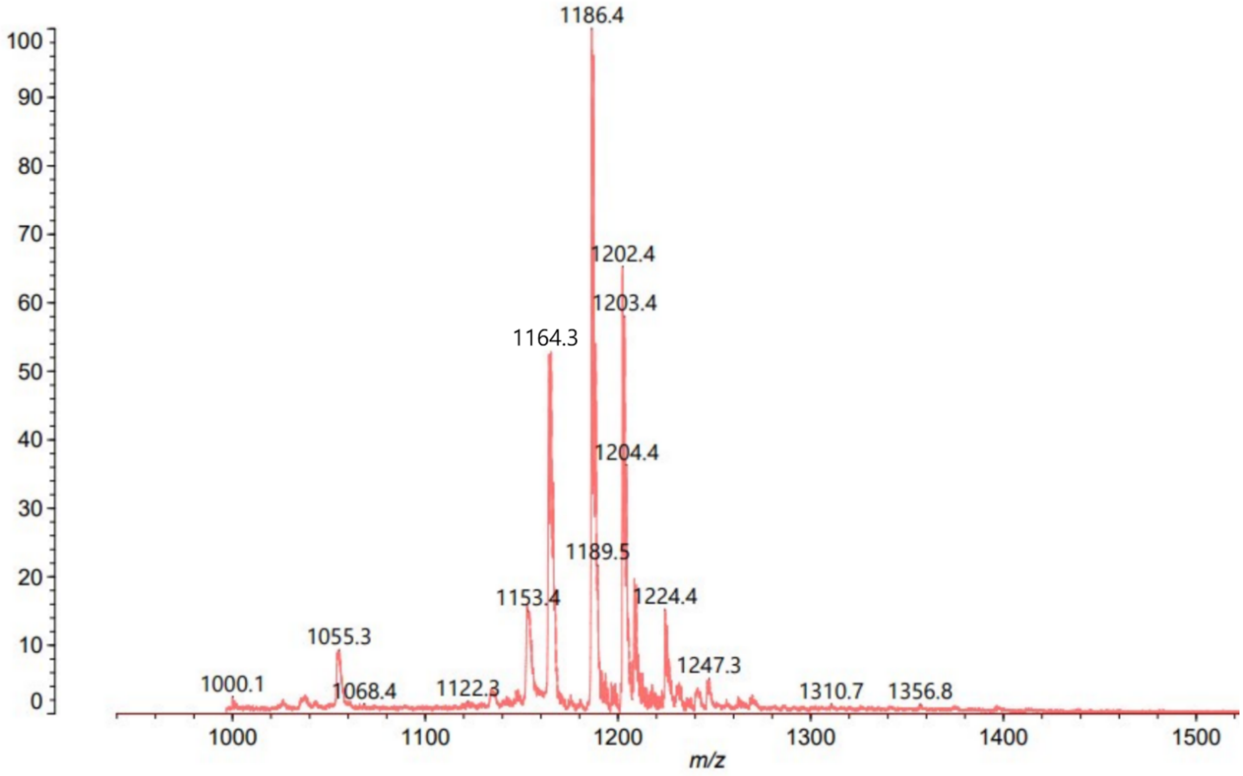

Figure S26. MALDI-TOF spectrum of Ac-ff$(ke)_2$ff-NH_2 (D5)

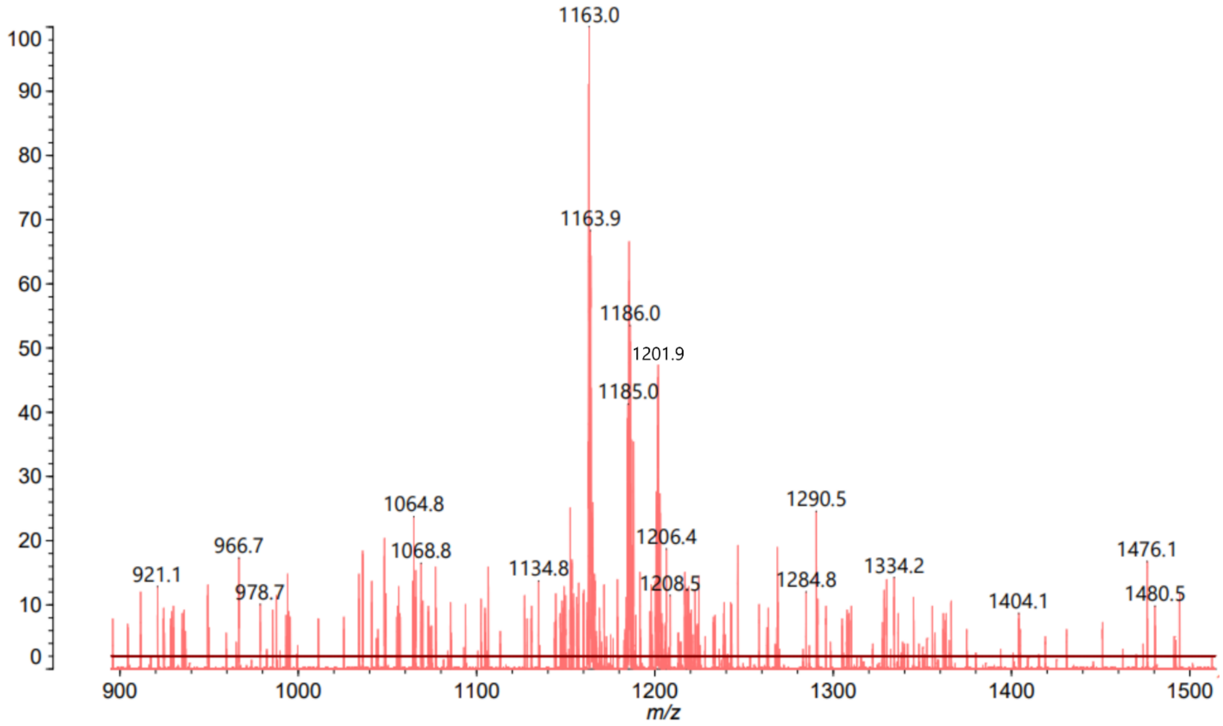

Figure S27. MALDI-TOF spectrum of Ac-F*KFEFKFE-NH_2 (L1X)

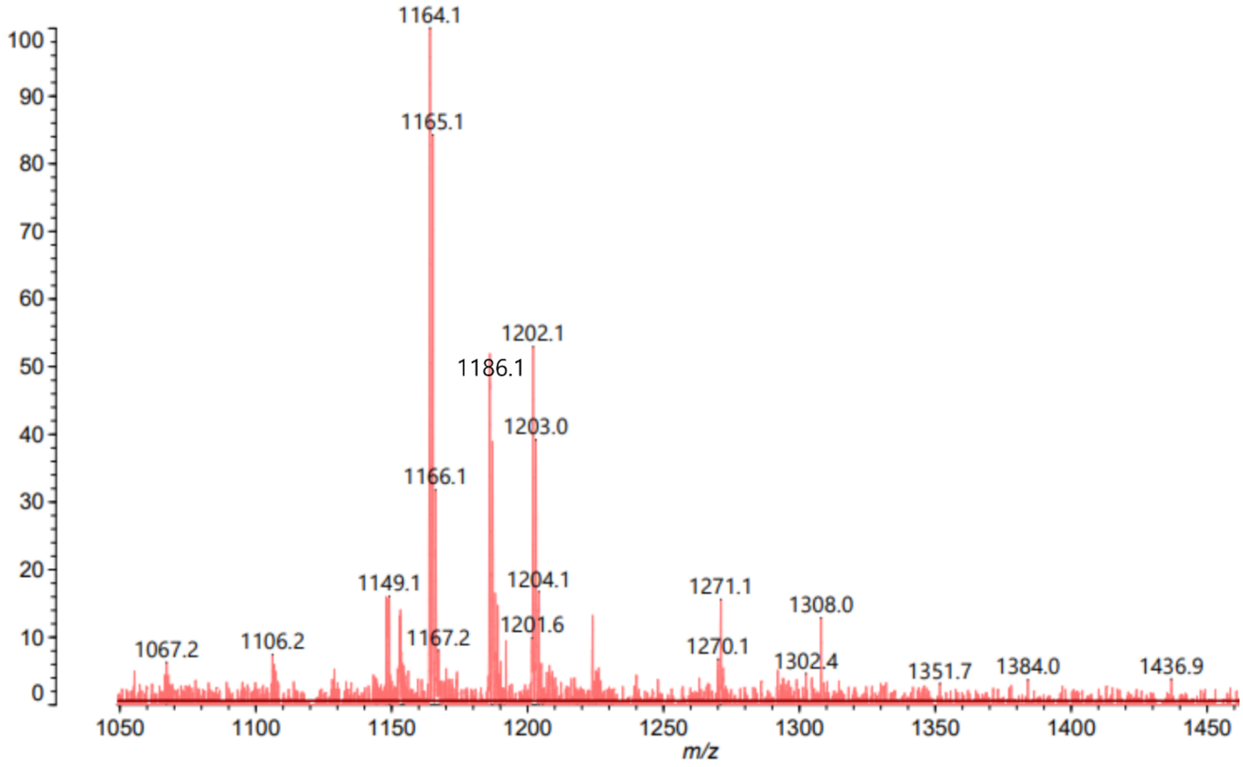

Figure S28. MALDI-TOF spectrum of Ac-F*KFEFKF*E-NH_2 (L1XX)

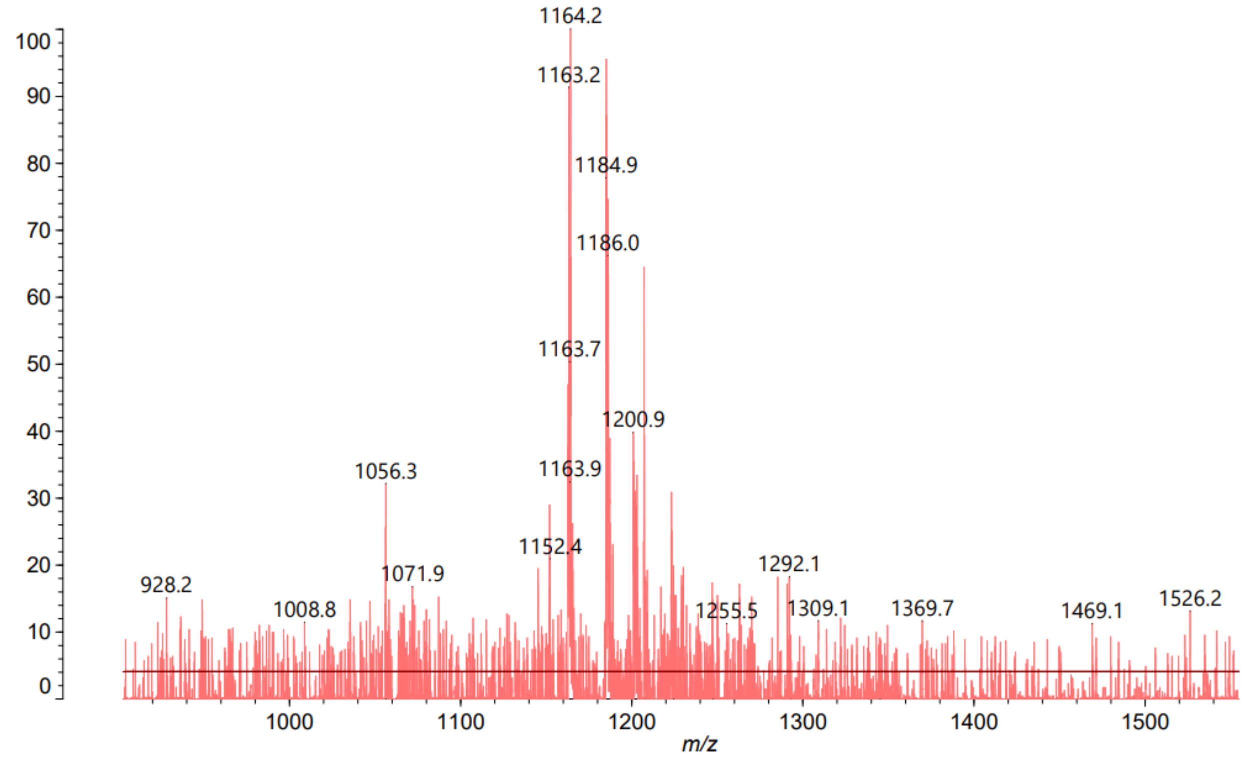

Figure S29. MALDI-TOF spectrum of Ac-F*KFKFEFE-NH_2 (L2X)

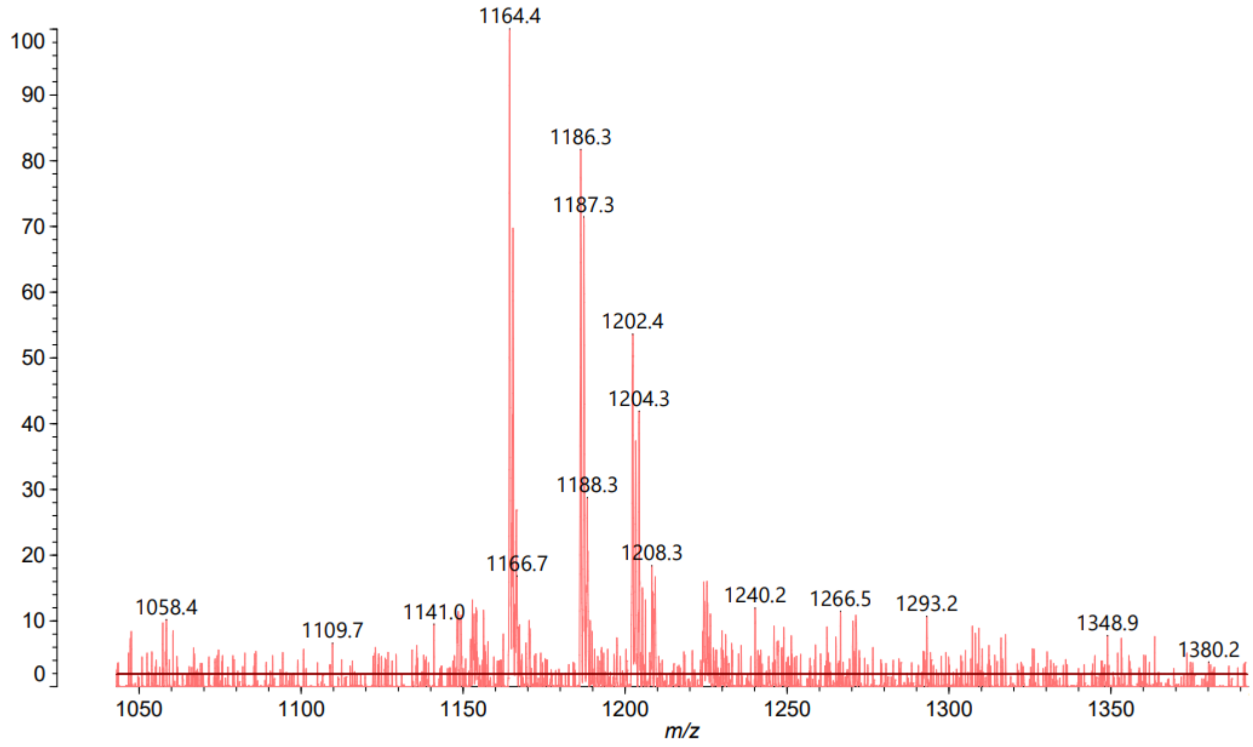

Figure S30. MALDI-TOF spectrum of Ac-F*KFKFEF*E-NH_2 (L2XX)

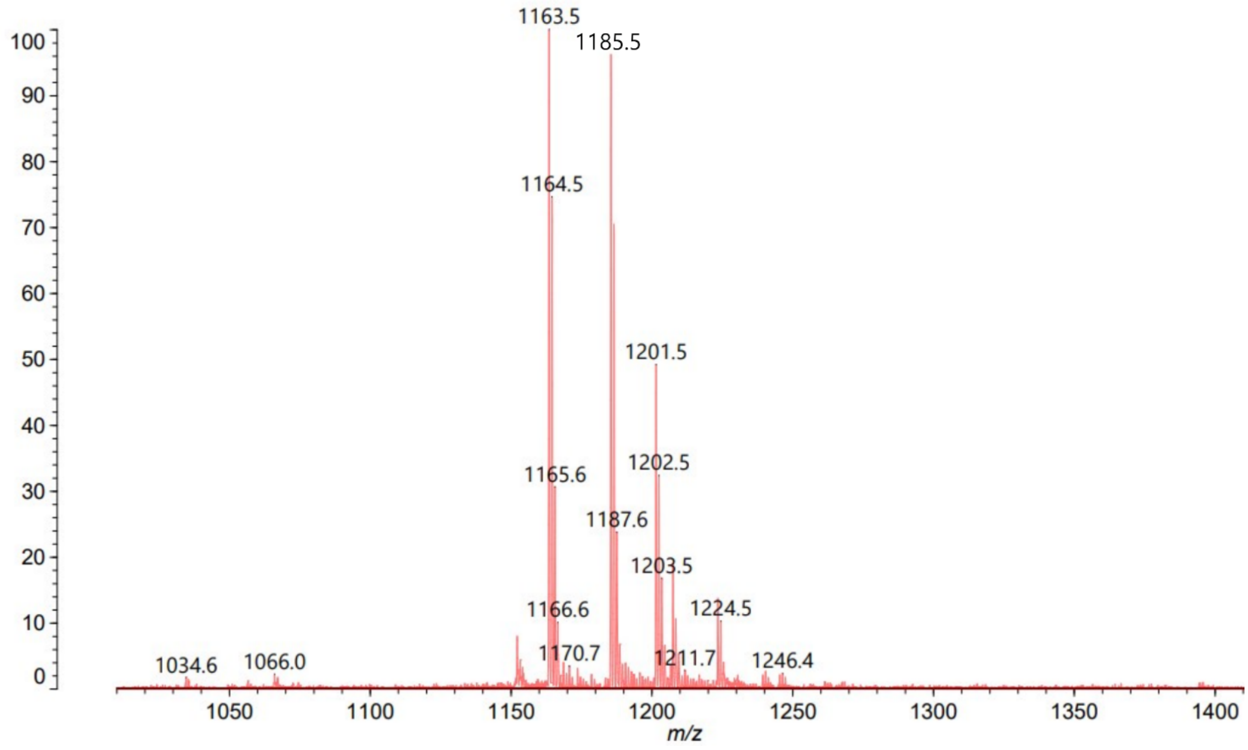

Figure S31. MALDI-TOF spectrum of Ac-KEF*FFFKE-NH_2 (L3X)

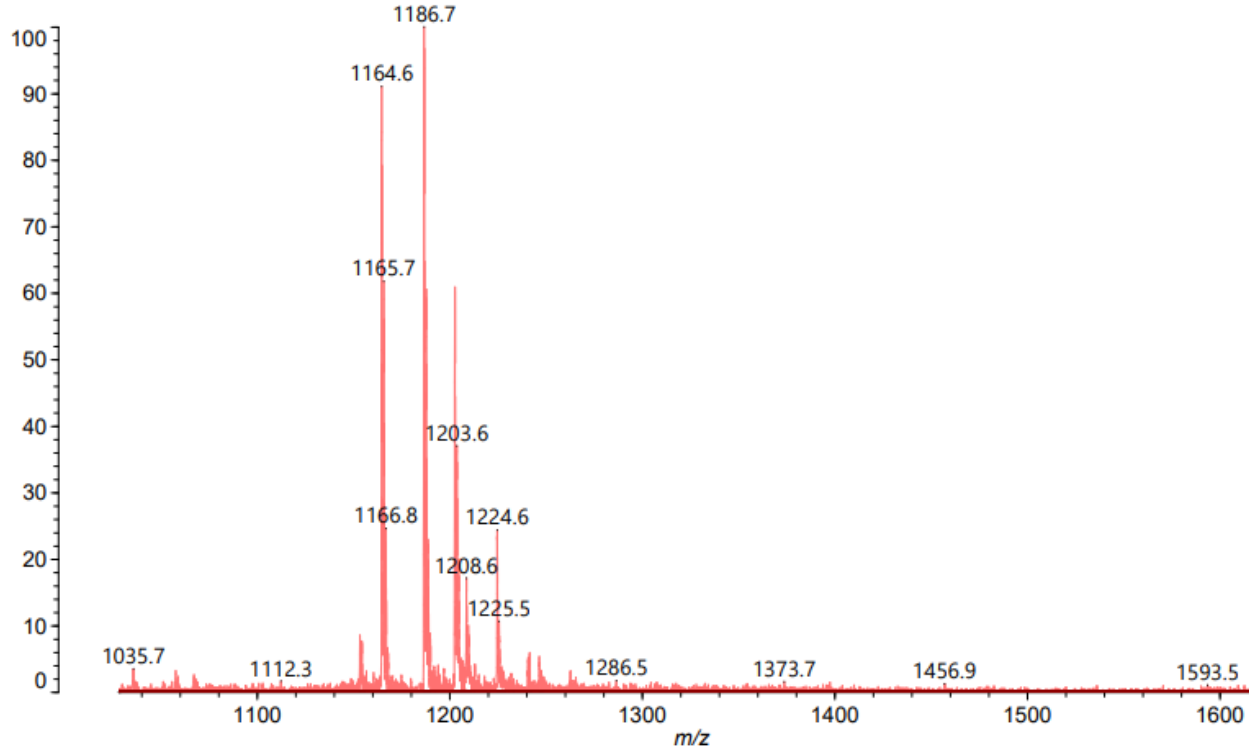

Figure S32. MALDI-TOF spectrum of Ac-KEF*FFF*KE-NH_2 (L3XX)

Table S9. Calculated and observed *m*/*z* for synthetic peptides (MALDI-TOF-MS).

	$[M+H]^+$		$[M+Na]^+$		$[M+K]^+$	
Peptide	Calculated	Observed	Calculated	Observed	Calculated	Observed
Ac-(FKFE)$_2$-NH$_2$ (L1)	1162.6	1162.7	1185.6	1184.6	1201.6	1201.9
Ac-(FK)$_2$(FE)$_2$-NH$_2$ (L2)	1162.6	1162.3	1185.6	1184.3	1201.6	1200.4
Ac-KE(F)$_4$KE-NH$_2$ (L3)	1162.6	1161.8	1185.6	1183.7	1201.6	1199.7
Ac-(KFFE)$_2$-NH$_2$ (L4)	1162.6	1163.3	1185.6	1185.2	1201.6	1201.2
Ac-FF(KE)$_2$FF-NH$_2$ (L5)	1162.6	1163.9	1185.6	1185.9	1201.6	1201.8
Ac-(fkfe)$_2$-NH$_2$ (D1)	1162.6	1162.8	1185.6	1185.9	1201.6	1200.9
Ac-(fk)$_2$(fe)$_2$-NH$_2$ (D2)	1162.6	1164.3	1185.6	1187.5	1201.6	1202.5
Ac-ke(f)$_4$ke-NH$_2$ (D3)	1162.6	1163.7	1185.6	1185.9	1201.6	1202.9
Ac-(kffe)$_2$-NH$_2$ (D4)	1162.6	1164.0	1185.6	1186.0	1201.6	1202.0
Ac-ff(ke)$_2$ke-NH$_2$ (D5)	1162.6	1164.3	1185.6	1186.4	1201.6	1202.4
Ac-F*KFEFKFE-NH$_2$ (L1X)	1163.6	1163.0	1186.6	1185.6	1202.6	1201.9
Ac-F*KFEFKF*E-NH$_2$ (L1XX)	1164.6	1164.1	1187.6	1186.1	1203.6	1202.1
Ac-F*KFKFEFE-NH$_2$ (L2X)	1163.6	1164.2	1186.6	1184.9	1202.6	1200.9
Ac-F*KFKFEF*E-NH$_2$ (L2XX)	1164.6	1164.4	1187.6	1186.3	1203.6	1202.4
Ac-KEF*FFFKE-NH$_2$ (L3X)	1163.6	1163.5	1186.6	1185.5	1202.6	1201.5
Ac-KEF*FFF*KE-NH$_2$ (L3XX)	1164.6	1164.6	1187.6	1186.7	1203.6	1202.6

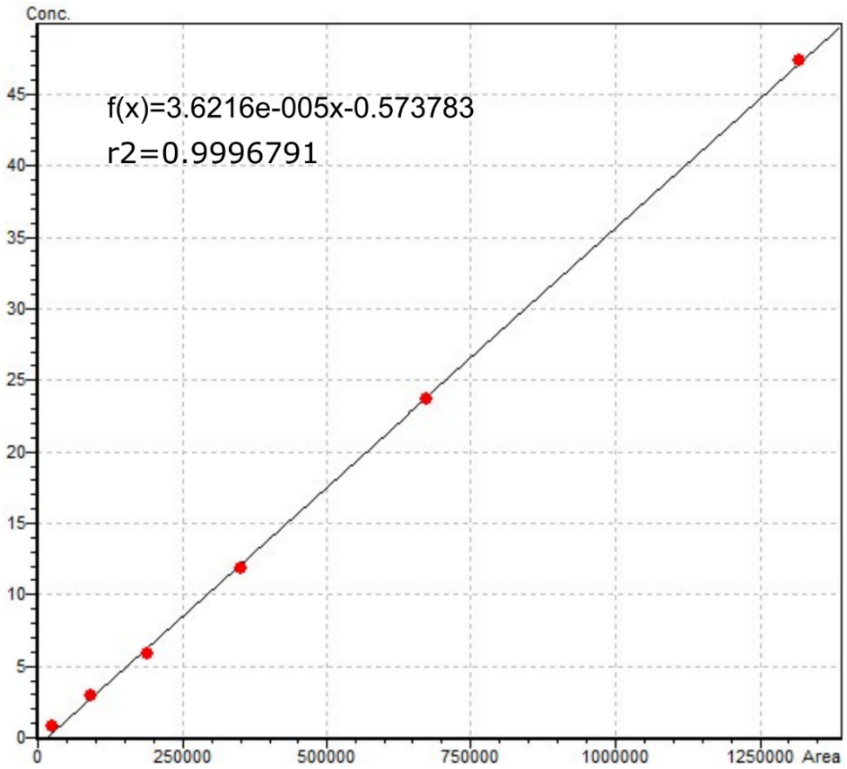

Figure S33. HPLC calibration curve (215 nm) for Ac-(FKFE)$_2$-NH$_2$ (L1)

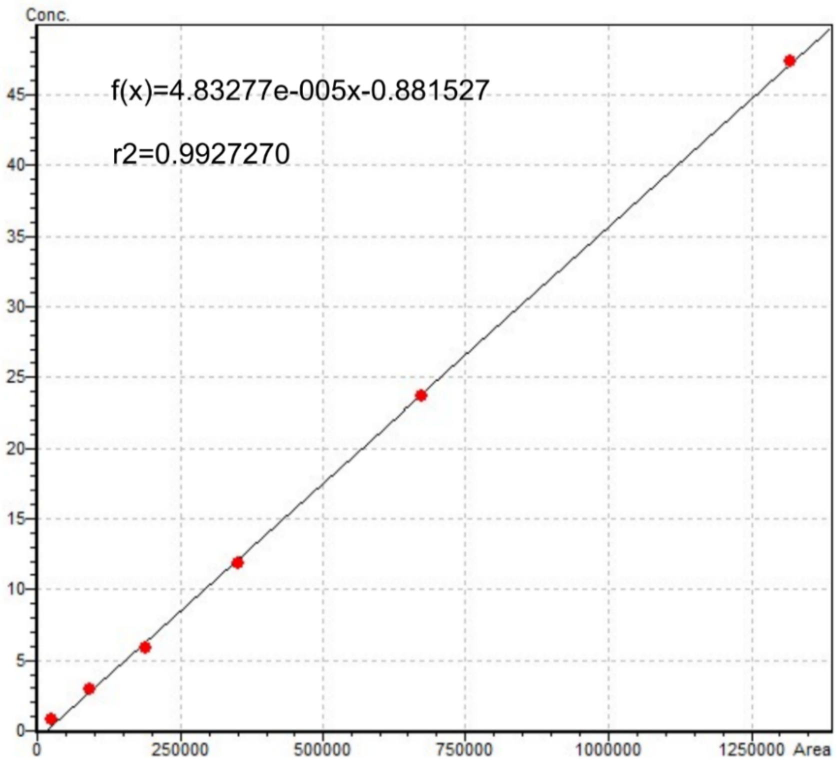

Figure S34. HPLC calibration curve (215 nm) for Ac-$(FK)_2(FE)_2$-NH_2 (L2)

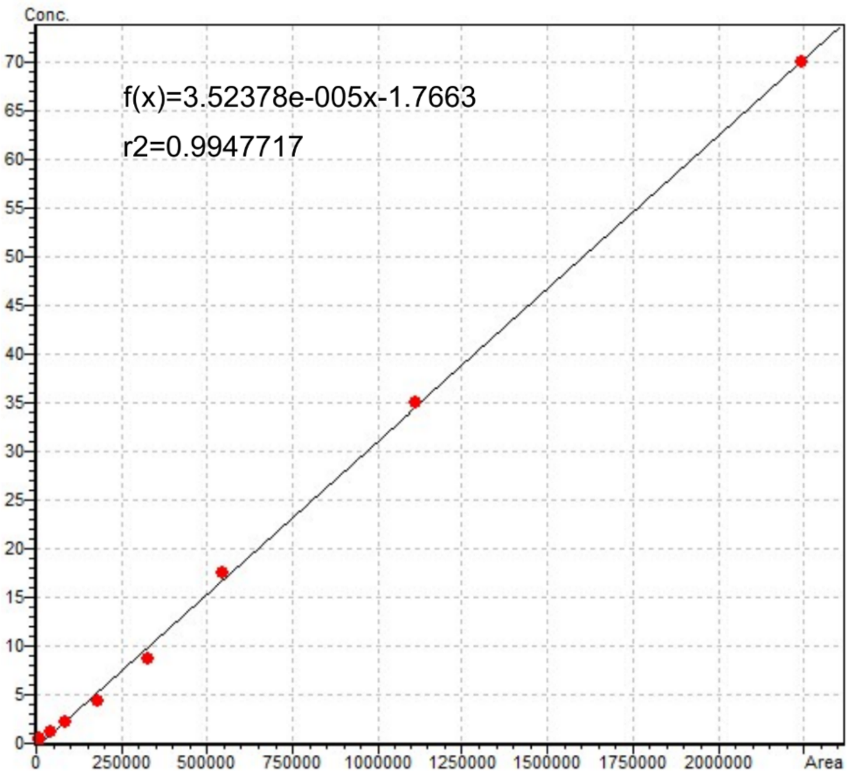

Figure S35. HPLC calibration curve (215 nm) for Ac-KE$(F)_4$KE-NH_2 (L3)

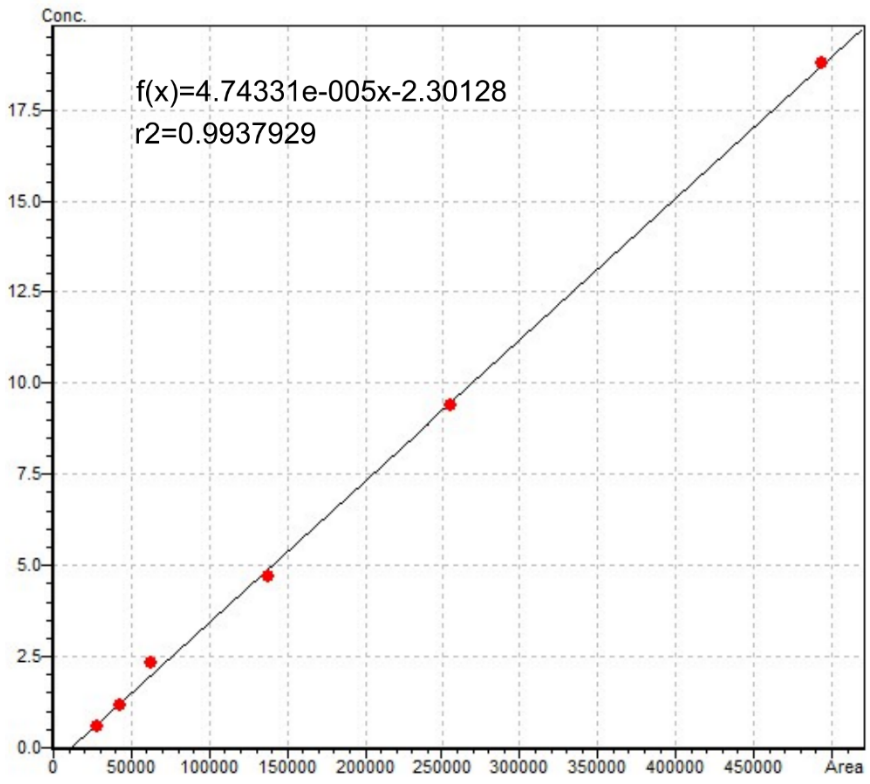

Figure S36. HPLC calibration curve (215 nm) for Ac-$(KFFE)_2$-NH_2 (L4)

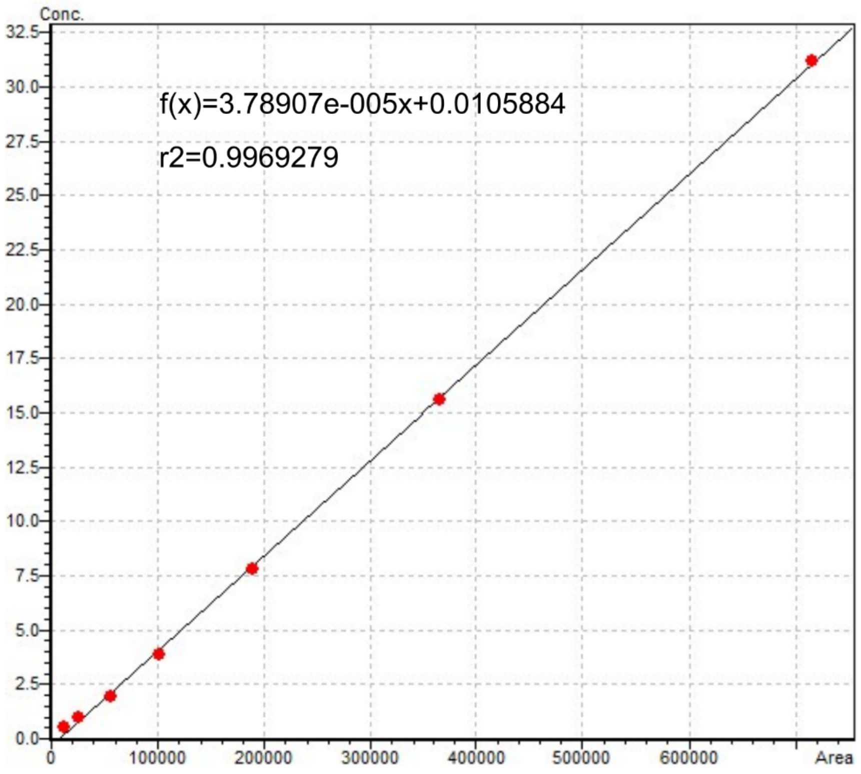

Figure S37. HPLC calibration curve (215 nm) for Ac-FF$(KE)_2$KE-NH_2 (L5)

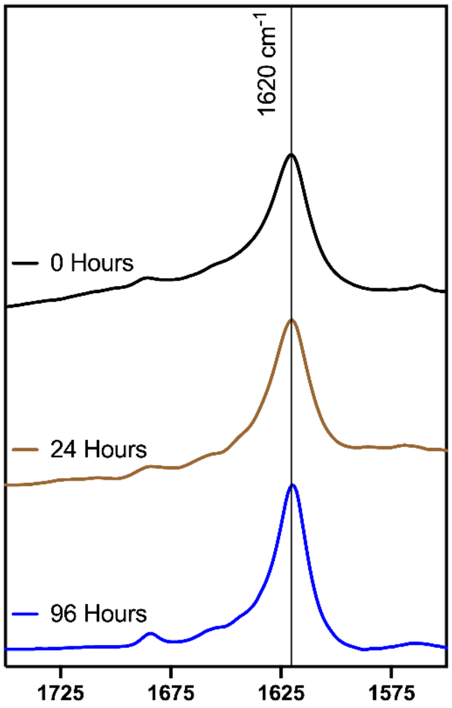

Figure S38. FTIR spectra of self-assembled **L1** (1 mM) at 0, 24, and 96 hours after assembly

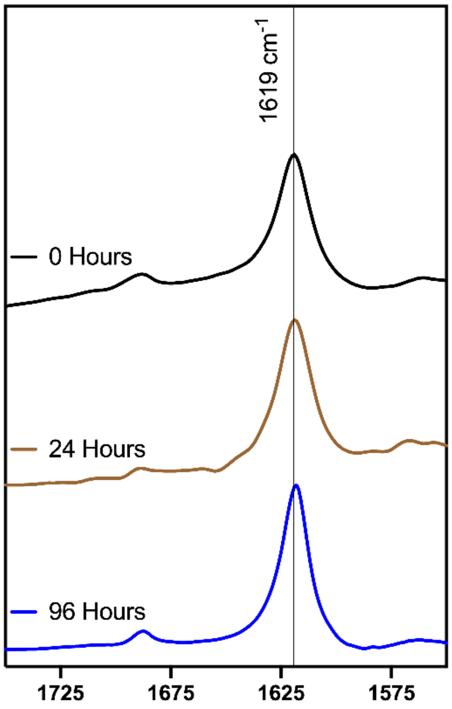

Figure S39. FTIR of coassembled **L1/D1** (1 mM) at 0, 24, and 96 hours after assembly

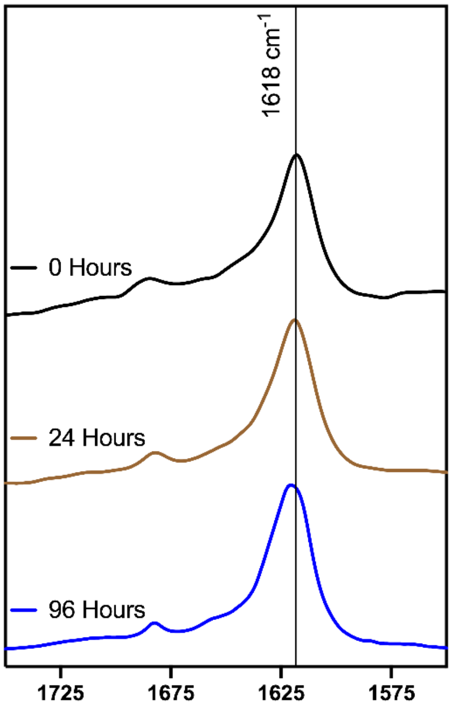

Figure S40. FTIR spectra of self-assembled **L2** (1 mM) at 0, 24, and 96 hours after assembly

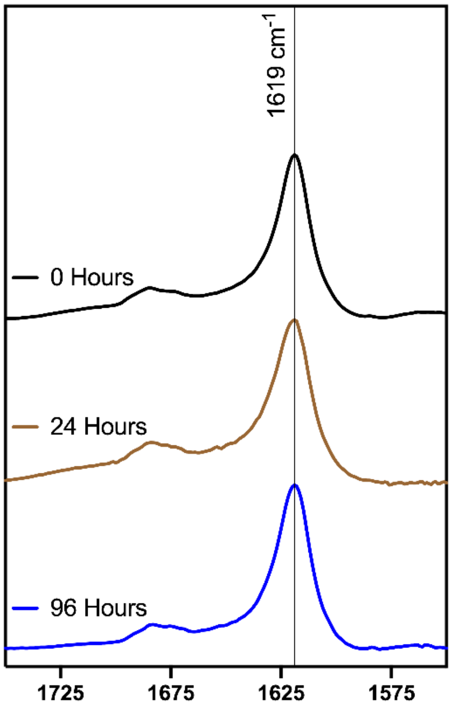

Figure S41. FTIR spectra of coassembled **L2/D2** (1 mM) at 0, 24, and 96 hours after assembly

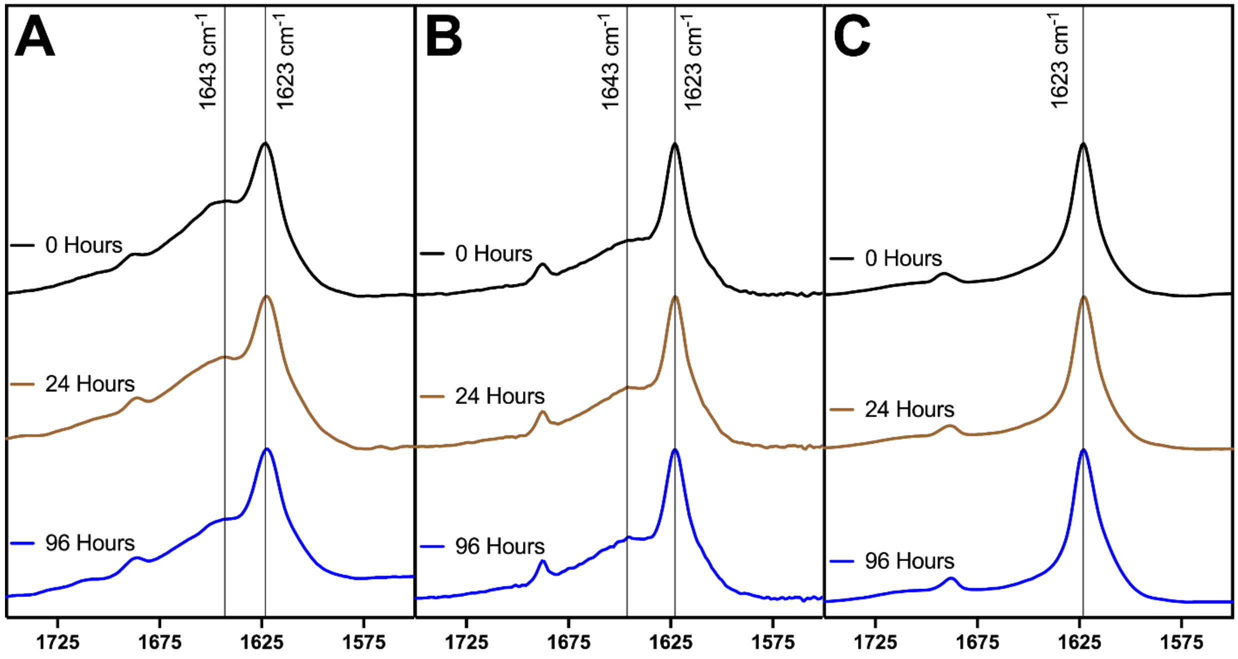

Figure 42. FTIR spectra of self-assembled **L3** at 0, 24, and 96 hours after assembly**.** (A) **L3** 1 mM, (B) **L3** 2 mM, (C) **L3** 4 mM

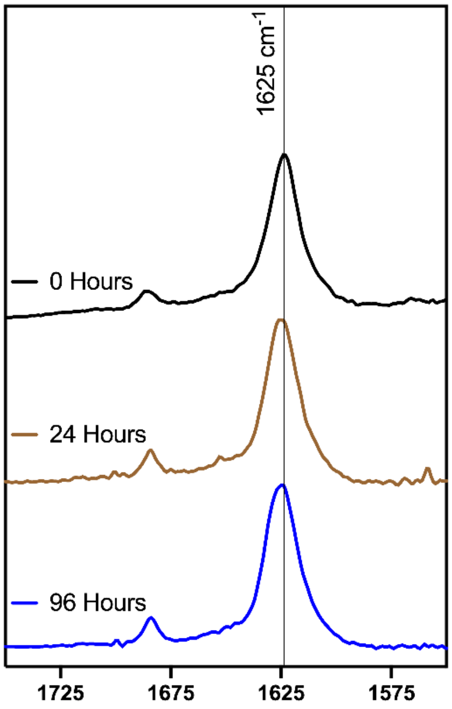

Figure 43. FTIR spectra of coassembled **L3/D3** (1 mM) at 0, 24, and 96 hours after assembly

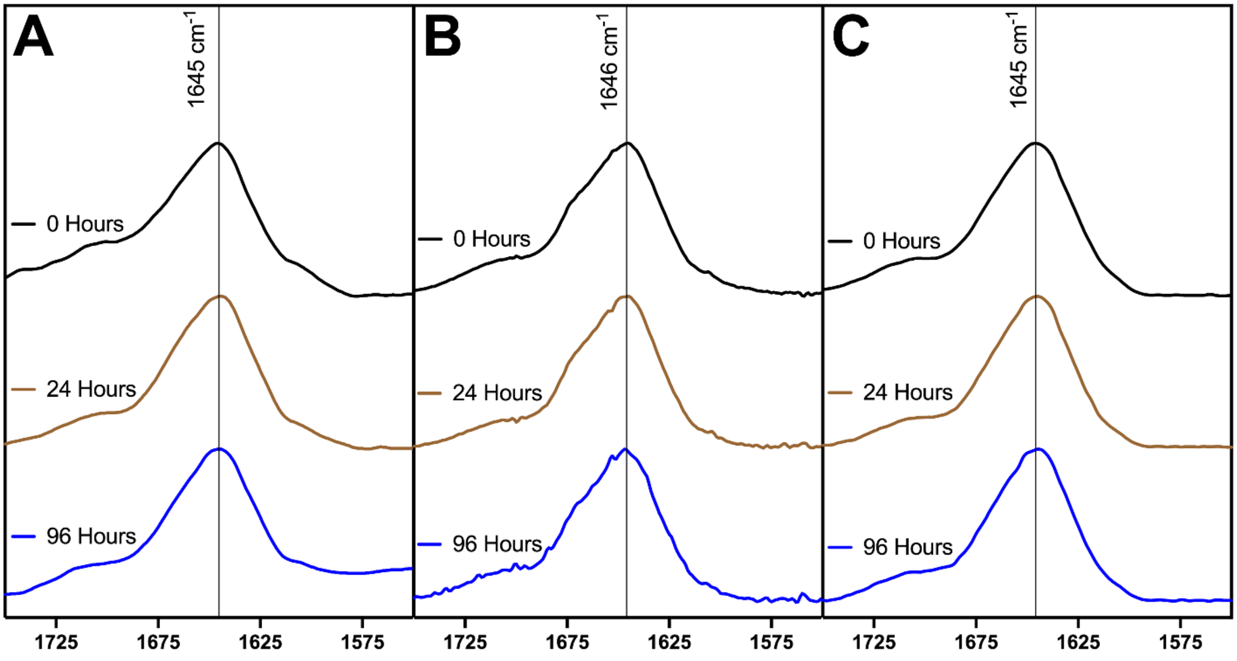

Figure 44. FTIR spectra of self-assembled **L4** at 0, 24, and 96 hours after assembly**.** (A) **L4** 1 mM, (B) **L4** 2 mM, (C) **L4** 4 mM

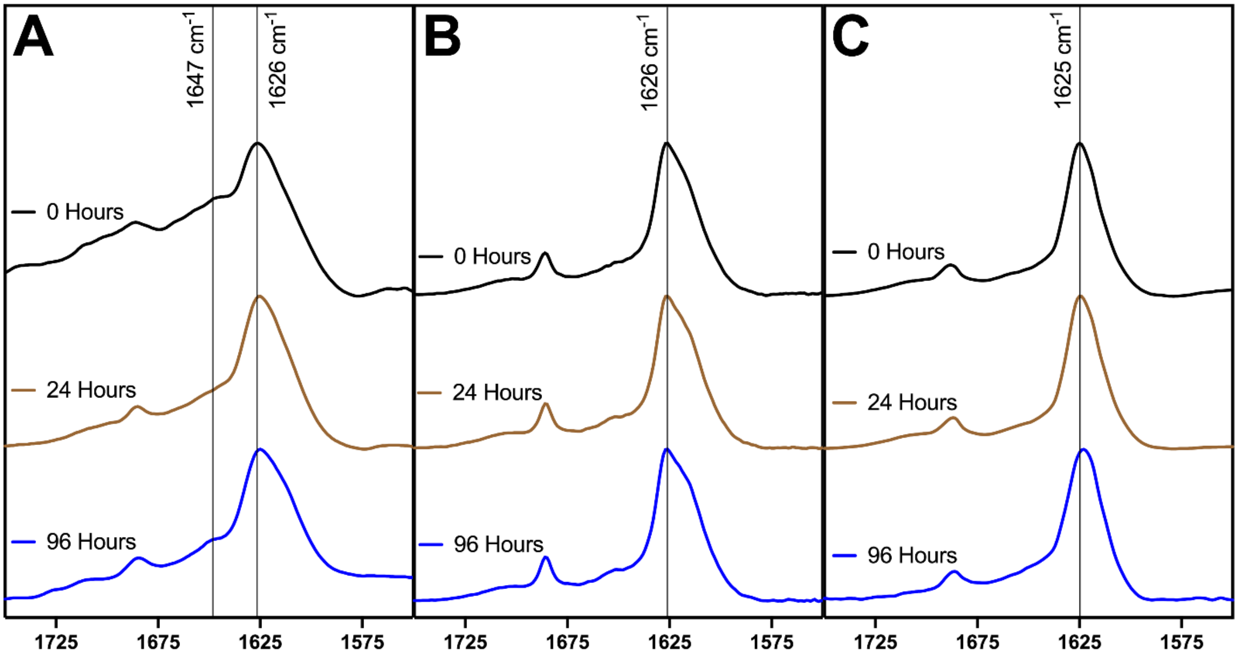

Figure 45. FTIR spectra of coassembled **L4/D4** at 0, 24, and 96 hours after assembly**.** (A) **L4/D4** 1 mM, (B) **L4/D4** 2 mM, (C) **L4/D4** 4 mM

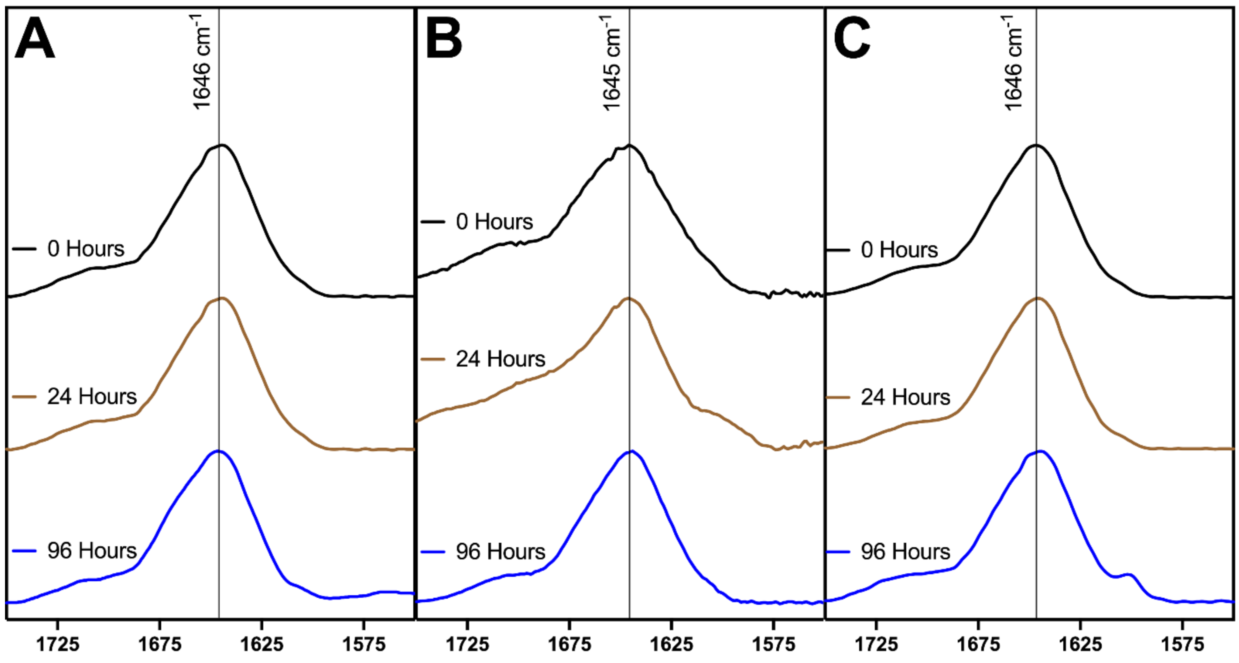

Figure 46. FTIR spectra of self-assembled **L5** at 0, 24, and 96 hours after assembly**.** (A) **L5** 1 mM, (B) **L5** 2 mM, (C) **L5** 4 mM

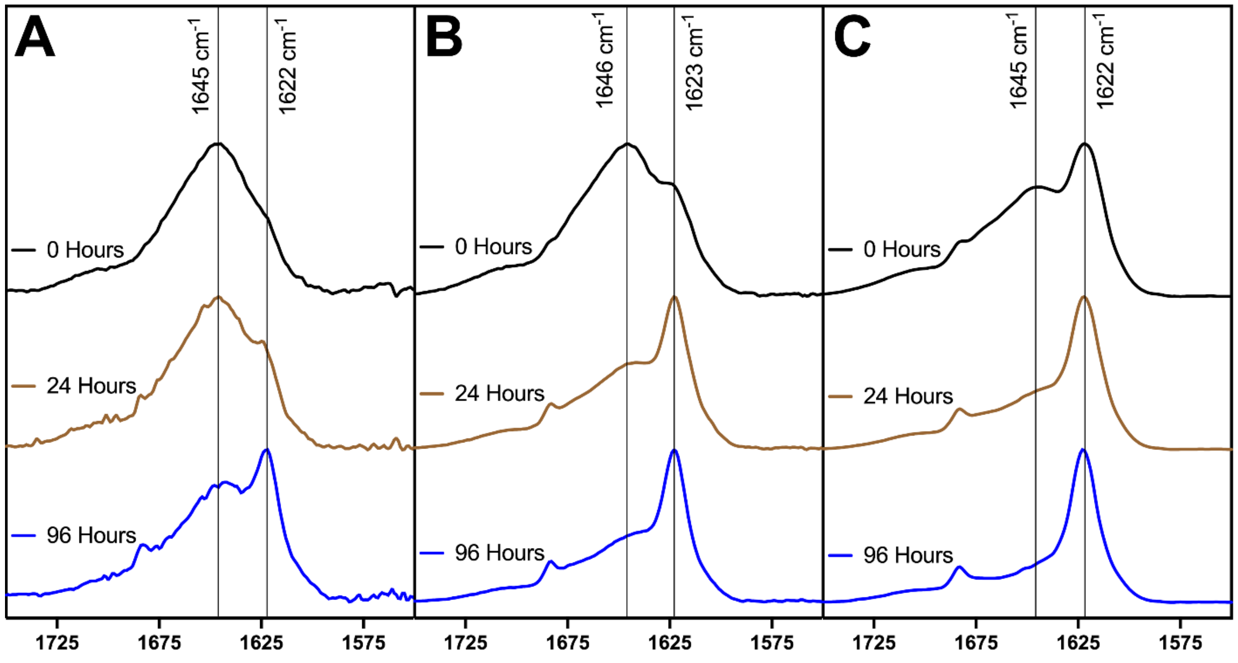

Figure 47. FTIR spectra of coassembled **L5/D5** at 0, 24, and 96 hours after assembly**.** (A) **L5/D5** 1 mM, (B) **L5/D5** 2 mM, (C) **L5/D5** 4 mM

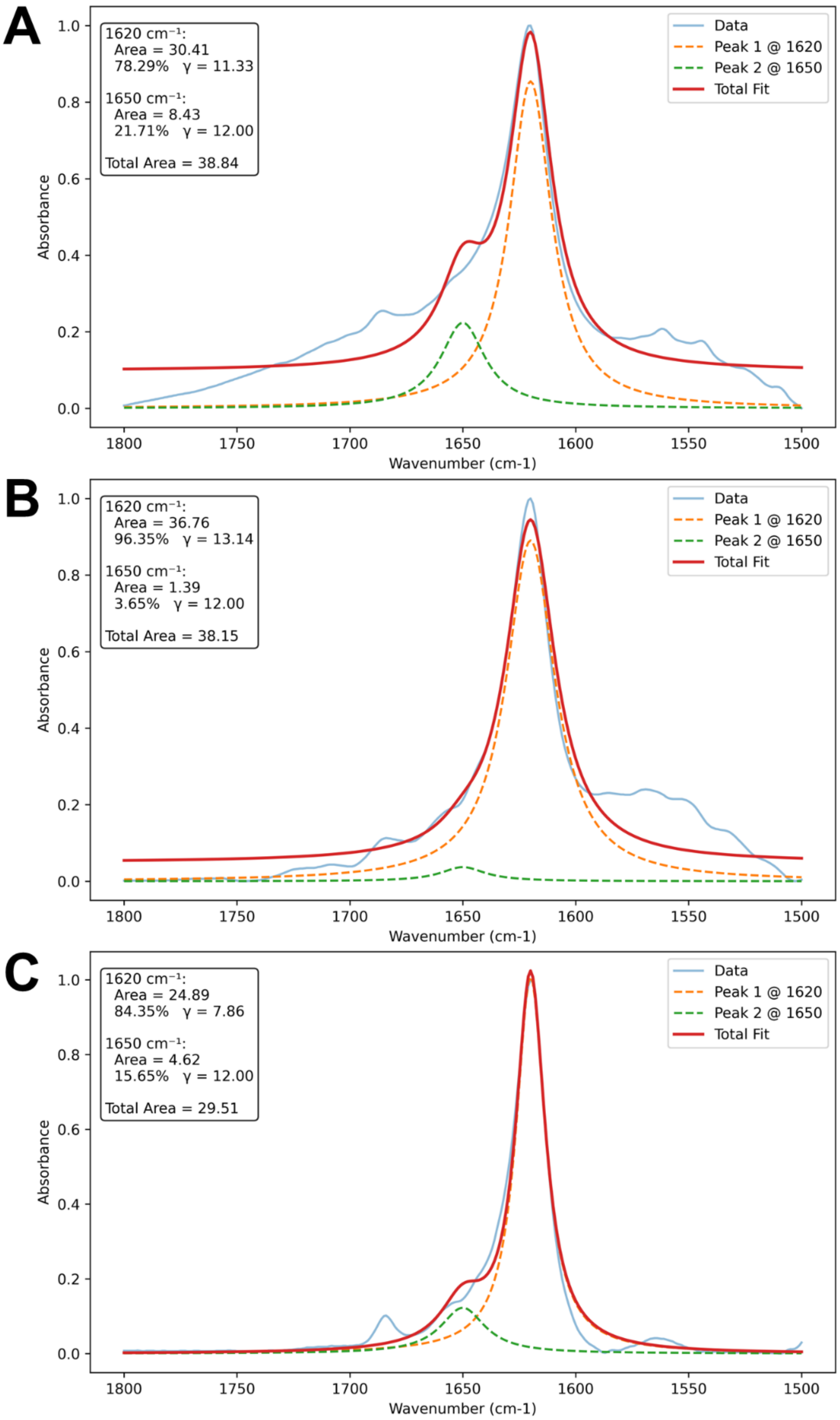

Figure S48. FTIR spectrum of **L1** 1 mM (solid blue) with an applied Lorentzian function to quantify the structural contributions of β-sheet at 1620 cm^{-1} (dotted orange) and random aggregate at 1650 cm^{-1}(dotted green), and a combined fit of the two peaks (solid red). (A) **L1** 1 mM 0 hours, (B) **L1** 1 mM 24 hours, (C) **L1** 1 mM 96 hours.

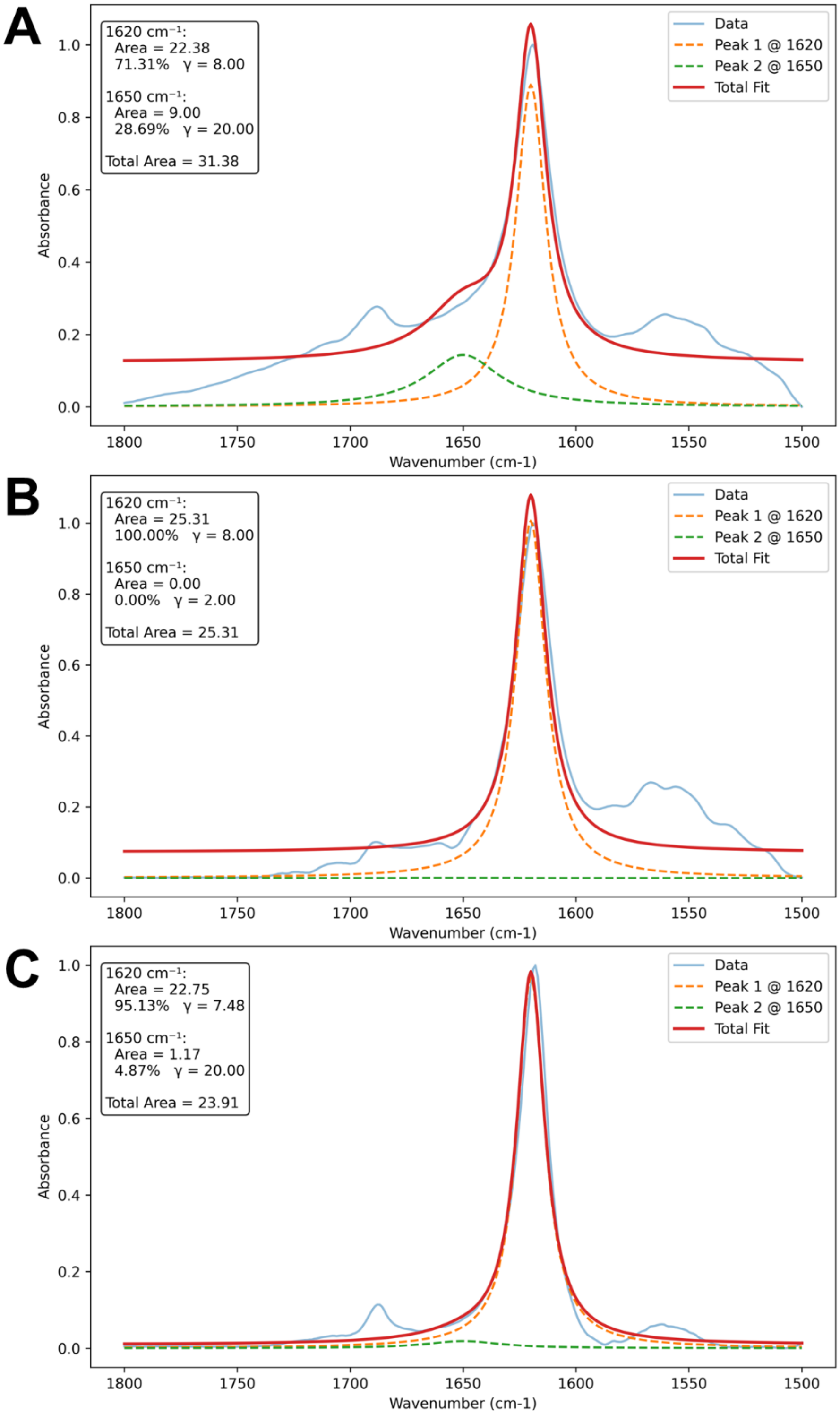

Figure S49. FTIR spectrum of **L1/D1** 1 mM (solid blue) with an applied Lorentzian function to quantify the structural contributions of β-sheet at 1620 cm^{-1} (dotted orange) and random aggregate at 1650 cm^{-1}(dotted green), and a combined fit of the two peaks (solid red). (A) **L1/D1** 1 mM 0 hours, (B) **L1/D1** 1 mM 24 hours, (C) **L1/D1** 1 mM 96 hours.

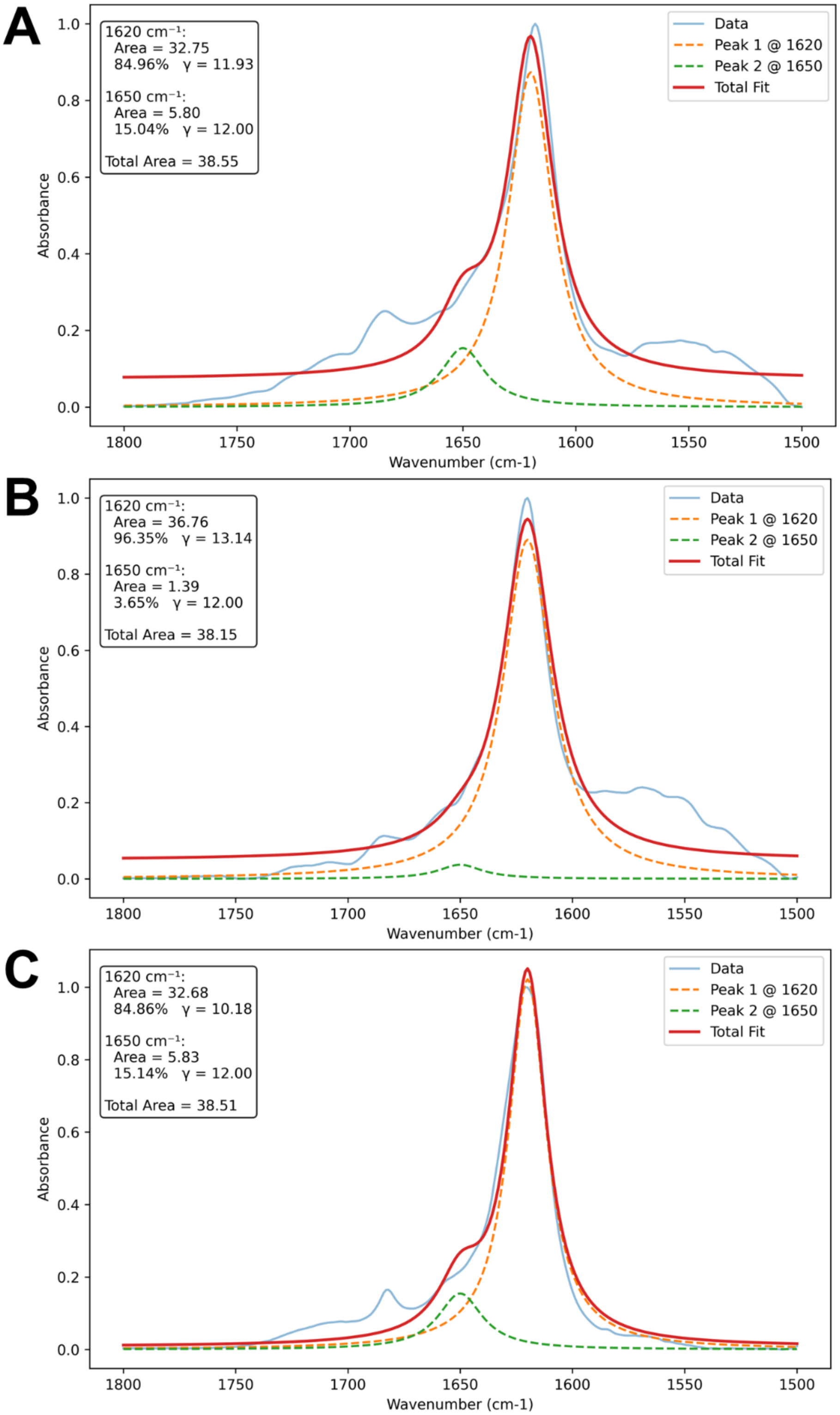

Figure S50. FTIR spectrum of **L2** 1 mM (solid blue) with an applied Lorentzian function to quantify the structural contributions of β-sheet at 1620 cm^{-1} (dotted orange) and random aggregate at 1650 cm^{-1}(dotted green), and a combined fit of the two peaks (solid red). (A) **L2** 1 mM 0 hours, (B) **L2** 1 mM 24 hours, (C) **L2** 1 mM 96 hours.

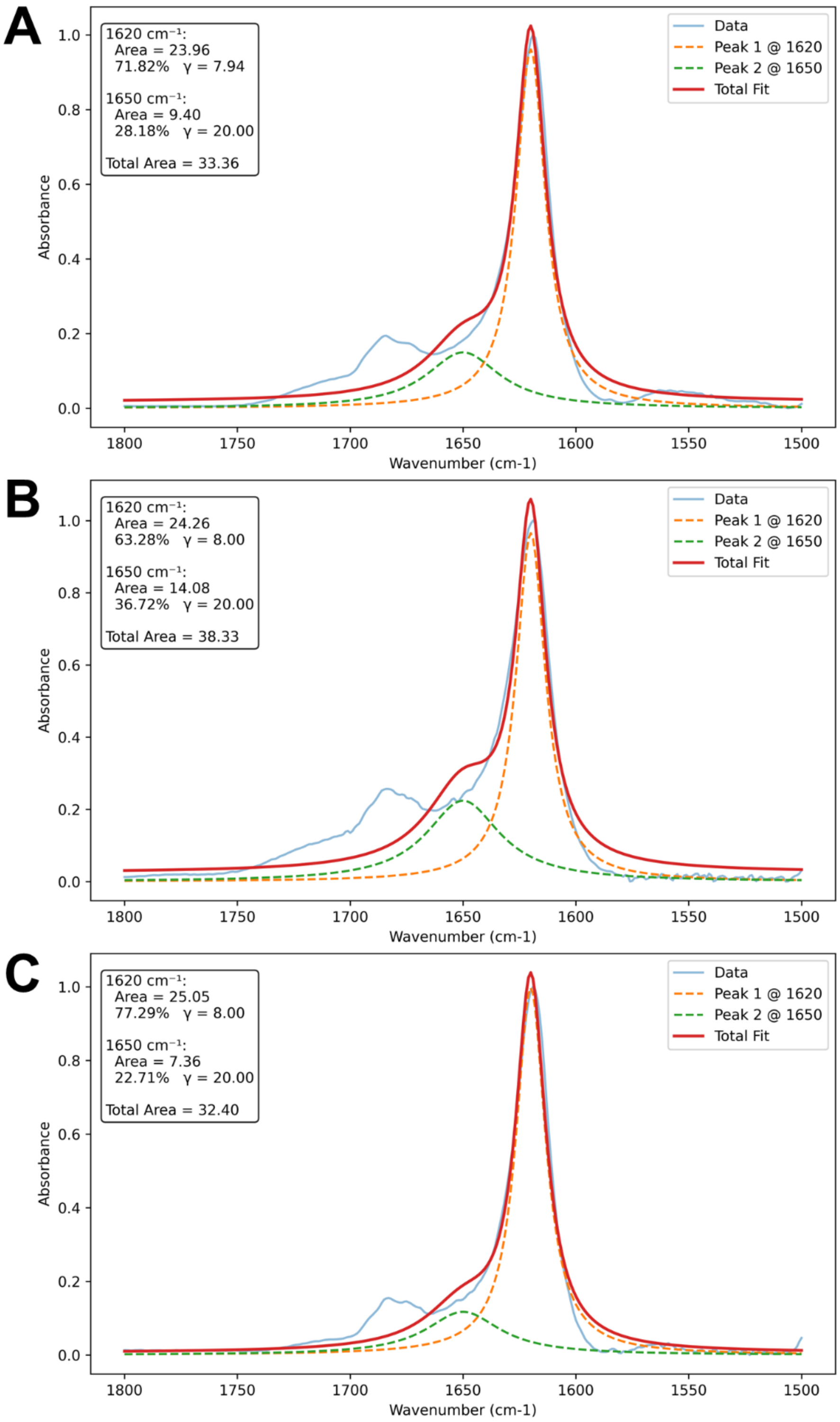

Figure S51. FTIR spectrum of **L2/D2** 1 mM (solid blue) with an applied Lorentzian function to quantify the structural contributions of β-sheet at 1620 cm^{-1} (dotted orange) and random aggregate at 1650 cm^{-1}(dotted green), and a combined fit of the two peaks (solid red). (A) **L2/D2** 1 mM 0 hours, (B) **L2/D2** 1 mM 24 hours, (C) **L2/D2** 1 mM 96 hours.

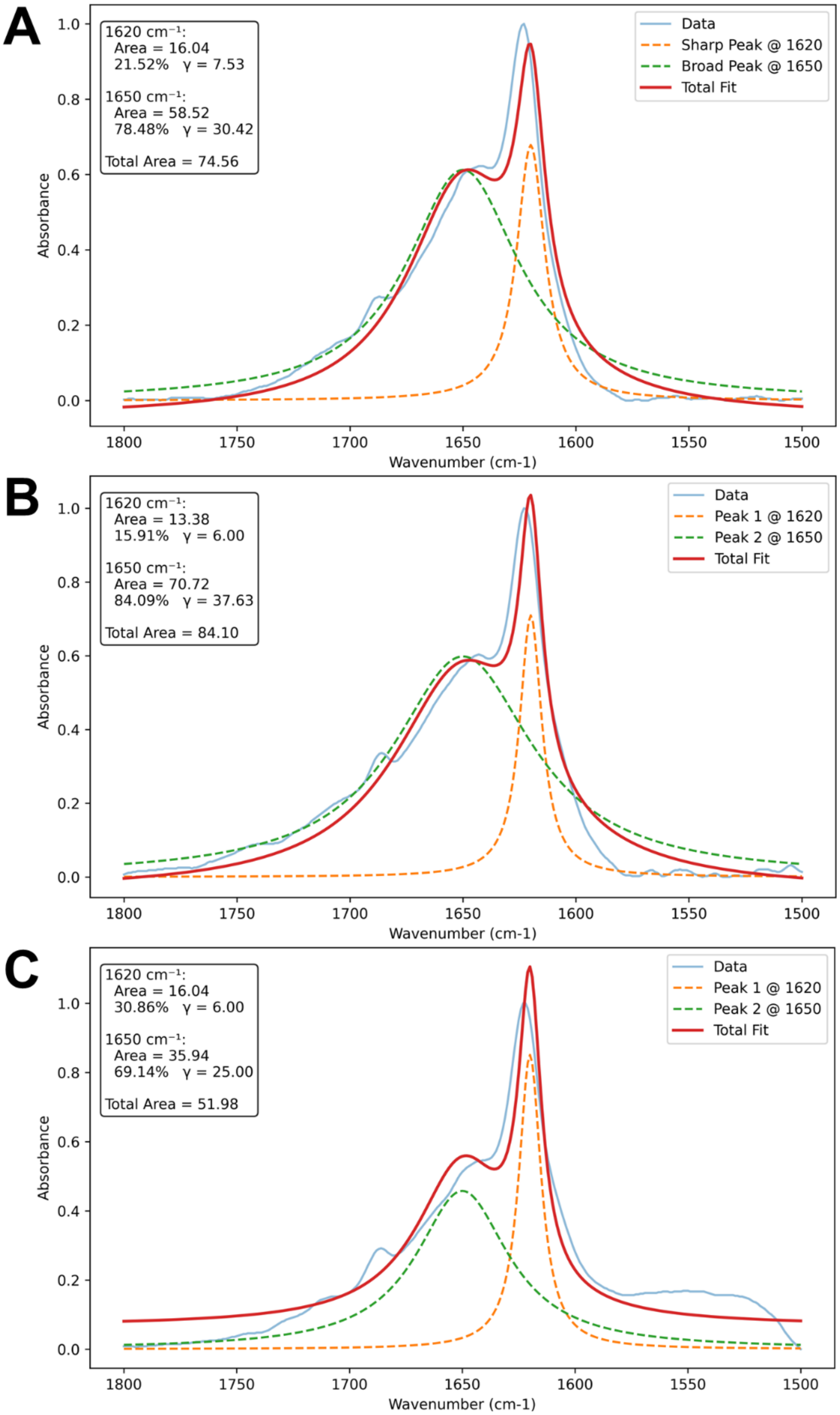

Figure S52. FTIR spectrum of **L3** 1 mM (solid blue) with an applied Lorentzian function to quantify the structural contributions of β-sheet at 1620 cm^{-1} (dotted orange) and random aggregate at 1650 cm^{-1}(dotted green), and a combined fit of the two peaks (solid red). (A) **L3** 1 mM 0 hours, (B) **L3** 1 mM 24 hours, (C) **L3** 1 mM 96 hours.

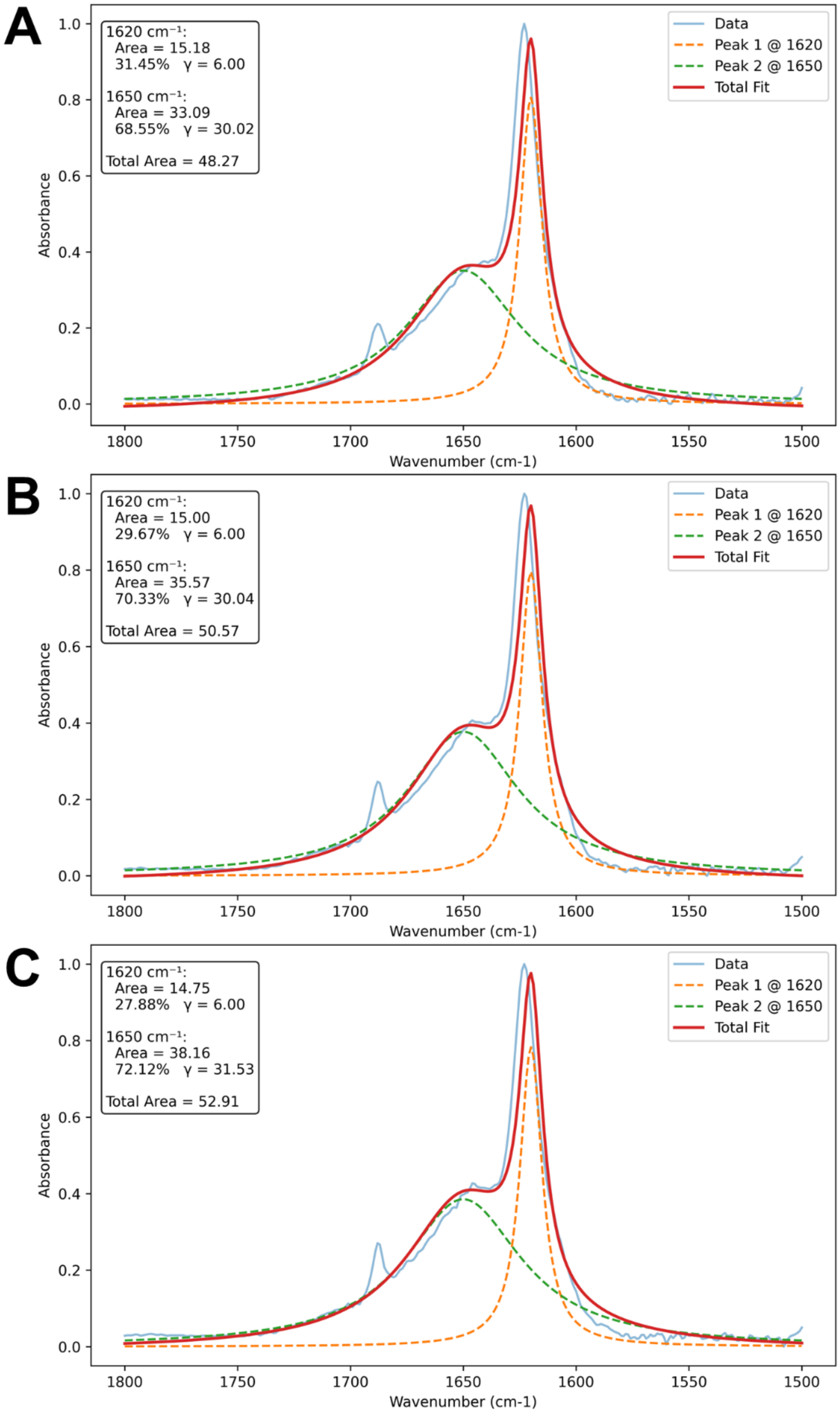

Figure S53. FTIR spectrum of **L3** 2 mM (solid blue) with an applied Lorentzian function to quantify the structural contributions of β-sheet at 1620 cm^{-1} (dotted orange) and random aggregate at 1650 cm^{-1}(dotted green), and a combined fit of the two peaks (solid red). (A) **L3** 2 mM 0 hours, (B) **L3** 2 mM 24 hours, (C) **L3** 2 mM 96 hours.

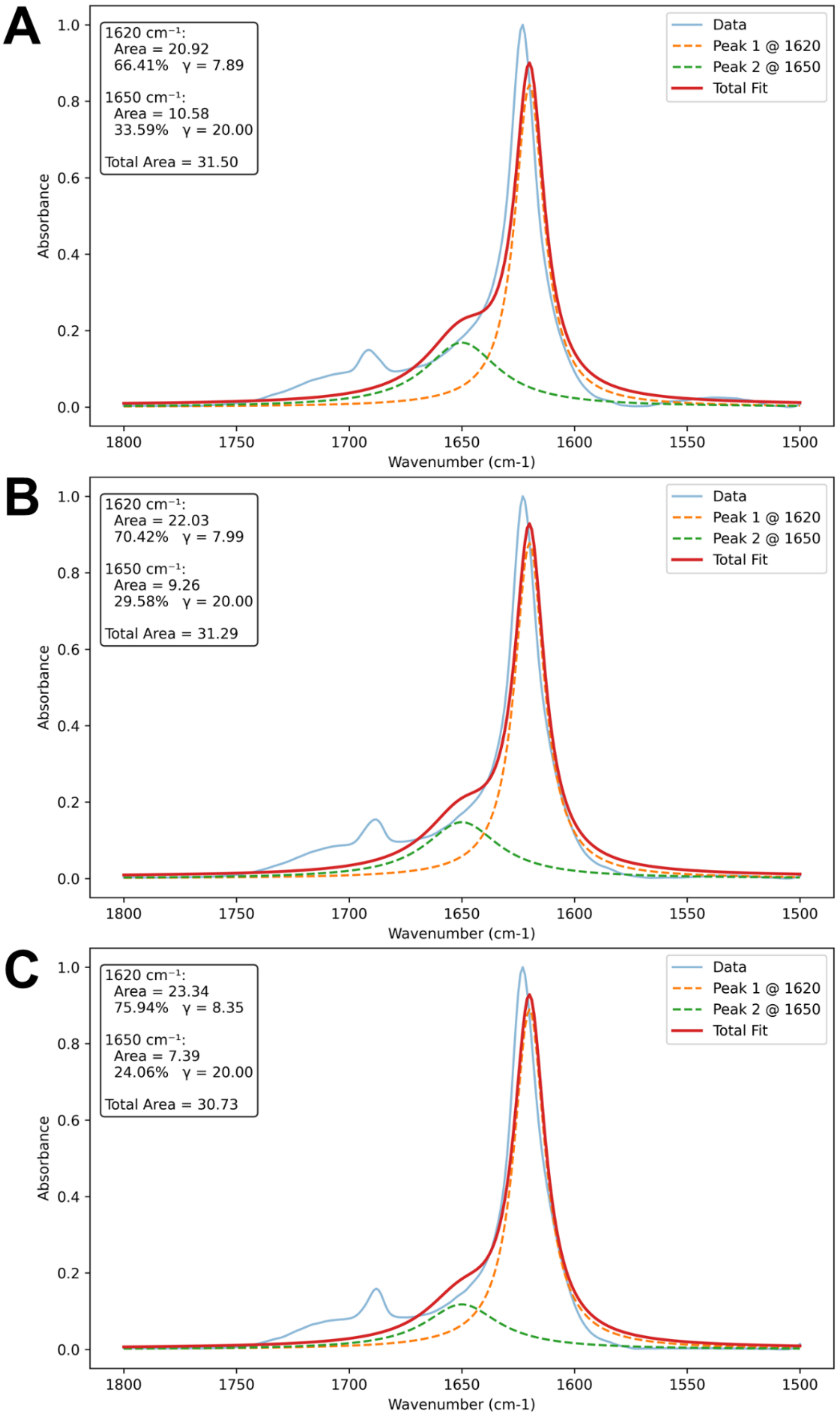

Figure S54. FTIR spectrum of **L3** 4 mM (solid blue) with an applied Lorentzian function to quantify the structural contributions of β-sheet at 1620 cm^{-1} (dotted orange) and random aggregate at 1650 cm^{-1}(dotted green), and a combined fit of the two peaks (solid red). (A) **L3** 4 mM 0 hours, (B) **L3** 4 mM 24 hours, (C) **L3** 4 mM 96 hours.

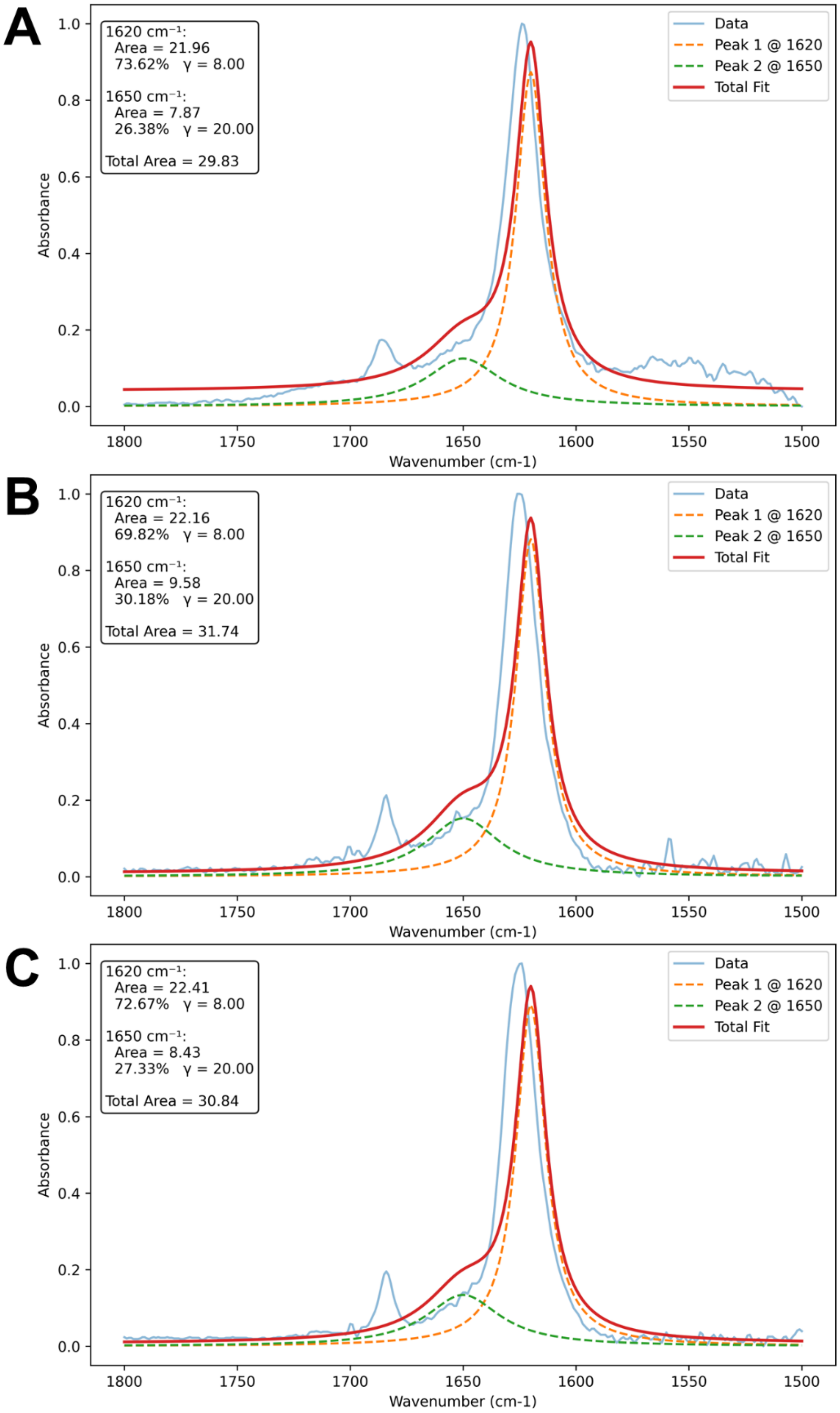

Figure S55. FTIR spectrum of **L3/D3** 1 mM (solid blue) with an applied Lorentzian function to quantify the structural contributions of β-sheet at 1620 cm^{-1} (dotted orange) and random aggregate at 1650 cm^{-1}(dotted green), and a combined fit of the two peaks (solid red). (A) **L3/D3** 1 mM 0 hours, (B) **L3/D3** 1 mM 24 hours, (C) **L3/D3** 1 mM 96 hours.

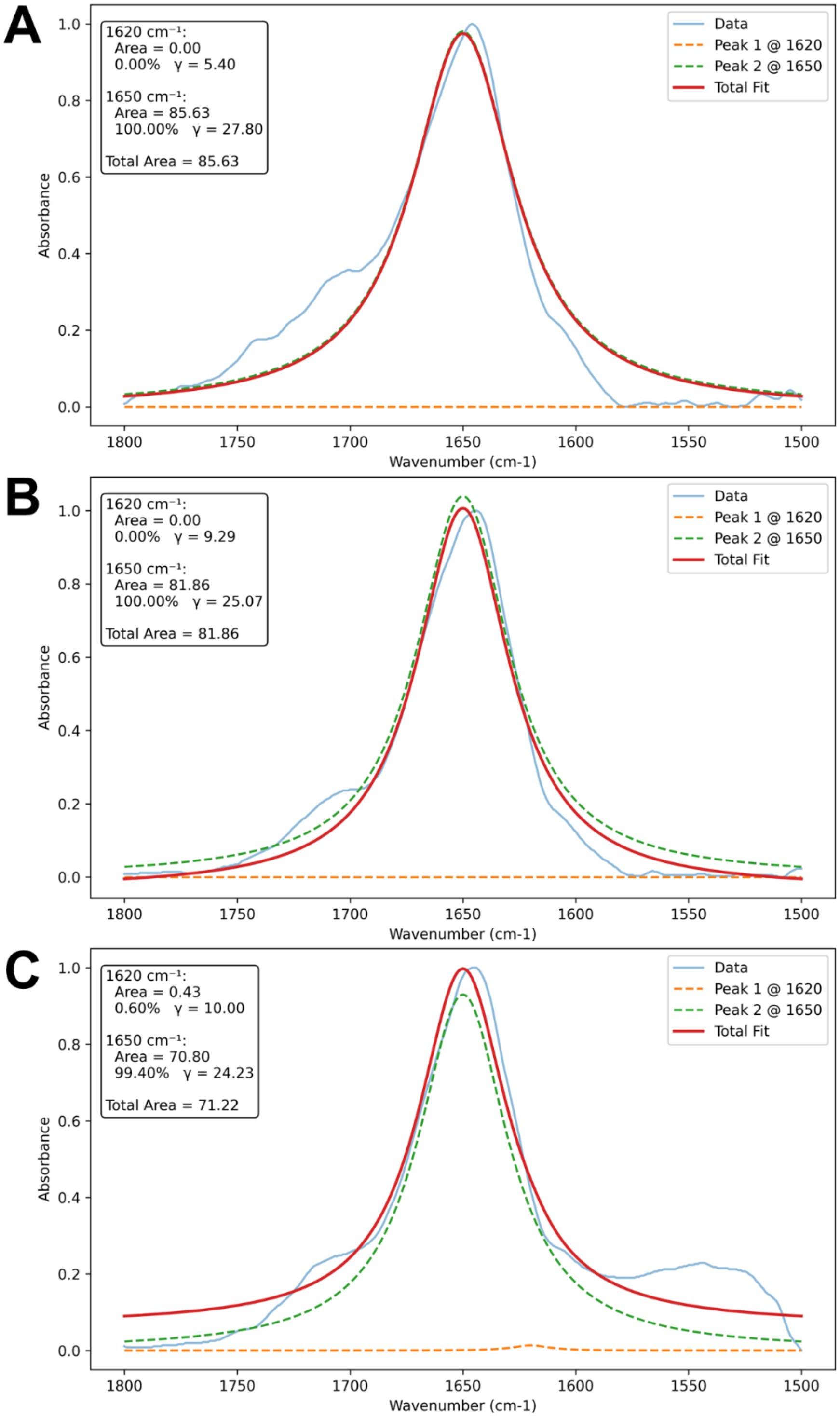

Figure S56. FTIR spectrum of **L4** 1 mM (solid blue) with an applied Lorentzian function to quantify the structural contributions of β-sheet at 1620 cm^{-1} (dotted orange) and random aggregate at 1650 cm^{-1}(dotted green), and a combined fit of the two peaks (solid red). (A) **L4** 1 mM 0 hours, (B) **L4** 1 mM 24 hours, (C) **L4** 1 mM 96 hours.

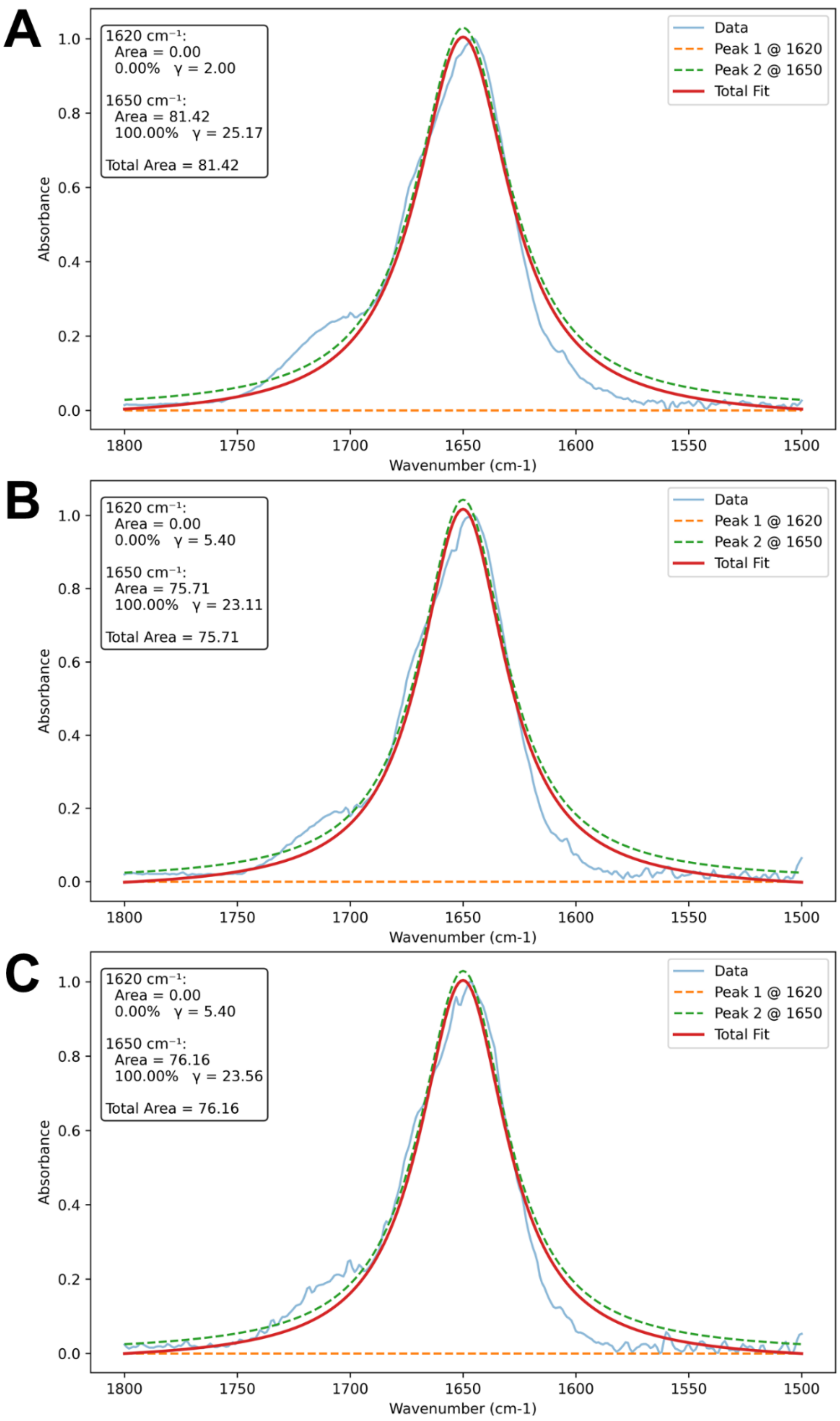

Figure S57. FTIR spectrum of **L4** 2 mM (solid blue) with an applied Lorentzian function to quantify the structural contributions of β-sheet at 1620 cm^{-1} (dotted orange) and random aggregate at 1650 cm^{-1}(dotted green), and a combined fit of the two peaks (solid red). (A) **L4** 2 mM 0 hours, (B) **L4** 2 mM 24 hours, (C) **L4** 2 mM 96 hours.

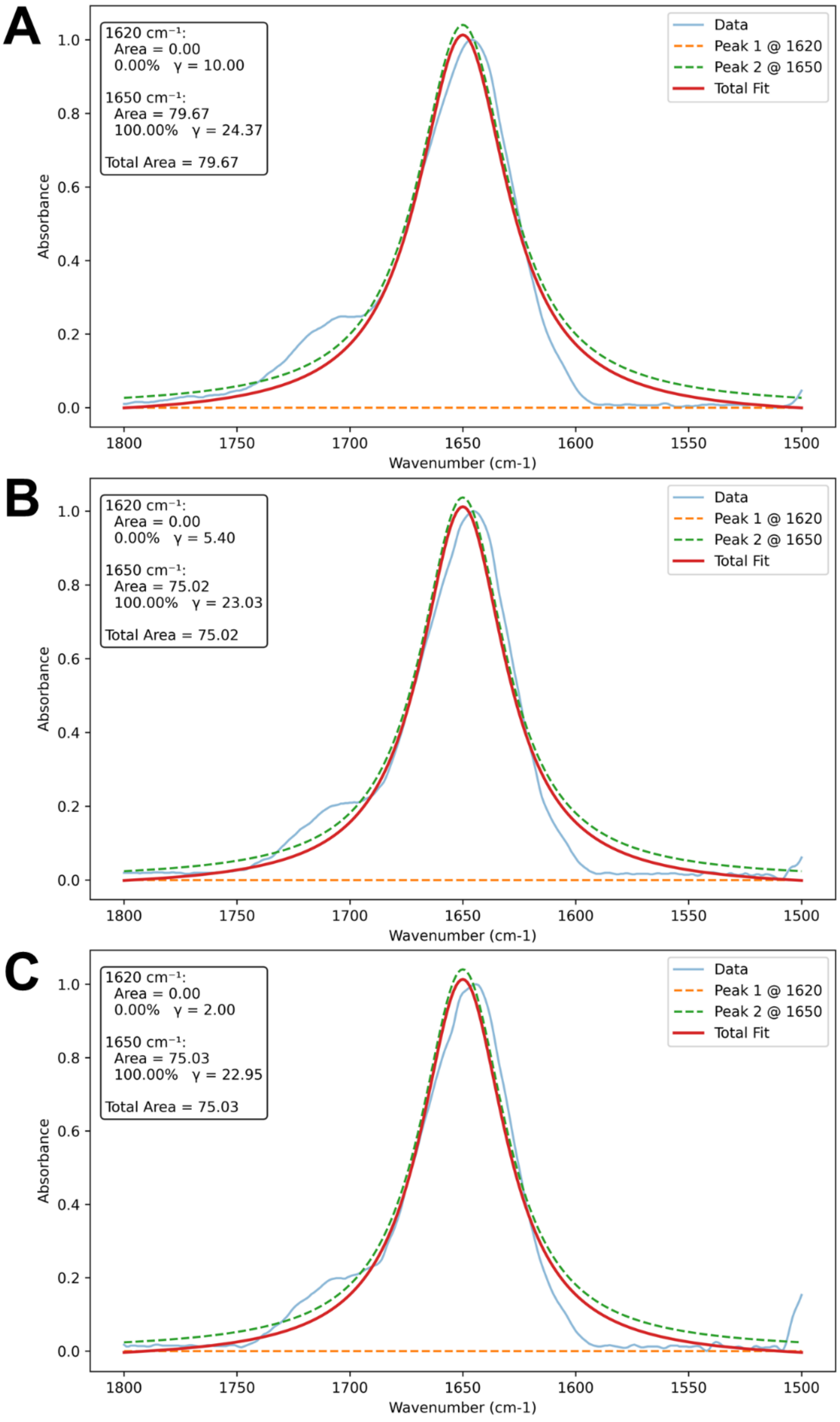

Figure S58. FTIR spectrum of **L4** 4 mM (solid blue) with an applied Lorentzian function to quantify the structural contributions of β-sheet at 1620 cm^{-1} (dotted orange) and random aggregate at 1650 cm^{-1}(dotted green), and a combined fit of the two peaks (solid red). (A) **L4** 4 mM 0 hours, (B) **L4** 4 mM 24 hours, (C) **L4** 4 mM 96 hours.

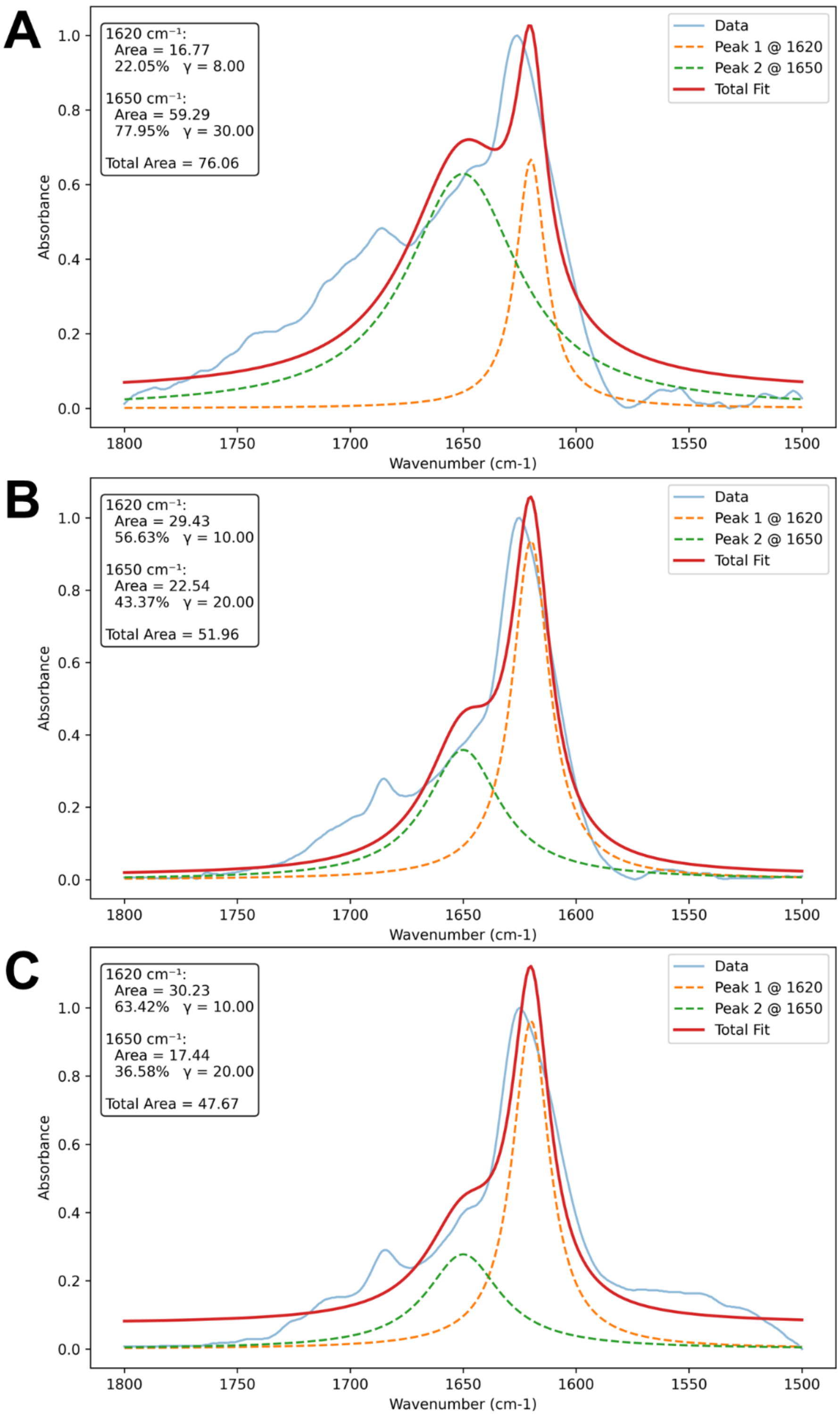

Figure S59. FTIR spectrum of **L4/D4** 1 mM (solid blue) with an applied Lorentzian function to quantify the structural contributions of β-sheet at 1620 cm^{-1} (dotted orange) and random aggregate at 1650 cm^{-1}(dotted green), and a combined fit of the two peaks (solid red). (A) **L4/D4** 1 mM 0 hours, (B) **L4/D4** 1 mM 24 hours, (C) **L4/D4** 1 mM 96 hours.

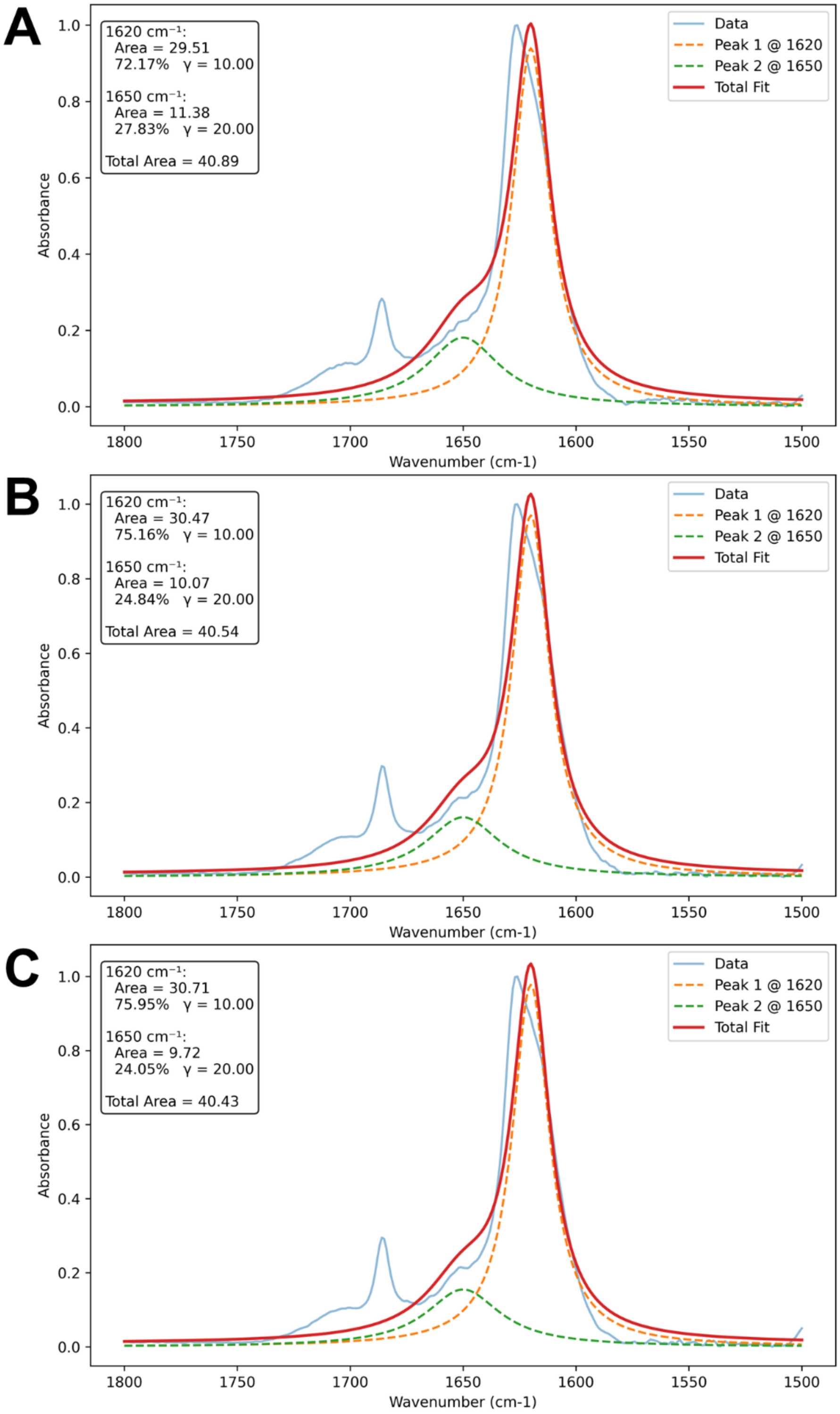

Figure S60. FTIR spectrum of **L4/D4** 2 mM (solid blue) with an applied Lorentzian function to quantify the structural contributions of β-sheet at 1620 cm^{-1} (dotted orange) and random aggregate at 1650 cm^{-1}(dotted green), and a combined fit of the two peaks (solid red). (A) **L4/D4** 2 mM 0 hours, (B) **L4/D4** 2 mM 24 hours, (C) **L4/D4** 2 mM 96 hours.

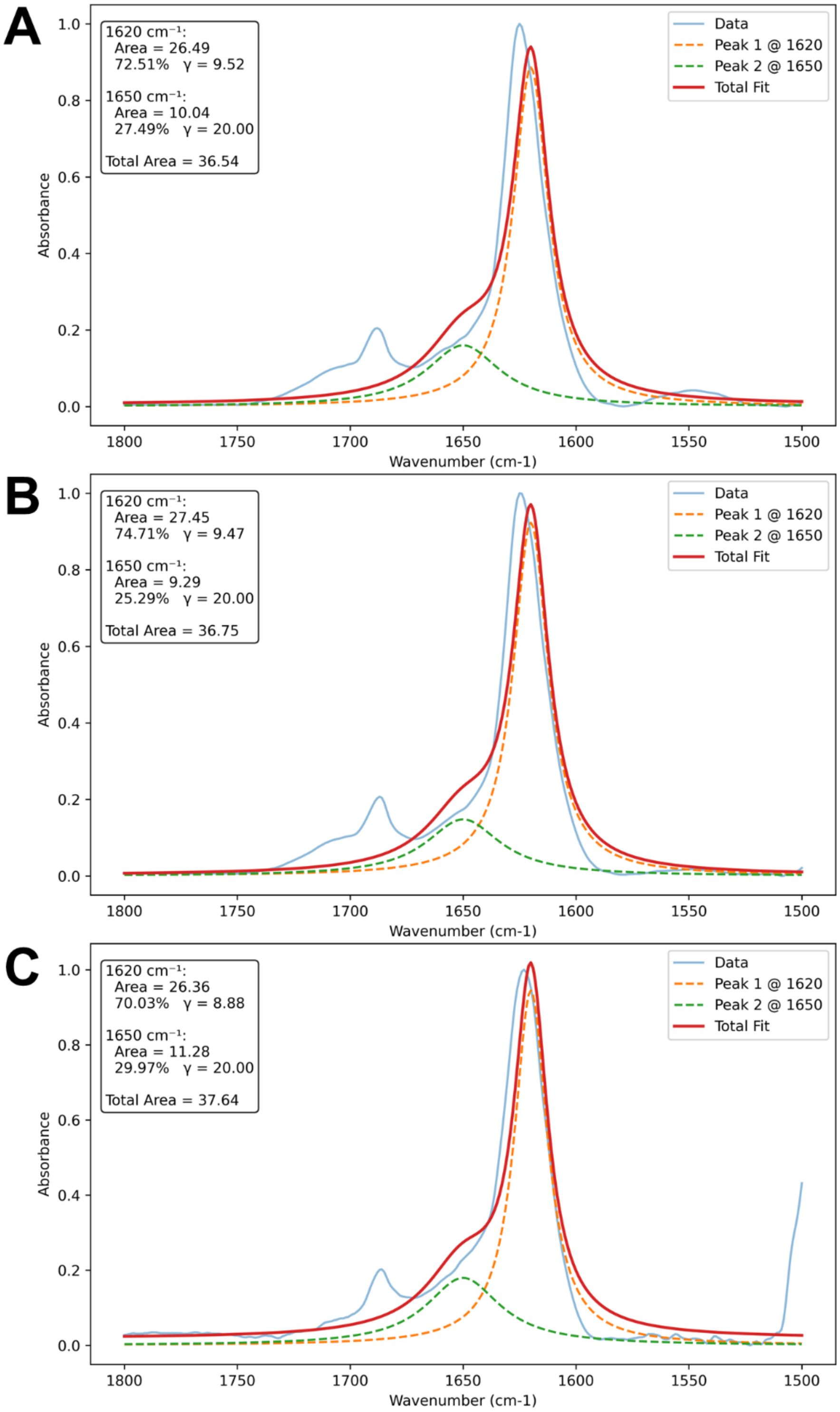

Figure S61. FTIR spectrum of **L4/D4** 4 mM (solid blue) with an applied Lorentzian function to quantify the structural contributions of β-sheet at 1620 cm^{-1} (dotted orange) and random aggregate at 1650 cm^{-1}(dotted green), and a combined fit of the two peaks (solid red). (A) **L4/D4** 4 mM 0 hours, (B) **L4/D4** 4 mM 24 hours, (C) **L4/D4** 4 mM 96 hours.

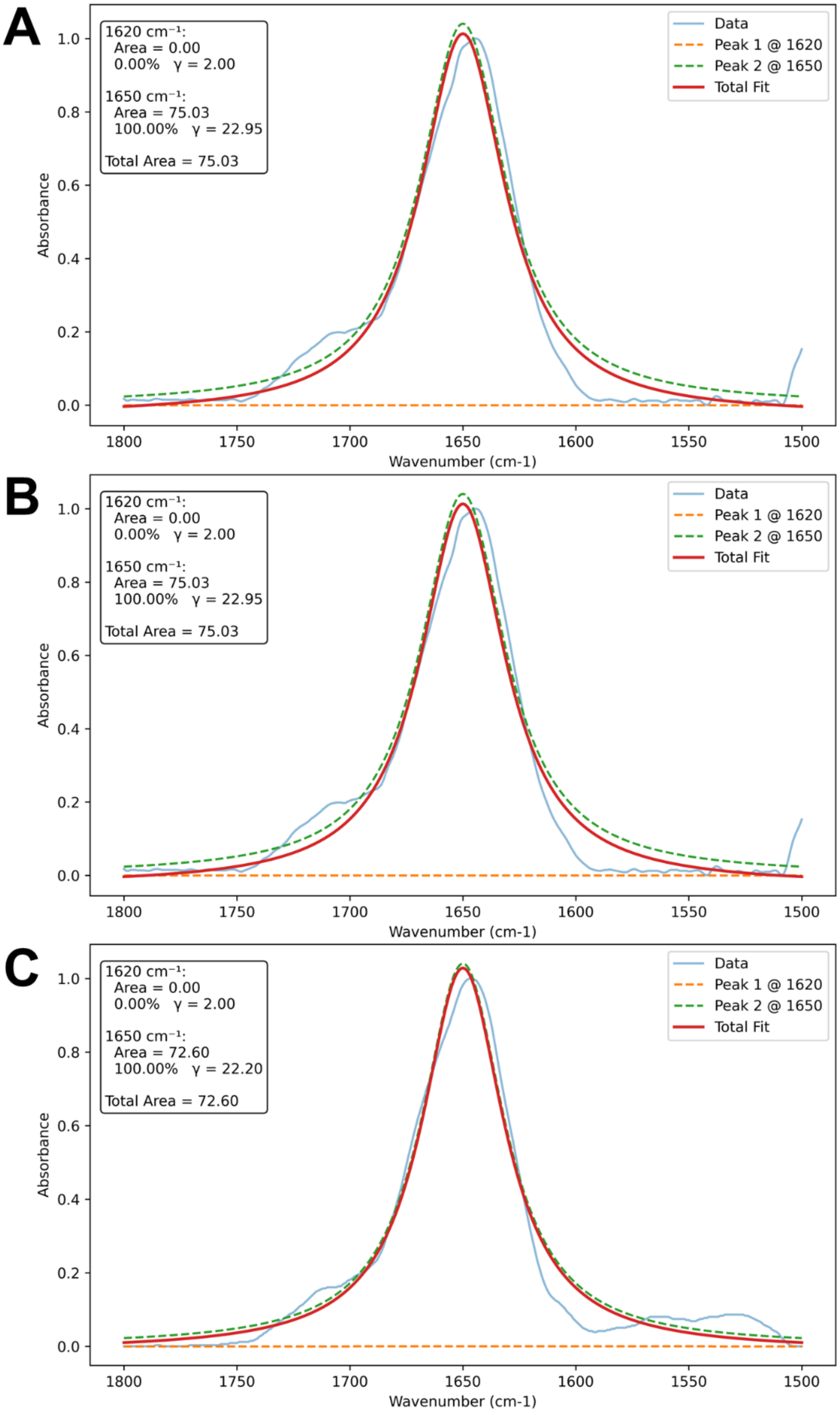

Figure S62. FTIR spectrum of **L5** 1 mM (solid blue) with an applied Lorentzian function to quantify the structural contributions of β-sheet at 1620 cm^{-1} (dotted orange) and random aggregate at 1650 cm^{-1}(dotted green), and a combined fit of the two peaks (solid red). (A) **L5** 1 mM 0 hours, (B) **L5** 1 mM 24 hours, (C) **L5** 4 mM 96 hours.

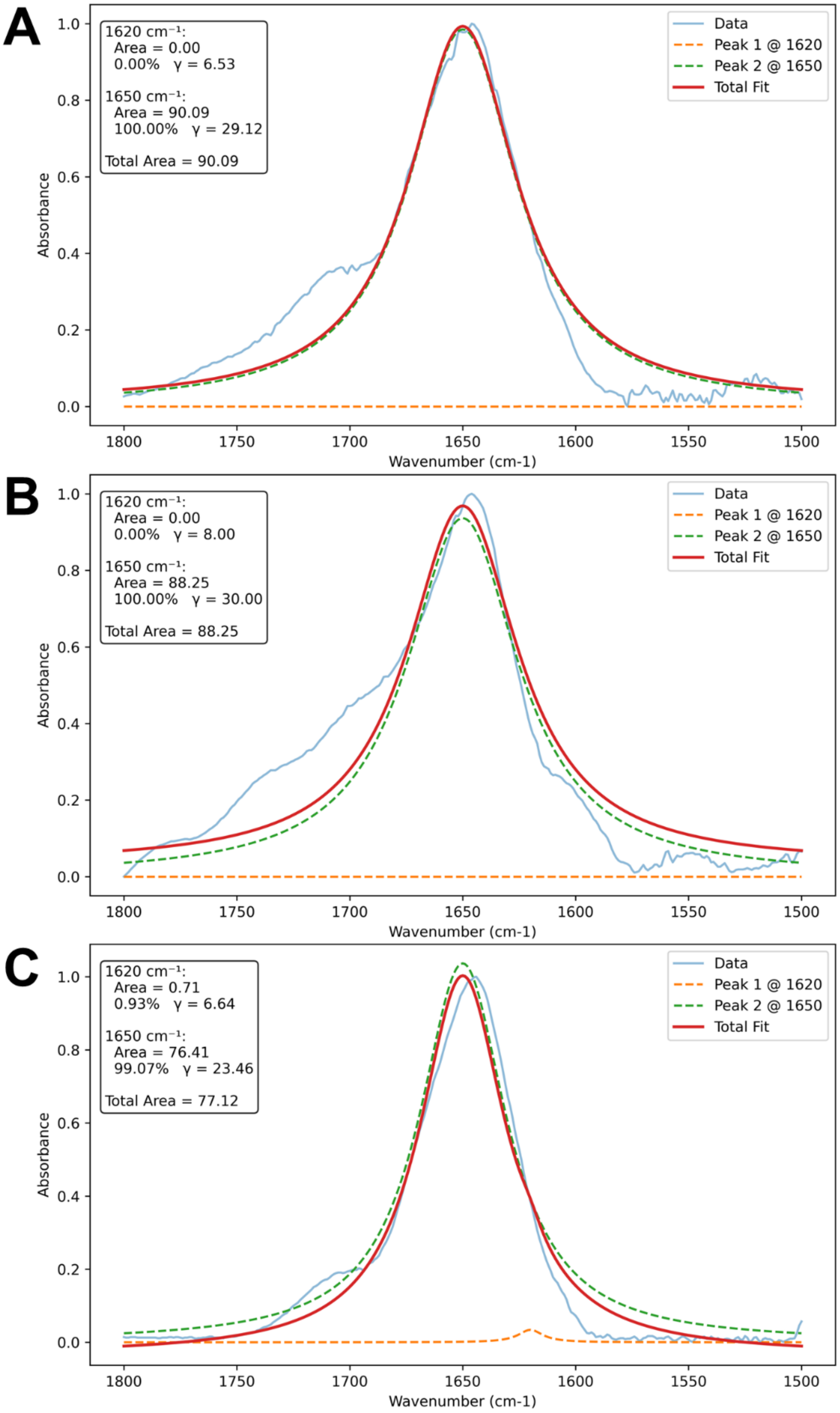

Figure S63. FTIR spectrum of **L5** 2 mM (solid blue) with an applied Lorentzian function to quantify the structural contributions of β-sheet at 1620 cm^{-1} (dotted orange) and random aggregate at 1650 cm^{-1}(dotted green), and a combined fit of the two peaks (solid red). (A) **L5** 2 mM 0 hours, (B) **L5** 2 mM 24 hours, (C) **L5** 2 mM 96 hours.

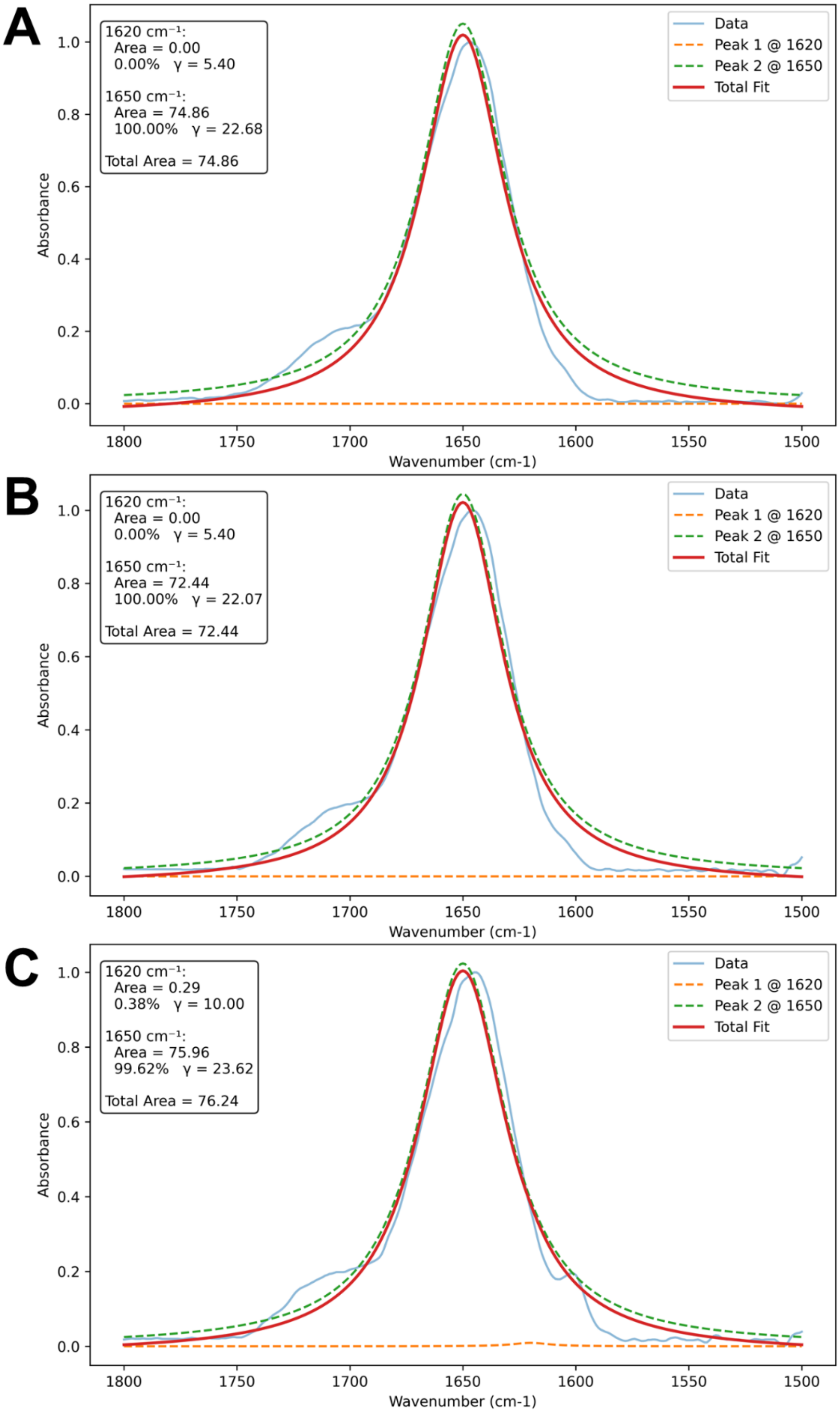

Figure S64. FTIR spectrum of **L5** 4 mM (solid blue) with an applied Lorentzian function to quantify the structural contributions of β-sheet at 1620 cm^{-1} (dotted orange) and random aggregate at 1650 cm^{-1}(dotted green), and a combined fit of the two peaks (solid red). (A) **L5** 4 mM 0 hours, (B) **L5** 4 mM 24 hours, (C) **L5** 4 mM 96 hours.

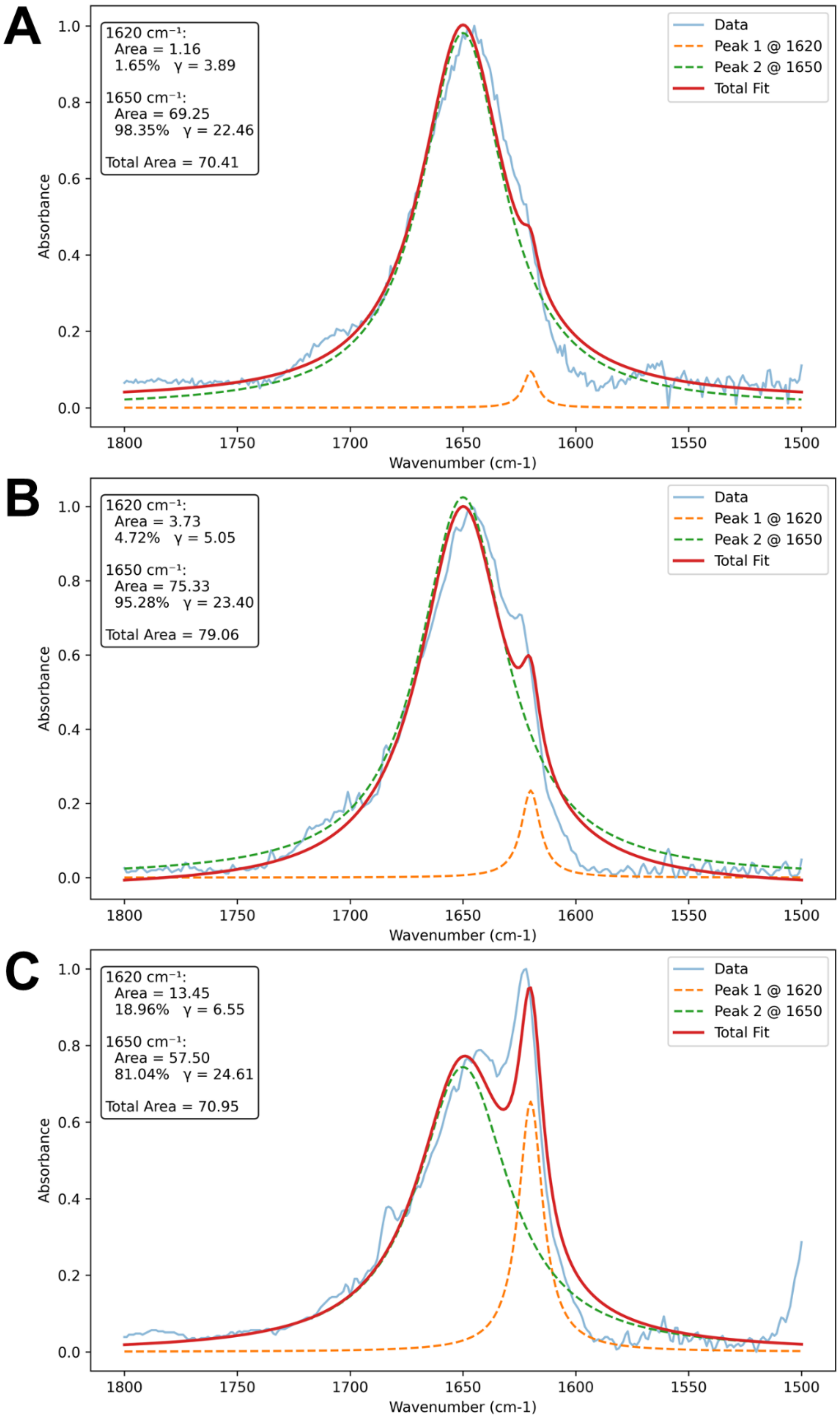

Figure S65. FTIR spectrum of **L5/D5** 1 mM (solid blue) with an applied Lorentzian function to quantify the structural contributions of β-sheet at 1620 cm^{-1} (dotted orange) and random aggregate at 1650 cm^{-1}(dotted green), and a combined fit of the two peaks (solid red). (A) **L5/D5** 1 mM 0 hours, (B) **L5/D5** 1 mM 24 hours, (C) **L5/D5** 1 mM 96 hours.

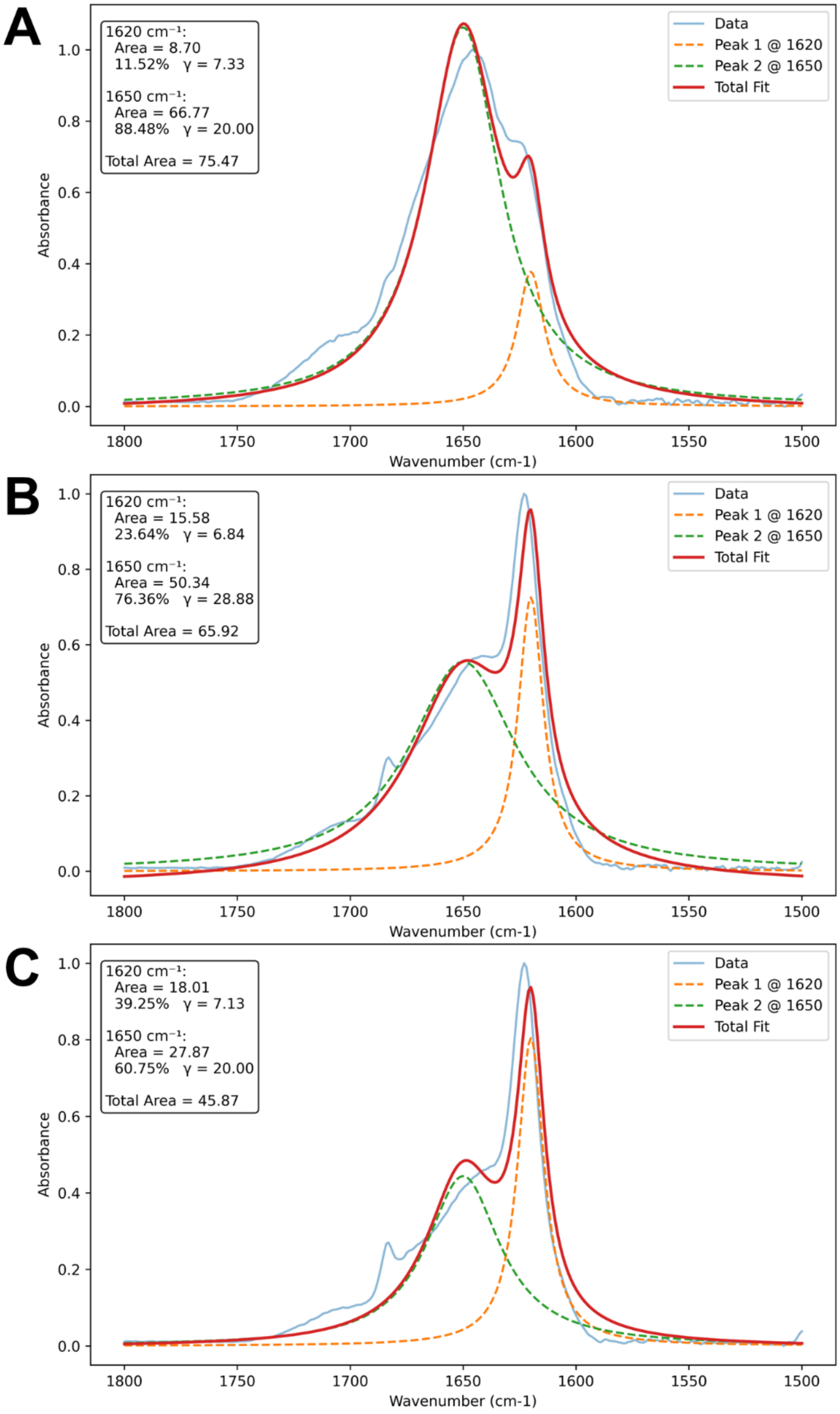

Figure S66. FTIR spectrum of **L5/D5** 2 mM (solid blue) with an applied Lorentzian function to quantify the structural contributions of β-sheet at 1620 cm^{-1} (dotted orange) and random aggregate at 1650 cm^{-1}(dotted green), and a combined fit of the two peaks (solid red). (A) **L5/D5** 2 mM 0 hours, (B) **L5/D5** 2 mM 24 hours, (C) **L5/D5** 2 mM 96 hours.

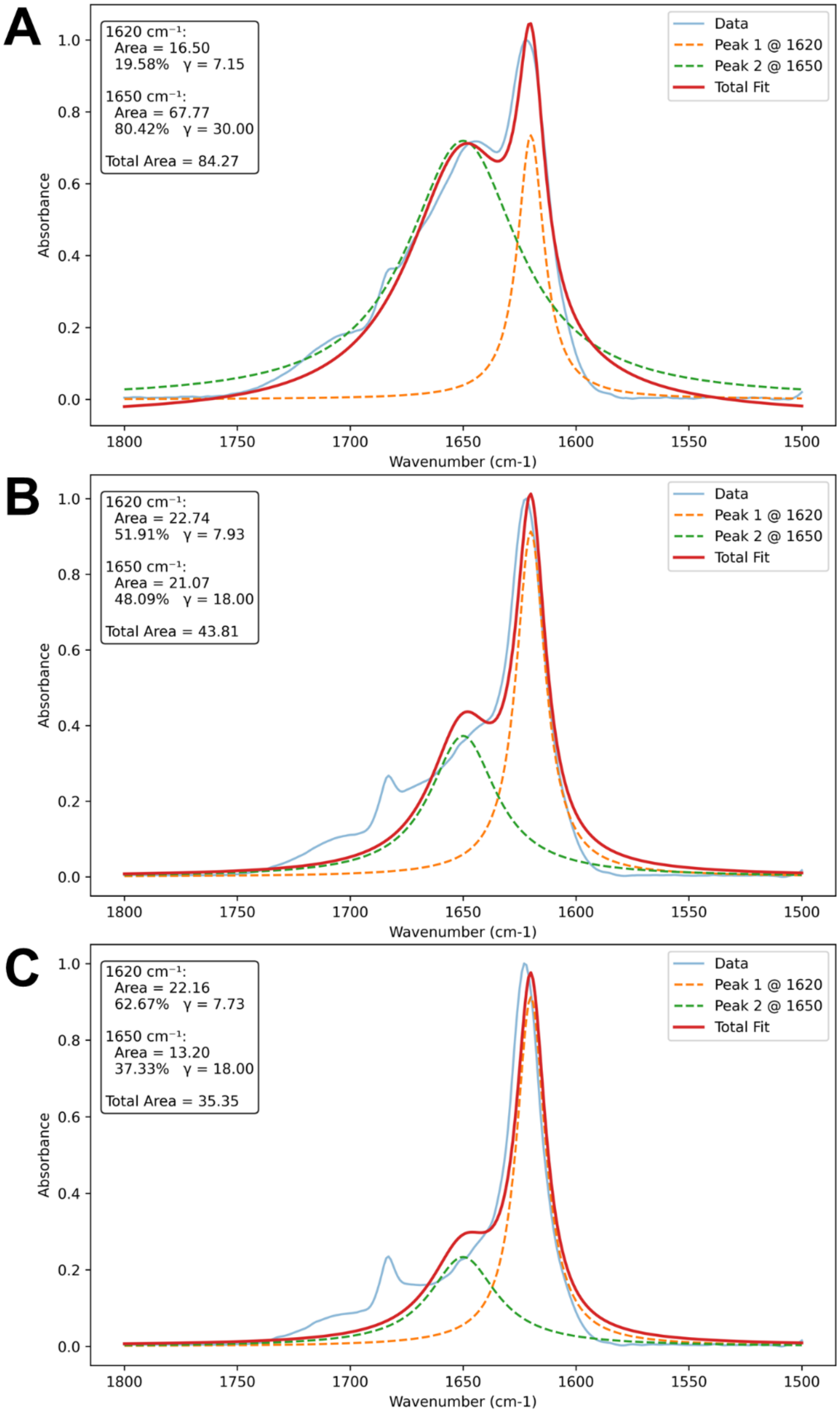

Figure S67. FTIR spectrum of **L5/D5** 4 mM (solid blue) with an applied Lorentzian function to quantify the structural contributions of β-sheet at 1620 cm^{-1} (dotted orange) and random aggregate at 1650 cm^{-1}(dotted green), and a combined fit of the two peaks (solid red). (A) **L5/D5** 4 mM 0 hours, (B) **L5/D5** 4 mM 24 hours, (C) **L5/D5** 4 mM 96 hours.

Lorentzian Function

The amide I absorbance spectrum was modeled as the sum of two Lorentzian functions and a constant baseline:

$$A(\tilde{\nu}) = L_1(\tilde{\nu}) + L_2(\tilde{\nu}) + c$$

where $\tilde{\nu}$ is the wavenumber (cm^{-1}) and c is a constant baseline offset.

Each Lorentzian component $L_i(\tilde{\nu})$ is defined as:

$$L_i(\tilde{\nu}) = \frac{a_i}{1 + \left(\frac{\tilde{\nu} - \tilde{\nu}_i}{\gamma_i}\right)^2}, \quad i = 1,2$$

where $\tilde{\nu}_i$is the center wavenumber of peak i, $a_i$is the peak amplitude, and $\gamma_i$is the half-width at half-maximum (HWHM).

For amide I band deconvolution, the peak center positions were fixed at:

$$\tilde{\nu}_1 = 1620\ \mathrm{cm}^{-1}, \quad \tilde{\nu}_2 = 1650\ \mathrm{cm}^{-1}$$

corresponding to β-sheet–associated and non–β-sheet (e.g., α-helical or disordered) secondary structure contributions, respectively.

The integrated area of each Lorentzian peak was calculated analytically as:

$$\mathrm{Area}_i = \int_{-\infty}^{\infty} L_i(\tilde{\nu})\ d\tilde{\nu} = a_i \pi \gamma_i$$

The fractional contribution of each spectral component was determined by normalizing individual peak areas to the total amide I area

$$f_i = \frac{\mathrm{Area}_i}{\sum_j \mathrm{Area}_j}$$

where Area_i represents the integrated absorbance contribution of peak i.

Peak Fitting Code

```
#==========================================================
# Importing required libraries for peak fitting
#==========================================================
import numpy as np
import pandas as pd
import matplotlib.pyplot as plt
from scipy.optimize import curve_fit

# ========================================================
# Load FTIR data
# ========================================================
# Data are stored in an Excel file with:
#   - x: wavenumber (cm^-1)
#   - y: absorbance for the specified sample condition
file = 'IR_coassembly_new.xlsx'
df = pd.read_excel(file)

x = df['x'].values                      # Wavenumber axis (cm^-1)
y = df['y'].values              # Absorbance spectrum

# ========================================================
# Define peak positions (fixed during fitting)
# ========================================================
# Peak positions are chosen based on known amide I band assignments
# and visual inspection of the spectra.
peak_x1 = 1620   # Main β-sheet–associated peak (cm^-1)
peak_x2 = 1650   # Shoulder / secondary structure contribution (cm^-1)
```

```
print(f"Using manually selected peaks: {peak_x1} and {peak_x2} cm-1")

# ==============================================================
# Define fitting model: sum of two Lorentzian functions + baseline
# ==============================================================
def two_lor(x, a1, gamma1, a2, gamma2, c):
    """
    Two-Lorentzian model with fixed peak centers.

    Parameters
    ----------
    a1, a2 : float
        Peak amplitudes
    gamma1, gamma2 : float
        Half-width at half-maximum (HWHM) parameters
    c : float
        Constant baseline offset
    """
    l1 = a1 / (1 + ((x - peak_x1) / gamma1)**2)
    l2 = a2 / (1 + ((x - peak_x2) / gamma2)**2)
    return l1 + l2 + c

# ==============================================================
# Initial parameter guesses
# ==============================================================
# Initial guesses are chosen to ensure convergence without biasing the fit
initial_guess = [
```

```
    np.max(y),        # a1: main peak amplitude
    6,                # gamma1: initial width estimate
    np.max(y) / 4,    # a2: smaller shoulder amplitude
    6,                # gamma2: initial width estimate
    0.0               # c: baseline offset
]

# ============================================================
# Parameter bounds
# ============================================================
# Widths are constrained to physically reasonable values for amide I bands
lower_bounds = [0, 2, 0, 2, -np.inf]
upper_bounds = [np.inf, 8, np.inf, 20, np.inf]

# ============================================================
# Perform nonlinear least-squares fitting
# ============================================================
popt, _ = curve_fit(
    two_lor,
    x,
    y,
    p0=initial_guess,
    bounds=(lower_bounds, upper_bounds)
)

a1, gamma1, a2, gamma2, c = popt

# ============================================================
```

```
# Reconstruct individual peak components
# ============================================================
peak1 = a1 / (1 + ((x - peak_x1) / gamma1)**2)
peak2 = a2 / (1 + ((x - peak_x2) / gamma2)**2)
fit_total = peak1 + peak2 + c

# ============================================================
# Quantify peak areas
# ============================================================
# Analytical integral of a Lorentzian: Area = a * π * gamma
area1 = a1 * np.pi * gamma1
area2 = a2 * np.pi * gamma2
total_area = area1 + area2

print("\n=== Lorentzian Fit Results ===")
print(f"Peak 1 @ {peak_x1:.2f} cm^-1 : Area = {area1:.2f} "
    f"({100*area1/total_area:.2f}%)   gamma = {gamma1:.2f}")
print(f"Peak 2 @ {peak_x2:.2f} cm^-1 : Area = {area2:.2f} "
    f"({100*area2/total_area:.2f}%)   gamma = {gamma2:.2f}")
print(f"Total Area = {total_area:.2f}")

# ============================================================
# Plot experimental data and fitted components
# ============================================================
plt.figure(figsize=(8, 5))
plt.plot(x, y, label='Experimental data', alpha=0.5)
plt.plot(x, peak1, '--', label=f'Peak @ {peak_x1} cm⁻¹')
plt.plot(x, peak2, '--', label=f'Peak @ {peak_x2} cm⁻¹')
```

```
plt.plot(x, fit_total, label='Total fit', linewidth=2)

plt.xlabel("Wavenumber (cm⁻¹)")
plt.ylabel("Absorbance")
plt.title("Title of the plot")
plt.legend()

# Standard FTIR convention (optional)
plt.gca().invert_xaxis()

plt.tight_layout()
plt.savefig("Title of the plot.png", dpi=600, bbox_inches="tight")
plt.show()
```

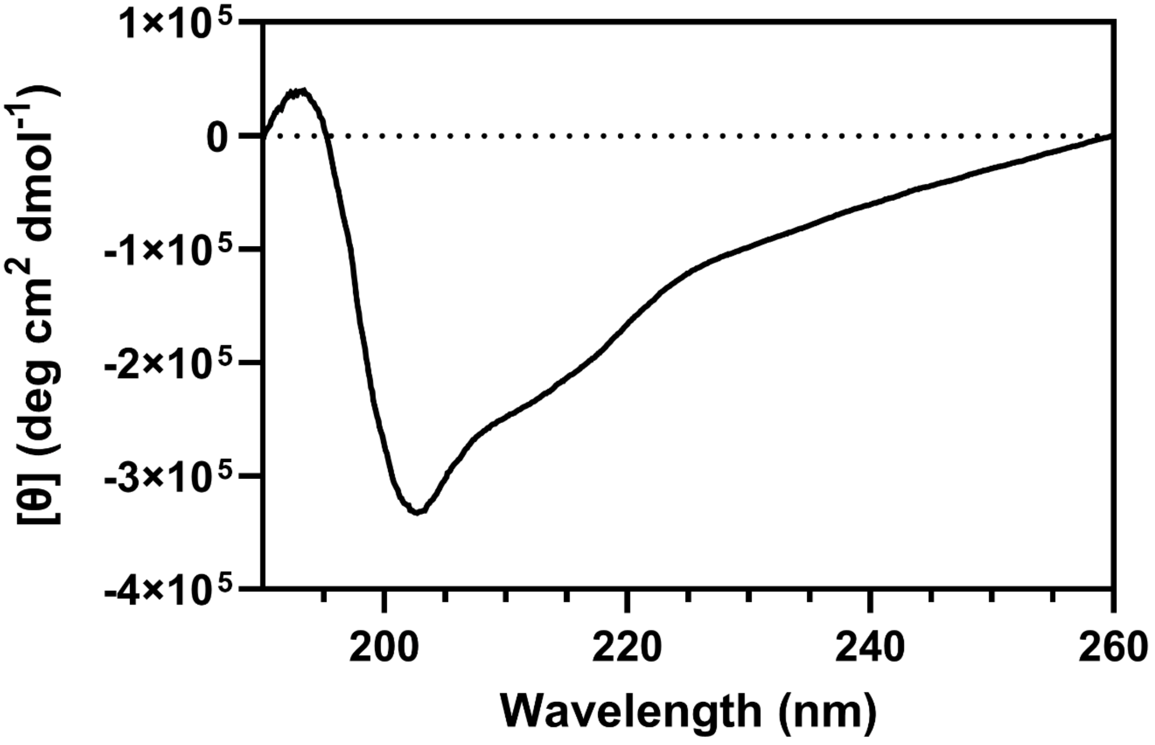

Figure S68. CD spectrum of self-assembled **L1** (1 mM) at 0 hours after assembly.

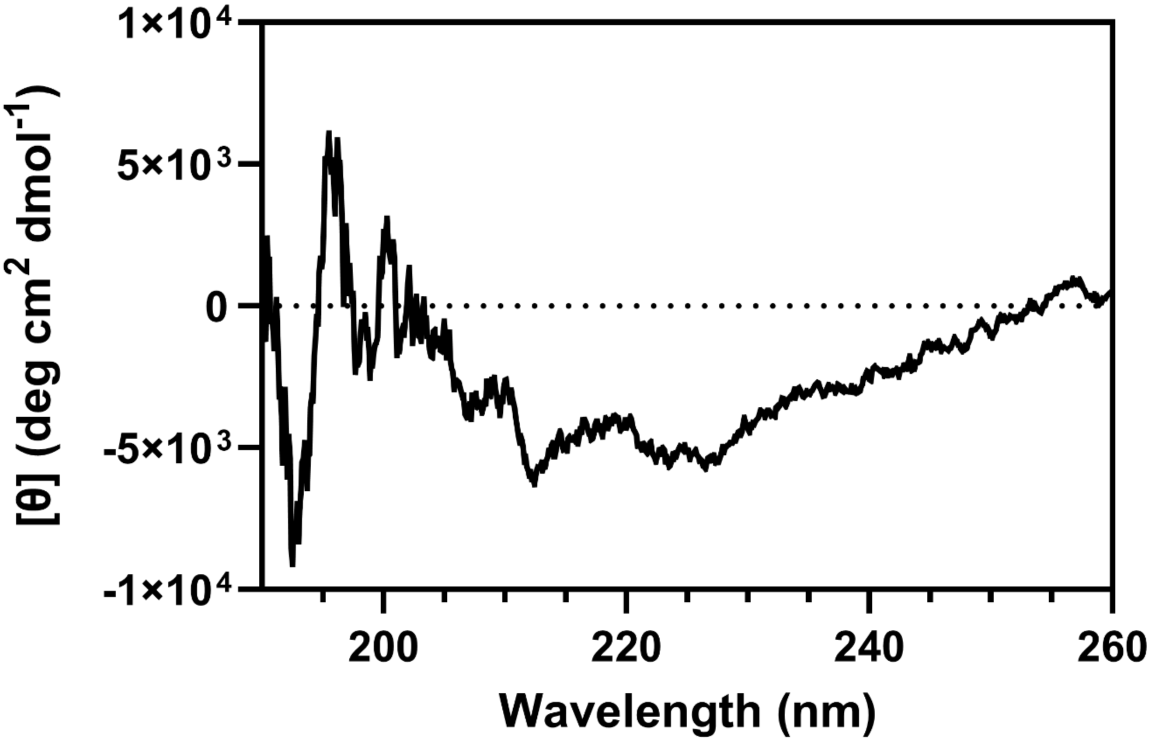

Figure S69. CD spectrum of coassembled **L1/D1** (1 mM) at 0 hours after assembly.

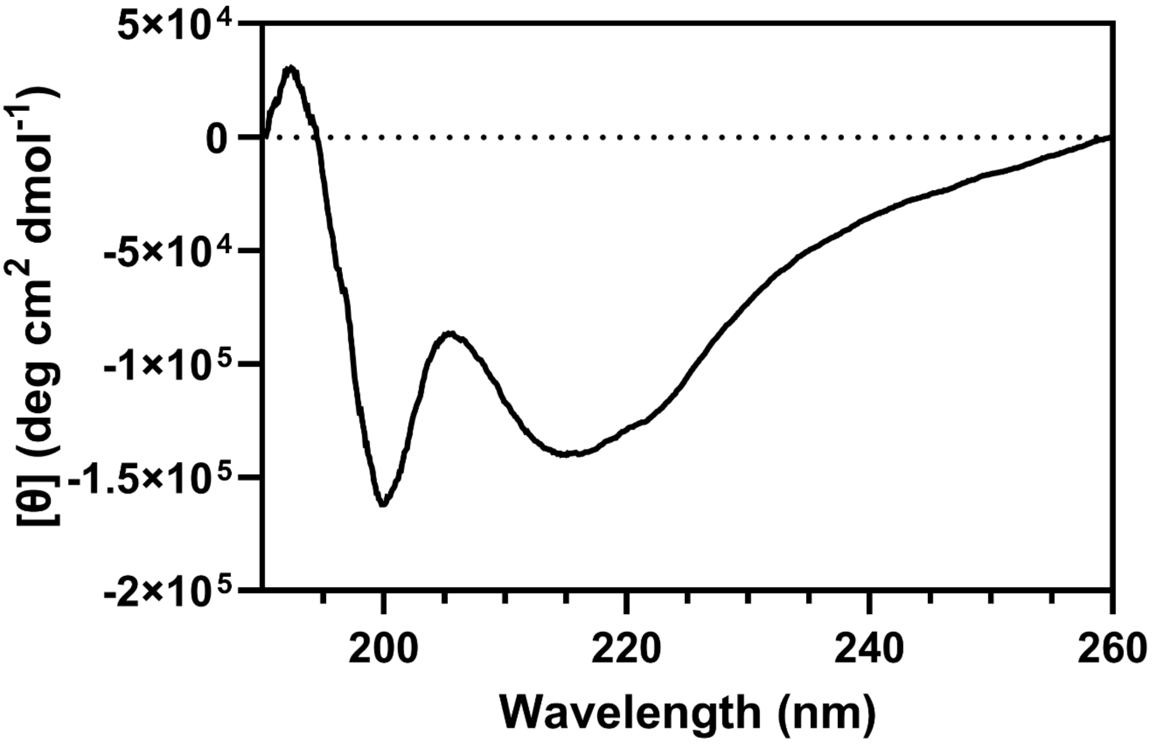

Figure S70. CD spectrum of self-assembled **L2** (1 mM) at 0 hours after assembly.

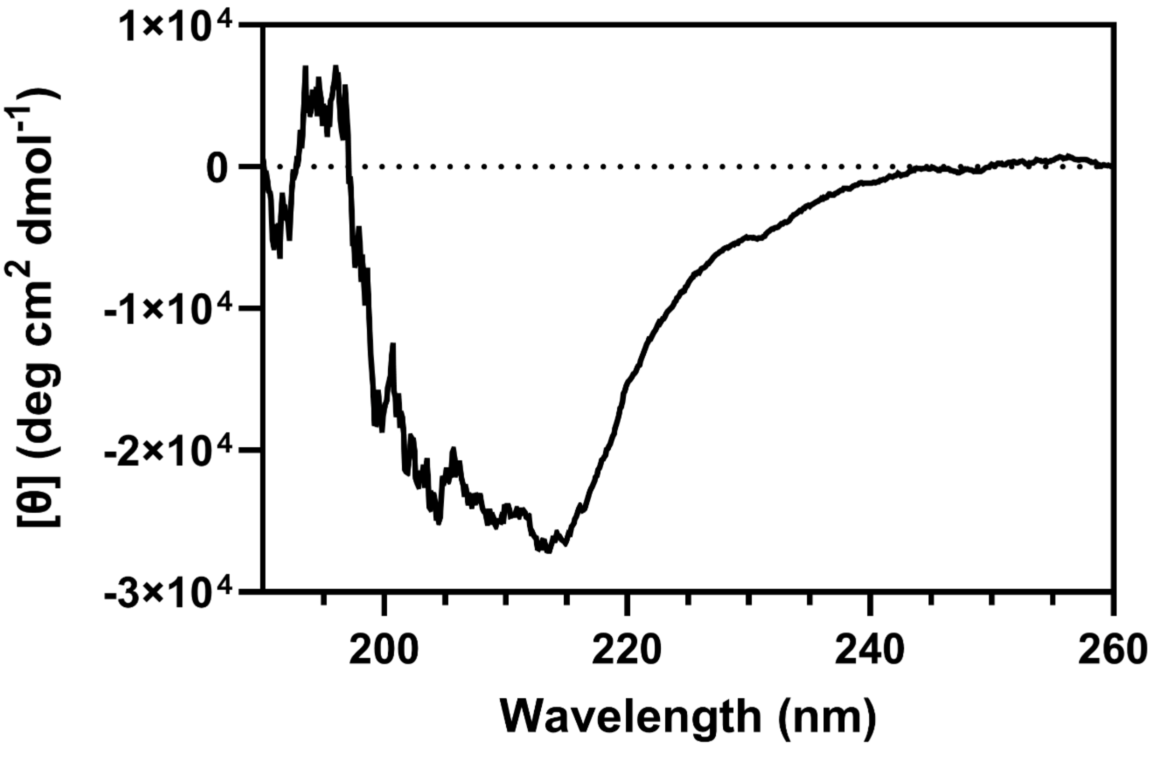

Figure S71. CD spectrum of coassembled **L2/D2** (1 mM) at 0 hours after assembly.

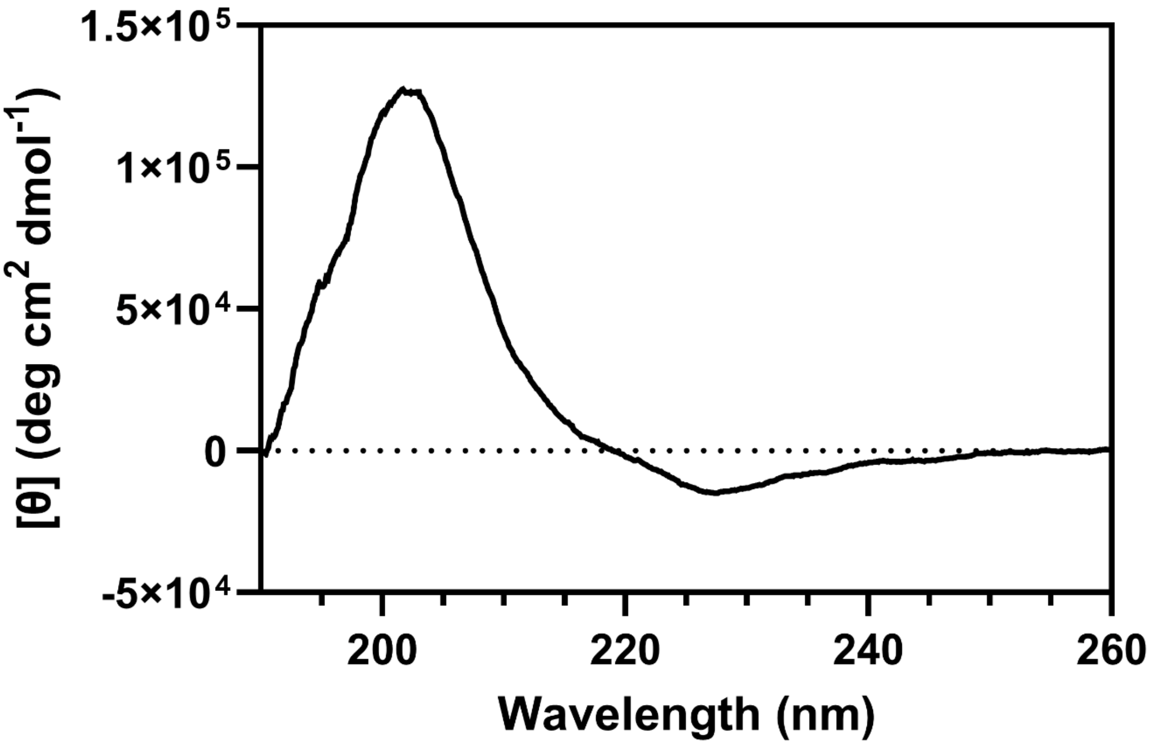

Figure S72. CD spectrum of self-assembled **L3** (1 mM) at 0 hours after assembly.

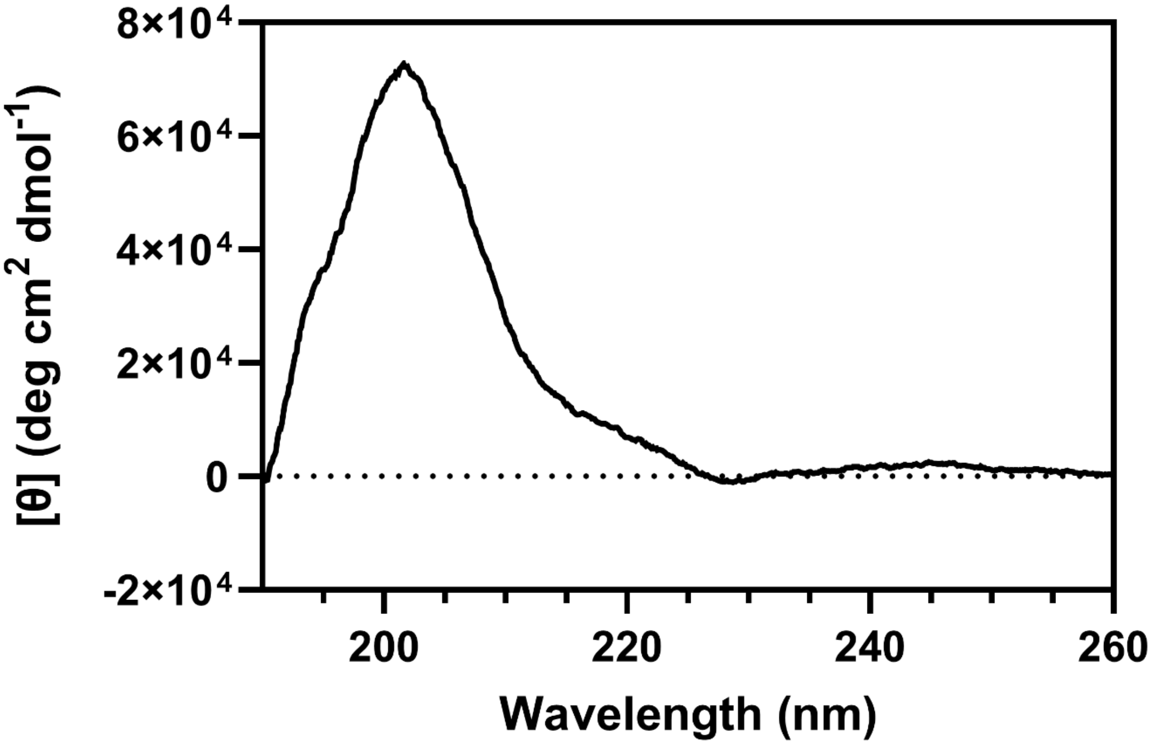

Figure S73. CD spectrum of self-assembled **L3** (2 mM) at 0 hours after assembly.

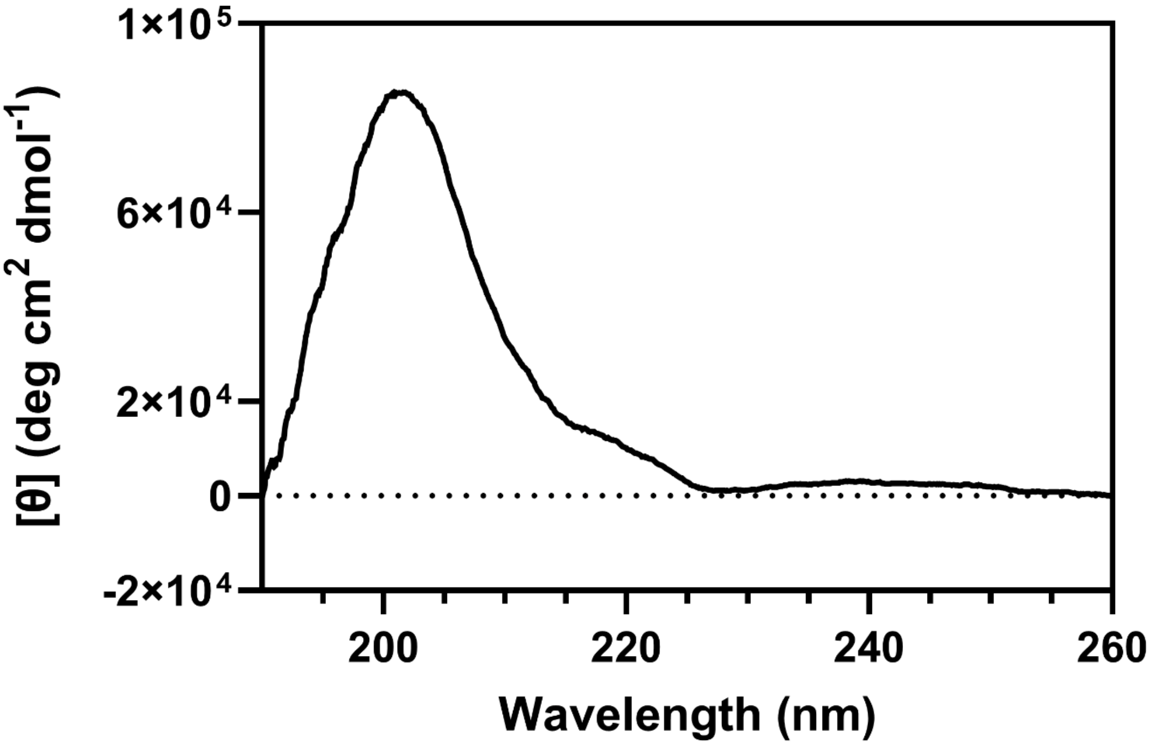

Figure S74. CD spectrum of self-assembled **L3** (4 mM) at 0 hours after assembly.

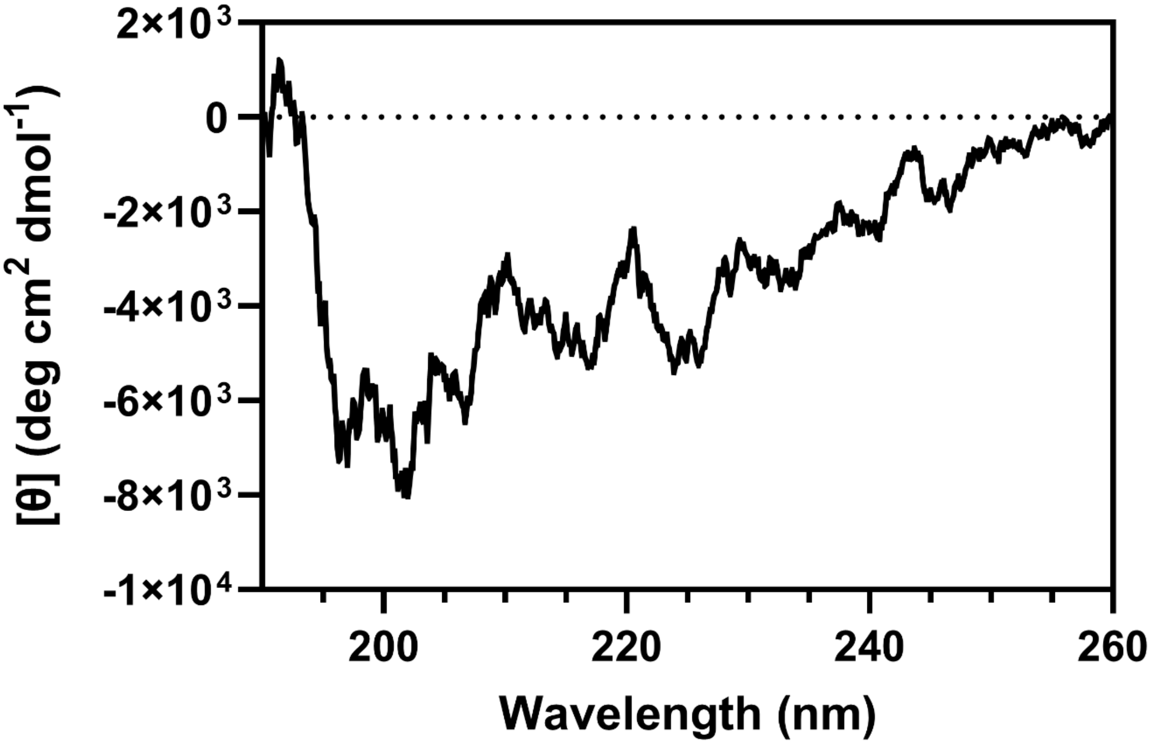

Figure S75. CD spectrum of coassembled **L3/D3** (1 mM) at 0 hours after assembly.

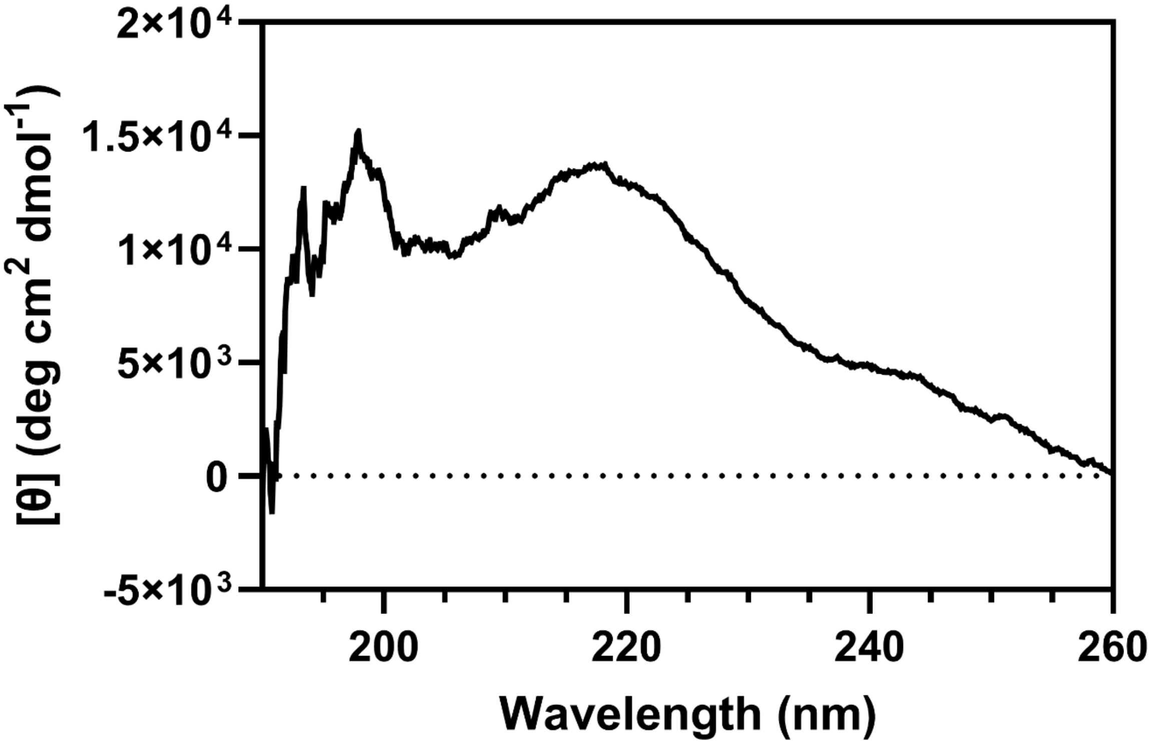

Figure S76. CD spectrum of self-assembled **L4** (1 mM) at 0 hours after assembly.

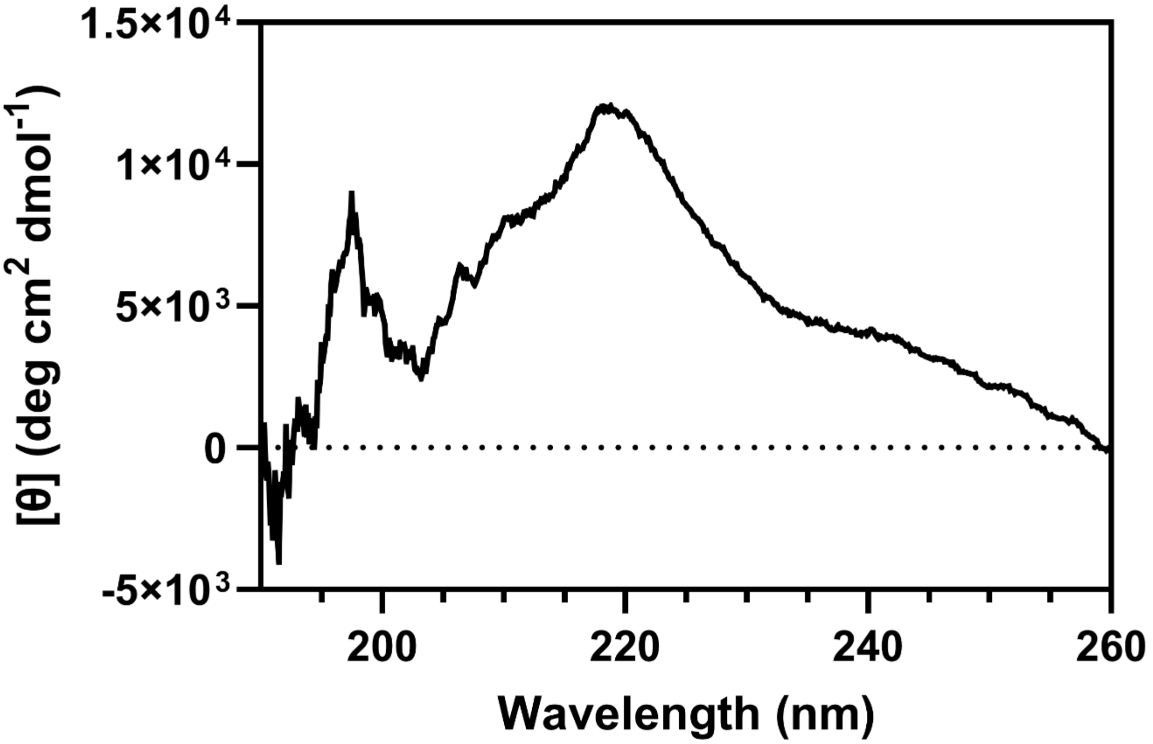

Figure S77. CD spectrum of self-assembled **L4** (2 mM) at 0 hours after assembly.

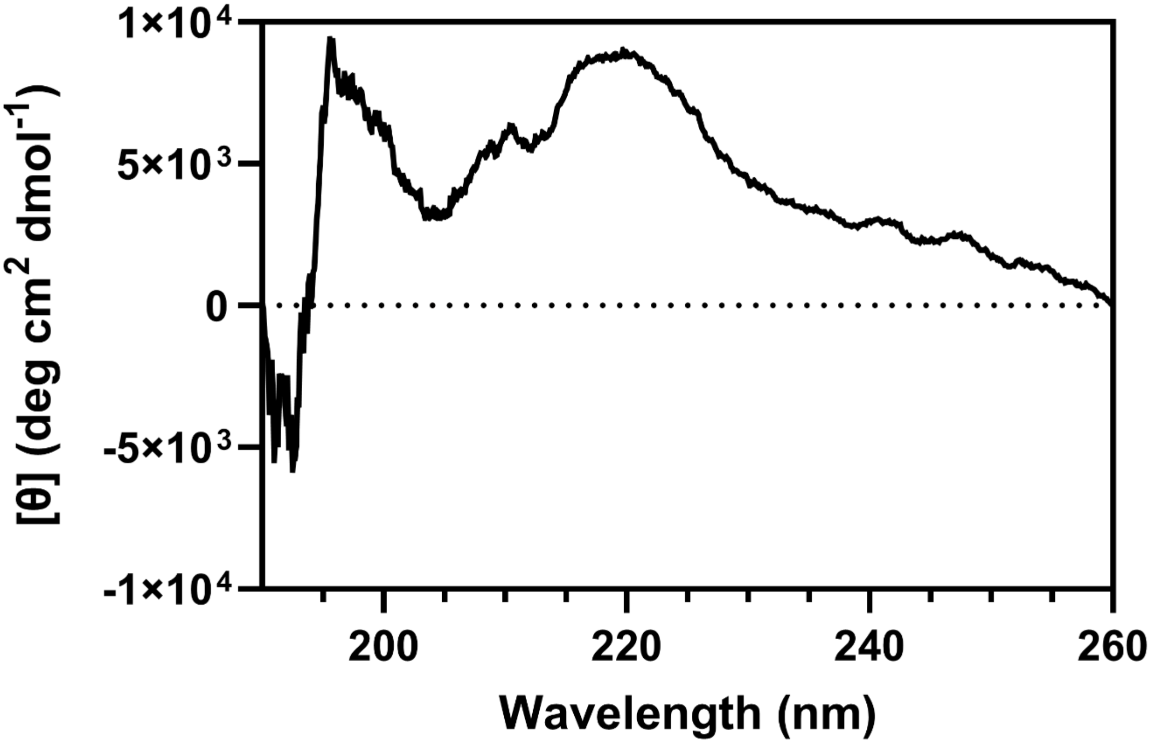

Figure S78. CD spectrum of self-assembled **L4** (4 mM) at 0 hours after assembly.

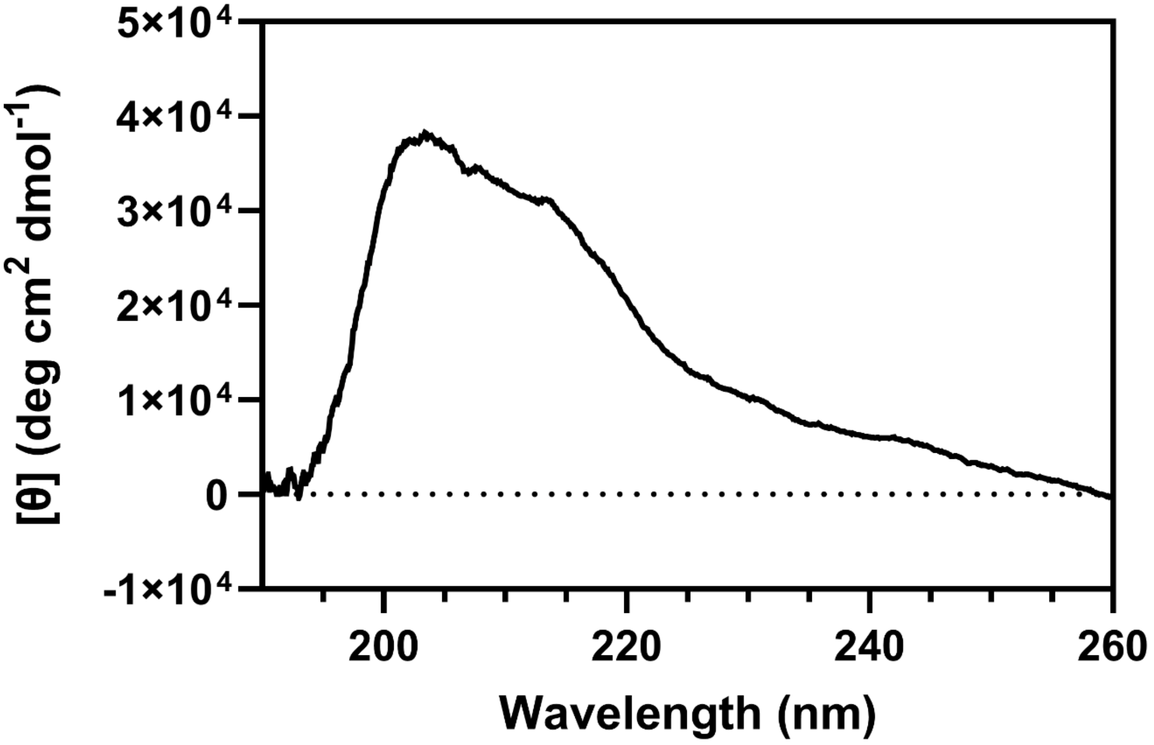

Figure S79. CD spectrum of coassembled **L4/D4** (1 mM) at 0 hours after assembly.

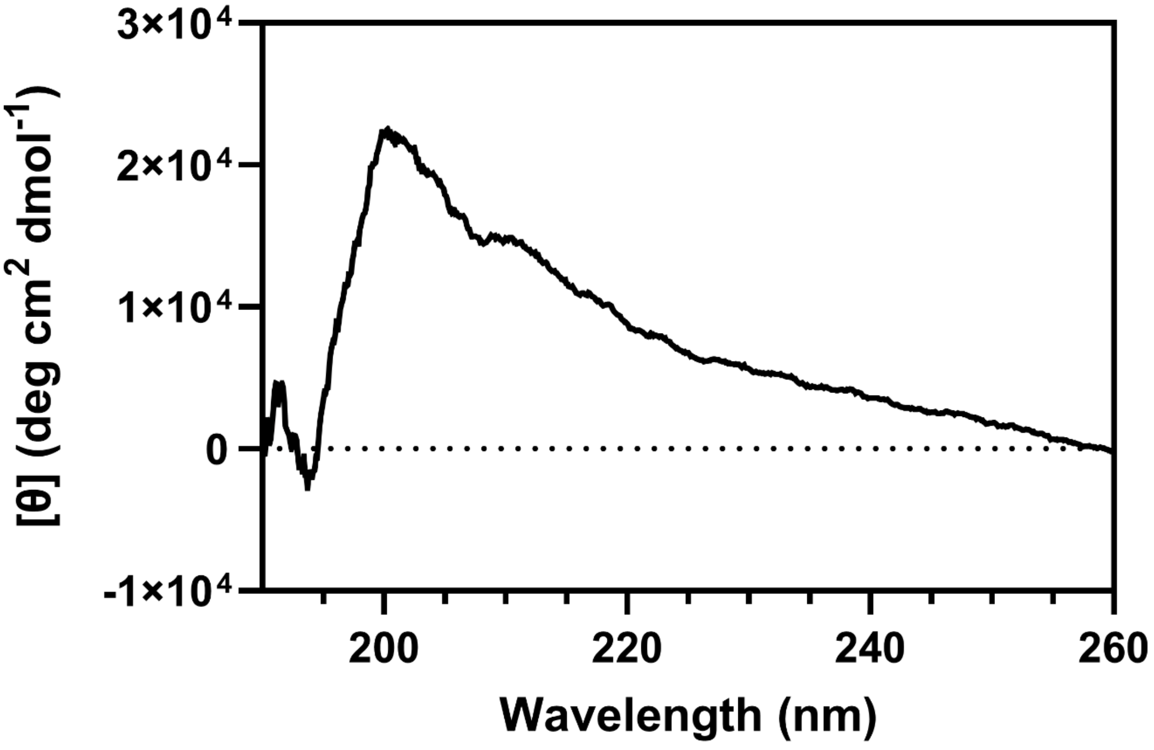

Figure S80. CD spectrum of coassembled **L4/D4** (2 mM) at 0 hours after assembly.

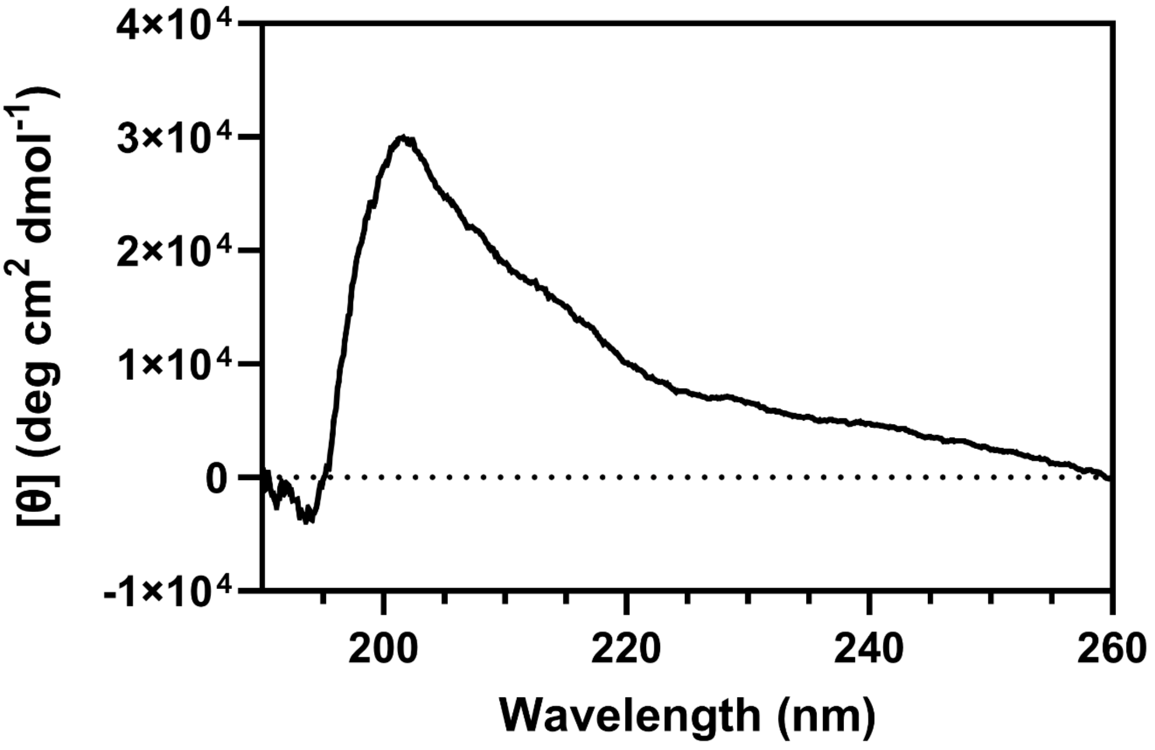

Figure S81. CD spectrum of coassembled **L4/D4** (4 mM) at 0 hours after assembly.

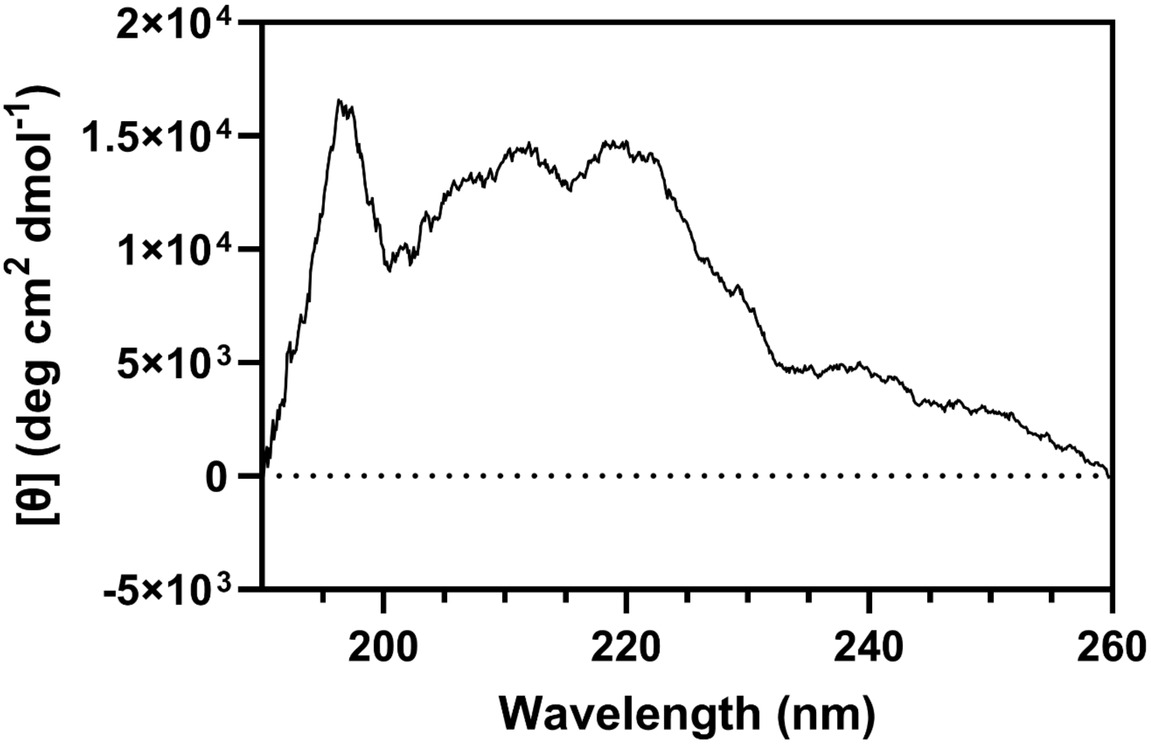

Figure S82. CD spectrum of self-assembled **L5** (1 mM) at 0 hours after assembly.

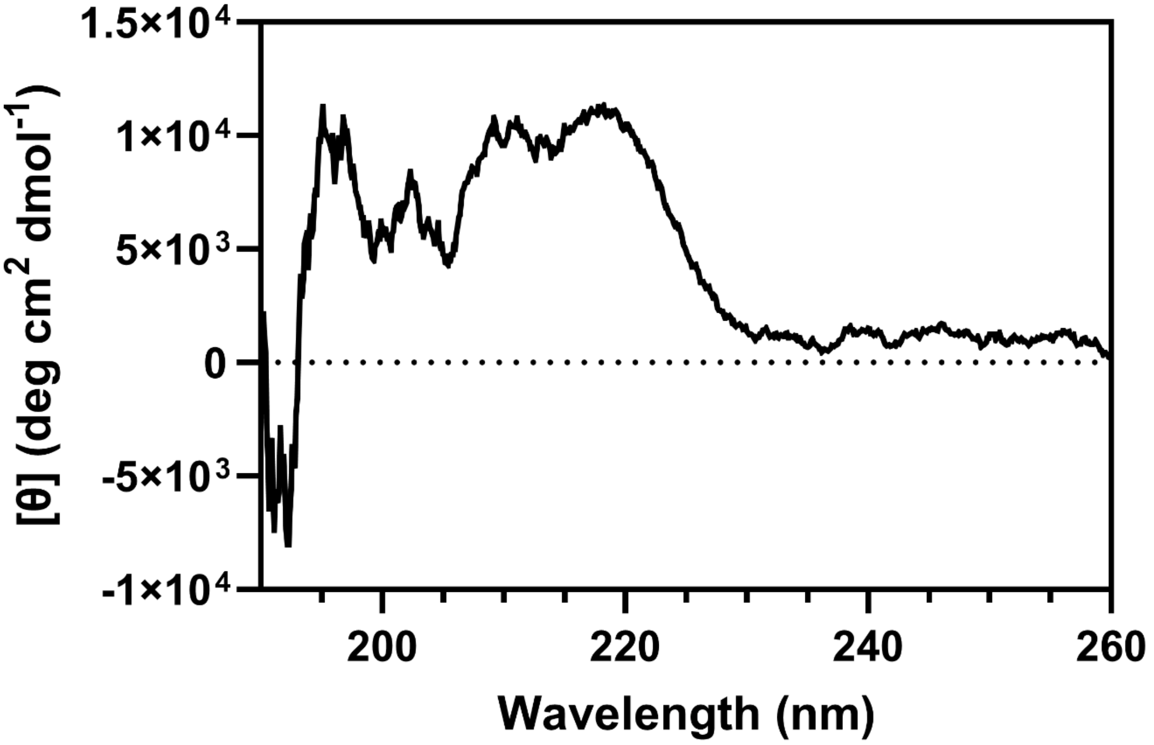

Figure S83. CD spectrum of self-assembled **L5** (2 mM) at 0 hours after assembly.

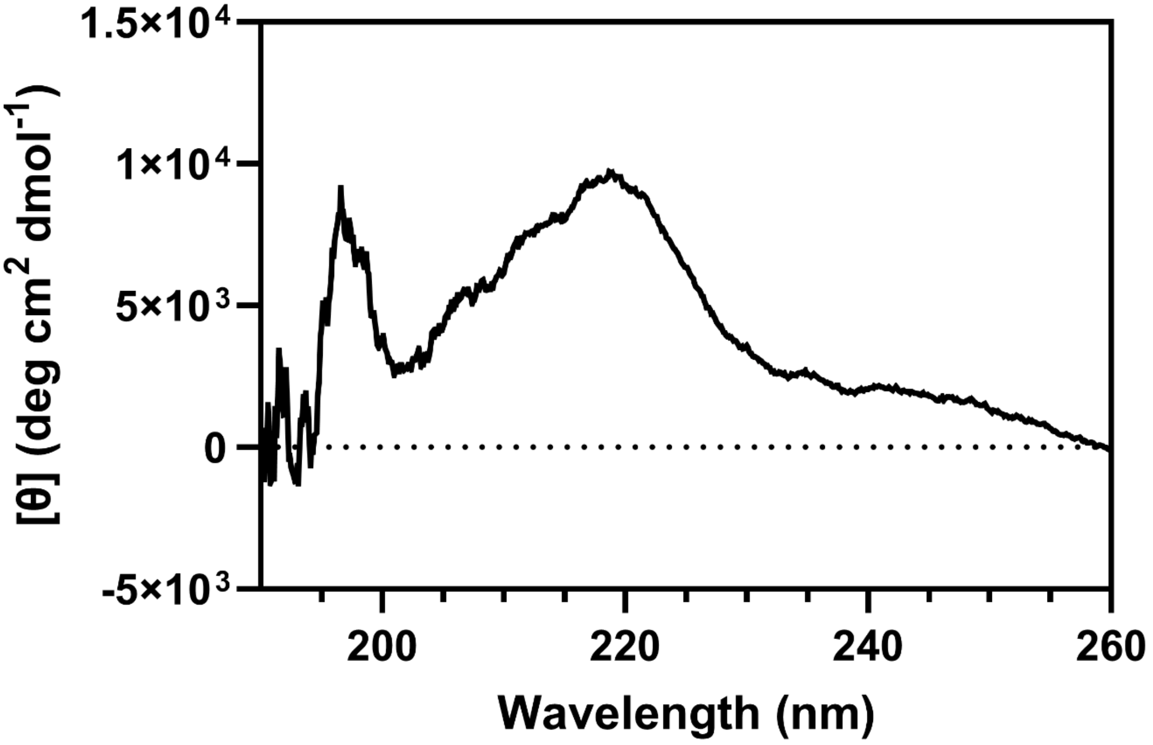

Figure S84. CD spectrum of self-assembled **L5** (4 mM) at 0 hours after assembly.

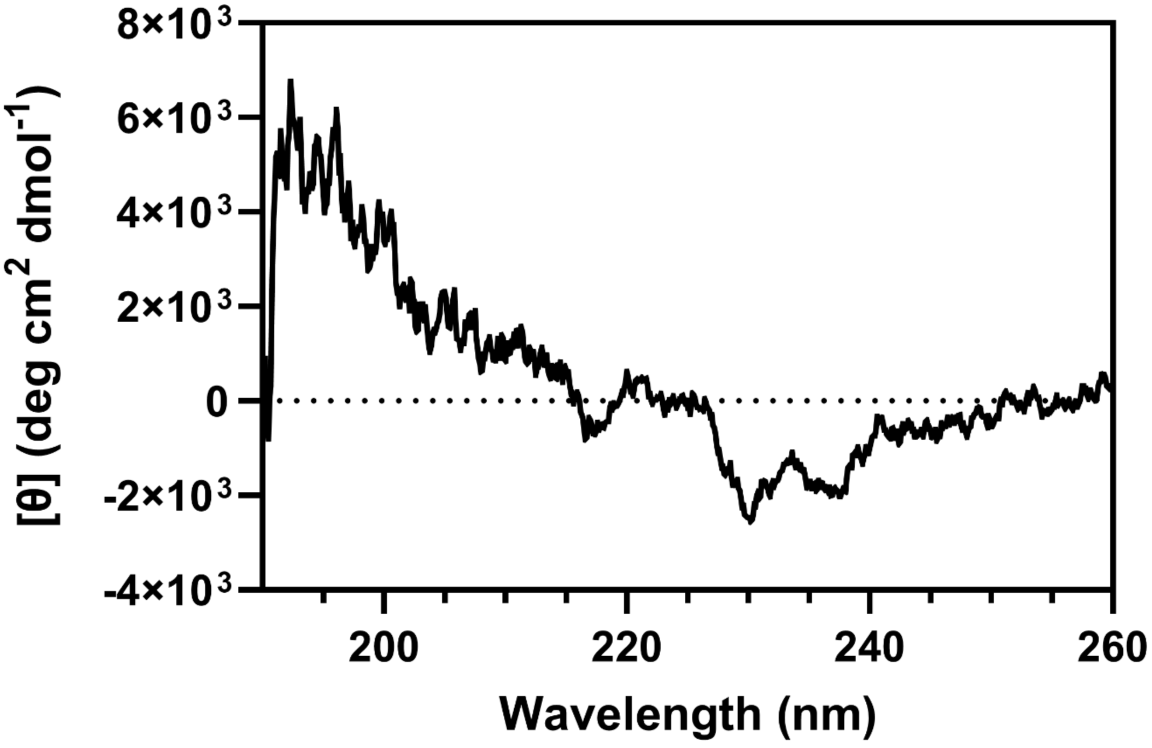

Figure S85. CD spectrum of coassembled **L5/D5** (1 mM) at 0 hours after assembly.

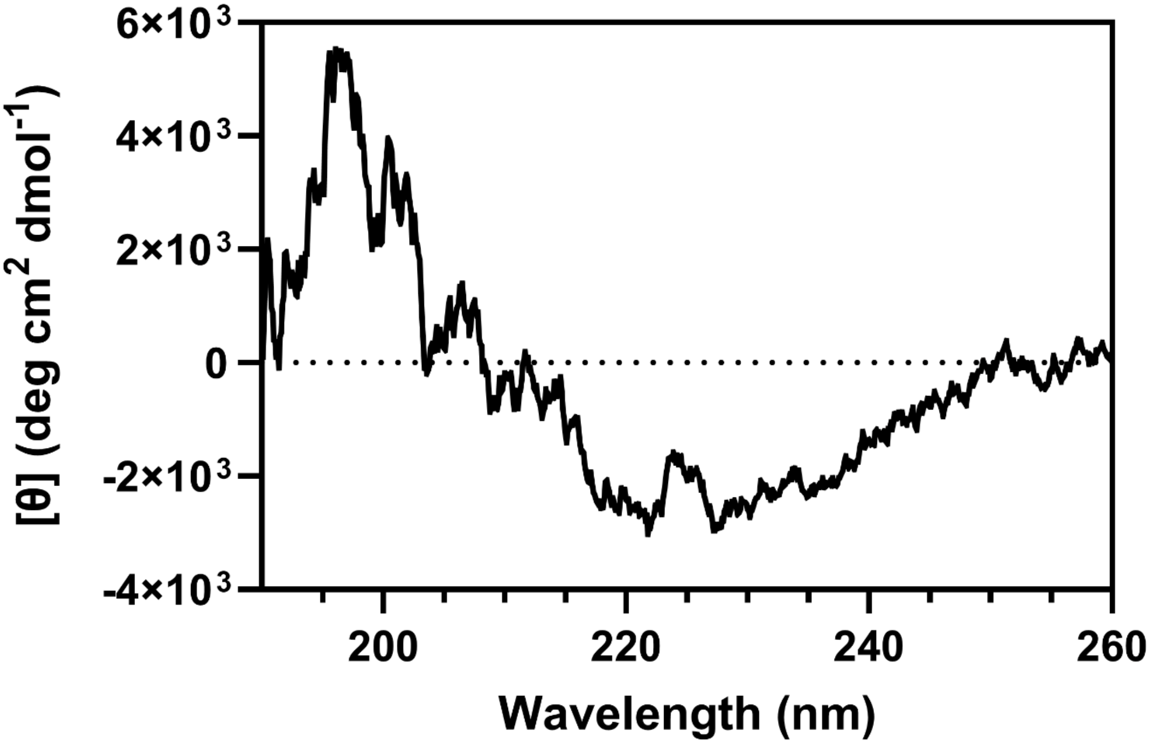

Figure S86. CD spectrum of coassembled **L5/D5** (2 mM) at 0 hours after assembly.

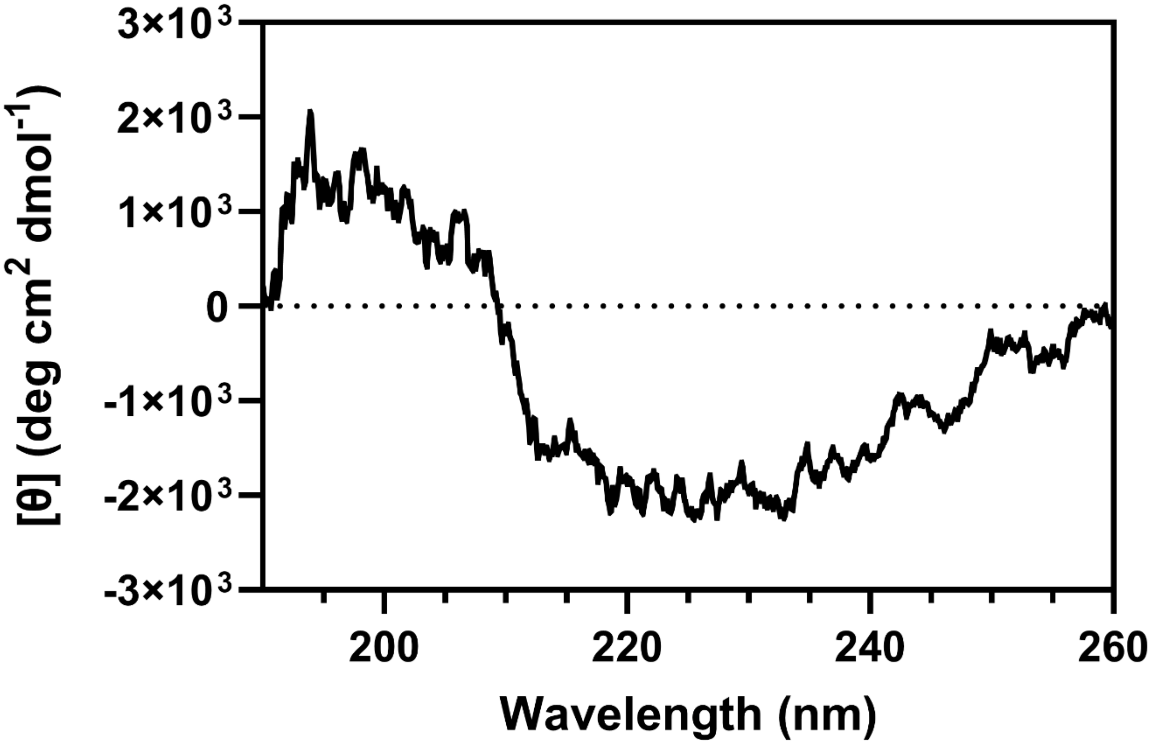

Figure S87. CD spectrum of coassembled **L5/D5** (4 mM) at 0 hours after assembly.

Table S10. CD Assembly and Working Concentrations

Sample (Assembly Concentration)	Working Concentration
**L1** (1 mM)	0.5 mM
**L1/D1** (1 mM)	0.5 mM
**L2** (1 mM)	0.57 mM
**L2/D2** (1 mM)	1 mM
**L3** (1 mM)	0.5 mM
**L3** (2 mM)	0.57 mM
**L3** (4 mM)	0.857 mM
**L3/D3** (1 mM)	0.5 mM
**L4** (1 mM)	1 mM
**L4** (2 mM)	1 mM
**L4** (4 mM)	1 mM
**L4/D4** (1 mM)	1 mM
**L4/D4** (2 mM)	1 mM
**L4/D4** (4 mM)	1 mM
**L5** (1 mM)	0.5 mM
**L5** (2 mM)	0.57 mM
**L5** (4 mM)	1.143 mM
**L5/D5** (1 mM)	0.5 mM
**L5/D5** (2 mM)	0.57 mM
**L5/D5** (4 mM)	0.857 mM

J-parameter

As defined in the main text, the *J*-parameter quantifies the difference between the number of hydrogen bonds formed between peptides of the same chirality (L–L and D–D) and those formed between peptides of opposite chirality (L–D). It is used to assess whether peptides preferentially interact with same-handed or mirror-image enantiomers in solution. However, the final assembled structures are influenced not only by energetic factors but also by stochastic effects, such as the initial spatial arrangement of peptides in the simulations. The impact of these random factors on the *J*-parameter can be assessed by analyzing systems in which peptide–peptide interactions are intrinsically unbiased with respect to chirality.

To this end, we analyzed homochiral simulations consisting of 10 L-peptides, each simulated for 2 μs using the same protocol as described in the main text. In these simulations, chirality labels were assigned by randomly designating five peptides as L and the remaining five as D, followed by computation of the *J*-parameter averaged over the final 100 ns of the trajectory. Because this labeling is arbitrary and does not correspond to physical differences between peptides, the resulting *J*-parameter reflects a system without any intrinsic chiral preference.

Different labeling assignments yield different J-values, and the full dispersion can be obtained from all $\frac{1}{2}\binom{10}{5} = 126$ distinct ways of assigning five L- and five D-peptides.

This procedure was applied to two independent simulations of peptide sequences L1, L2, and L3, yielding a total of six simulations. The resulting distribution of J-parameters is shown in **Figure S88**. The mean value of the distribution is −2.47, close to zero as expected for unbiased interactions, with a root-mean-square deviation of approximately 15. These results indicate that systems in which L-peptides preferentially interact with D-peptides are expected to exhibit a negative mean J-parameter, although individual simulations may still yield positive values due to statistical fluctuations. Consequently, meaningful interpretation of the J-parameter requires statistical analysis based on distributions obtained from multiple independent simulations. In this study, we were unable to perform more than 4 simulations for each L/D simulation because of the large size of our systems (~100,000 atoms) and the long simulation times (up to 3 μs), which require significant computational resources.

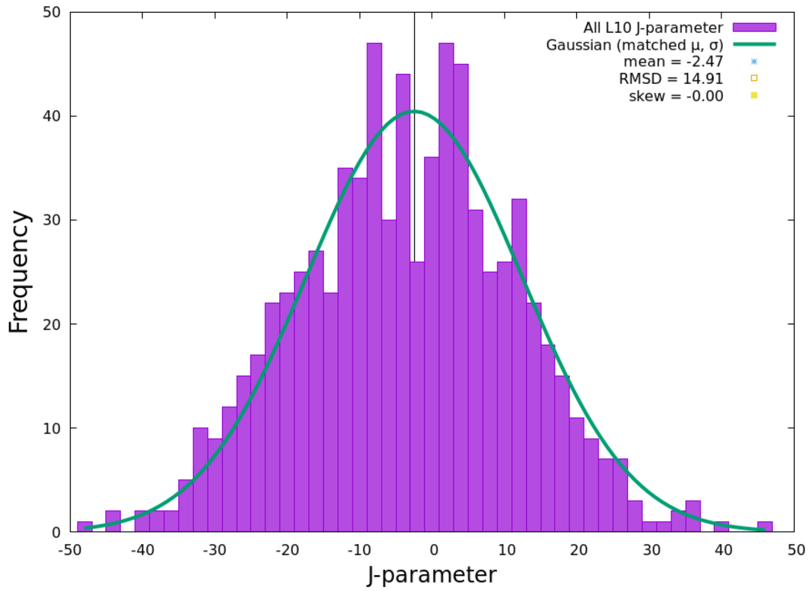

Figure S88. Distribution of the J-parameter for systems where peptides interact without bias based on chirality.
